# Financial Incentives for COVID-19 Vaccination: A Cluster Randomized Clinical Trial

**DOI:** 10.1001/jamanetworkopen.2024.58542

**Published:** 2025-02-07

**Authors:** John Ternovski, Sebastian Jilke, Florian Keppeler, Dominik Vogel

**Affiliations:** 1 Office of Labor and Economic Analysis, Department of Economics and Geosciences, Air Force Academy, Colorado Springs, Colorado; 2 McCourt School of Public Policy, Georgetown University, Washington, DC; 3 Department of Political Science, Aarhus University, Aarhus, Denmark; 4 Department of Political and Social Sciences, Zeppelin University, Friedrichshafen, Germany; 5 Department of Administrative Sciences, Harz University of Applied Sciences, Halberstadt, Germany

## Abstract

**Importance:**

Prior studies found that financial incentives have small, positive direct effects in increasing COVID-19 vaccination rates, but unmeasured social spillovers (ie, changes in outcomes among untreated individuals who are socially exposed to policy beneficiaries) may diminish the overall effect of such policies.

**Objective:**

To assess the spillover effects of a COVID-19 vaccination financial incentive and assess whether incorporating estimates of spillover meaningfully affects broader evaluations of policy effectiveness.

**Design, Setting, and Participants:**

This population-level, address-cluster randomized clinical trial was conducted in November 2021. Participants were all adult (aged ≥18 years) residents of Ravensburg, Germany, who were randomly assigned to the treatment or control group. One resident in each address cluster was randomly selected to be an address-cluster representative. Address-cluster representatives in the treatment group received the treatment letter; all other cohabitants at that same address received the control letter. All individuals in addresses randomly assigned to the control group were mailed a control letter. Intention-to-treat data analysis was conducted from January 2022 to May 2024.

**Intervention:**

Control letters informed recipients about 7 upcoming free COVID-19 vaccination events. Treatment letters were identical to control letters, except they also offered €40 (US $41.46) for getting vaccinated at one of the events.

**Main Outcome and Measure:**

Primary and booster COVID-19 vaccination uptake was observed and recorded on site during the public vaccination events. Primary vaccinations were defined as either the first dose of a 1-dose vaccine or the first or second dose of a 2-doses vaccine. Boosters were defined as any dose after primary vaccination. Three types of commonly used treatment effects were analyzed: direct, spillover, and overall.

**Results:**

Among 41 548 Ravensburg residents (mean [SD] age, 49.96 [19.04] years; 51.3% female), 796 (1.9%) were vaccinated at 1 of the 7 public vaccination events. The direct, spillover, and overall effects of receiving a financial incentive on primary vaccinations were all nonsignificant. For booster vaccinations, the direct effect was negative but not statistically significant (−0.32 percentage points [95% CI, −0.77 to 0.14 percentage points]; *P* = .17), whereas the overall effect (−0.30 percentage points [95% CI, −0.51 to −0.09 percentage points]; *P* = .006) was significantly negative. The spillover effect was significantly negative (−0.29 percentage points [95% CI, −0.53 to −0.06 percentage points]; *P* = .01), but only for the first vaccination events.

**Conclusions and Relevance:**

This trial found null direct effects on COVID-19 vaccination uptake and negative effects on booster uptake among individuals who did not receive but were indirectly exposed to the financial incentives. The timing of this spillover suggests that cohabitants of financial incentive recipients postponed booster vaccination, thereby undermining the potential effectiveness of this policy.

**Trial Registration:**

ISRCTN identifier: ISRCTN59503725

## Key Points

**Question:**

Are there adverse consequences of using financial incentives to encourage COVID-19 vaccination?

**Findings:**

In a cluster randomized clinical trial involving the entire adult population (n = 41 548) of a mid-sized German city, financial incentives had no direct impact on participants’ likelihood to get vaccinated against COVID-19 (first dose, second dose, or booster). Individuals living in the same household as financial incentive recipients were significantly less likely to receive booster vaccination compared with the control group.

**Meaning:**

Findings from this study suggest that financial incentives may have adverse social spillover effects on vaccination rates and that spillover effects should be measured in randomized clinical trials to avoid costly, ineffective, or counterproductive policy outcomes.

## Teaser

This cluster randomized clinical trial evaluates the direct, spillover, and overall effects of giving adults a financial incentive to receive primary or booster COVID-19 vaccination.

## Introduction

Policymakers have increasingly leveraged behavioral science to bridge medical research and policy^1,2^ (eAppendix 12 in Supplement 2). However, when behavioral science interventions are scaled up, social spillovers and delayed effects could lead to adverse policy outcomes.^3^ Social spillovers are defined as any changes in outcomes among individuals who are not direct beneficiaries of a policy but are socially exposed to policy beneficiaries. Delayed effects are any policy outcomes that occur after initial outcomes are collected. If these effects are left unmeasured, ineffective or harmful policies may falsely appear to be effective. We illustrated this risk via a cluster randomized clinical trial (RCT) that evaluated a popular^4,5^ intervention to encourage vaccination against COVID-19. There is extensive literature^6,7,8,9,10^ on the use of monetary incentives to improve health-related behaviors; a prominent RCT found that offering approximately $24 to online survey takers increased COVID-19 vaccination by 4 percentage points.^11^ The same research team conducted follow-up work, addressing the possibility that financial incentives could lead to backlash in the future, and concluded that “financial incentives for vaccination do not have negative unintended consequences.”^12^ However, as is common practice in policy RCTs, neither the original nor the expanded study measured social spillover. Across a meta-analysis of 11 RCTs^13^ and a systematic review of all quantitative studies that evaluated the effectiveness of financial incentives for COVID-19 vaccination (38 studies),^14^ all included studies measured only direct effects. Both the meta-analysis and systematic review concluded that financial incentives were associated with small, positive increases in COVID-19 vaccination.^13,14^

However, prior research has indicated that social spillover may affect vaccination interest and confirmed vaccinations^15,16,17^ and that spillover is not always positive.^15^ There are also theoretical reasons to suspect that financial incentives may crowd out (ie, replace) prosocial behaviors such as vaccination.^18,19,20^ In this RCT, we aimed to assess the effect of incorporating estimates of spillover on broader evaluations of policy effectiveness. Specifically, we performed a comprehensive test of a policy intervention (similar to that used in Campos-Mercade et al^11^ but across the full population of a mid-sized city rather than a sample of paid survey panelists). This study aims to highlight the importance of measuring social spillover and delayed effects before a program is scaled up.

## Methods

### Trial Design

This report follows the Consolidated Standards of Reporting Trials (CONSORT) reporting guideline,^21^ and the original trial protocol is provided in Supplement 1. This 2-arm parallel cluster RCT was initiated in November 2021. The unit of randomization was residency address, with all registered adult residents of the city of Ravensburg, Germany, included. The Zeppelin University Ethics Committee approved this study and waived participants’ informed consent for inclusion in the study because the vaccination campaign was to be conducted by local government officials and the study was designed to evaluate this campaign.

After the trial commenced, specifically during the first vaccination event, vaccination demand was observed exceeding initial expectations, and the city decided to organize 11 additional vaccination events. These additional events were not mentioned in either the treatment or control letters sent to residents and occurred between December 1 and 18, 2021. We updated our preregistration plan when the city informed us of the possibility of additional events (November 12, 2021). The additional outcome data from these unannounced events made it possible to measure delayed effects.

### Participants and Sample Size

The eligible population was drawn from the official city registry, which includes all adults who legally reside in Ravensburg. All vaccination events were conducted in strict adherence to German public health authorities’ vaccination procedures, which include consultation with a physician and signed consent from the participant to get vaccinated. The Zeppelin University Ethics Committee waived participants’ informed consent not only for their inclusion in the study but also for the use of their data in the city’s administrative registry and the use of their in-person vaccination consent observed at the public vaccination events (eAppendix 9 in Supplement 2).

The sample size was determined by the total number of legal residents in the city registry. All demographic information was obtained from the registry. Besides 3 preregistered exclusions (which totaled less than 1% of all residents and included a prison and an address that appeared to have 75 residents), the full population was included in the sample (eAppendix 10 in Supplement 2).

### Randomization

With a 1:1 allocation ratio, registered addresses (ie, clusters) were randomized to 2 experimental conditions. After stratifying by cluster size and blocking within each stratum (4 addresses per block) to ensure balance,^18,19^ all addresses were randomly assigned to 1 of 2 groups: treatment or control. One adult resident in each address was randomly selected as a cluster representative. The cluster representatives who resided in treatment addresses received the treatment letter, and all other individuals at the same address (cohabitants) received the control letter. All individuals in the control addresses were mailed a control letter. The randomization code is available as part of the replication package (eAppendix 8 in Supplement 2). One of us (D.V.) implemented the allocation and concealed it from the city team, who sent out the mailing.

### Intervention

The control and treatment letters were identical, except that the treatment letter offered recipients financial incentives for COVID-19 vaccination. Both letters were sent out at the same time, with residents receiving their letters in the first week of November 2021. The letters informed recipients of 7 public COVID-19 vaccination events occurring between November 13 and December 12, 2021. The letters were addressed from and signed by city officials and the mayor of Ravensburg. The full timeline of the study, including changes to German vaccination policies, is provided in eAppendix 3 in Supplement 2.

German health authorities recommended booster vaccinations for all persons 18 years or older on November 5, 2021. Mailed letters are the typical mode of communication between German government organizations and residents.^22^ During this time in Germany, the availability of COVID-19 vaccines and vaccination appointments for the general public were limited. Public vaccination events were intended to ameliorate this issue, especially as the start of the booster campaign created a considerable demand wave. Public vaccination events operated on a first-come first-served basis.

The treatment letter offered participants in the treatment group a total of €40 (US $41.46) for getting vaccinated. Specifically, there were 2 financial incentives. The individual-level incentive (modeled after the financial incentive in a study by Campos-Mercade et al^11^) offered recipients €20 ($20.73) for getting vaccinated at 1 of the 7 public vaccination events. To encourage positive social spillovers, we offered an additional community-level incentive: if the treatment letter recipient did get vaccinated, they could receive an additional €20 if more than 900 Ravensburg residents got vaccinated at the 7 public events. The letter also specified that if the recipient was already fully vaccinated, they could still receive the community-level incentive if they transferred their treatment letter to another Ravensburg resident and if this other individual presented the letter while getting vaccinated at one of the events. All letters presented to city officials were retained, and financial incentives were issued to participants only after data analysis.

The full letters, translated into English, are provided in eAppendix 1 in Supplement 2. The letters contained a QR (quick response) code with information in English, Spanish, Russian, Arabic, and Turkish, which, together with German, are 6 of the most commonly spoken languages in Ravensburg. Ravensburg's city registry includes nationality, which represents the official nationality of the resident as determined by the German government; the non-German nationality group includes 130 different nationalities. Nationality was used in this analysis to test for heterogeneous treatment effects.

**Figure 1.**
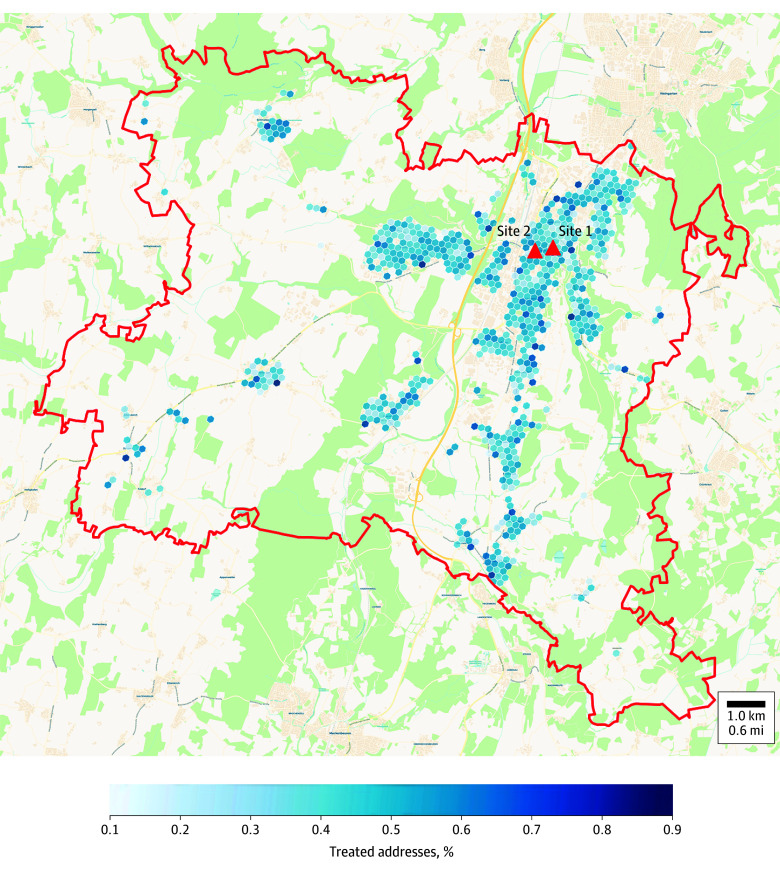
Geographic Distribution of Participants Around Vaccination Sites The map depicts the location of the vaccination sites in relation to the population of study participants in the city of Ravensburg (red line indicates the city’s border). Fifty percent of participants were within 1.8 miles (2.9 km) of the vaccination sites, and 80% were within 3.4 miles (5.5 km) of the vaccination sites.

All vaccination sites were centrally located (Figure 1), less than 100 m (0.06 miles) from the central public bus station, and in fully accessible, well-signposted buildings with ample nearby parking. Some of the medical staff at the public vaccination events were multilingual to assist non-German speakers. The vaccine was free of charge, and vaccination did not require participants to have health insurance or identity papers.

### Outcomes

The main outcome was observed primary and booster COVID-19 vaccination uptake, as estimated via direct, spillover, and overall effects. Vaccination was observed by city officials and recorded on site during the public vaccination events. Vaccination dose number (eg, first, second) and prior vaccination details were self-reported by participants and recorded by city officials. Primary vaccinations were defined as either the first dose of a 1-dose vaccine (eg, Ad26.COV2.S; Johnson & Johnson) or the first or second dose of a 2-doses vaccine (eg, mRNA-1273; Moderna). Boosters were defined as any dose after primary vaccination.

Names and addresses were linked to city administrative records and verified on site. Collected outcome data were later transferred to the study team to match against treatment assignment. An additional outcome was whether a participant visited the informational website mentioned in the control and treatment letters, as estimated via direct and overall effects (eAppendix 2 in Supplement 2).

### Statistical Analysis

Analysis was conducted according to the preregistered analysis plan (eAppendix 10 in Supplement 2). We estimated 3 types of commonly used^23^ treatment effects: direct, spillover, and overall (eAppendix 4 in Supplement 2). The direct effect was assessed by comparing the treated individuals in the treatment address clusters with those individuals who would have been treated in the control address clusters (ie, address cluster representatives). The spillover effect was assessed by comparing the untreated individuals in treatment address clusters with the individuals in the control address clusters who were not randomly selected as address-cluster representatives. The overall effect was assessed by comparing all participants in the treatment address clusters with those of the control address clusters. Delayed effects were measured by estimating the direct, overall, and spillover effects for each vaccination event day and for announced vaccination events vs additional unannounced vaccination events.

As is standard practice^24,25^ for direct treatment effects, we used covariate-adjusted (age, age^2^ [to account for a nonlinear association between age and vaccination], female sex, non-German nationality) ordinary least squares regression with a set of indicators for randomization blocks and heteroskedasticity robust SEs. For spillover and overall treatment effects, we clustered SEs by address clusters. The results were robust to alternative specifications, including randomization inference^26^ (eAppendix 6 in Supplement 2). The spillover analysis assumed that individuals living at the same address were in the same social network (for alternative social network operationalizations, see eAppendix 6 in Supplement 2).

Two-sided *P* < .05 indicated statistical significance. Intention-to-treat data analysis was conducted from January 2022 to May 2024 using R, version 4.4.1 (R Core Team).

## Results

**Figure 2.**
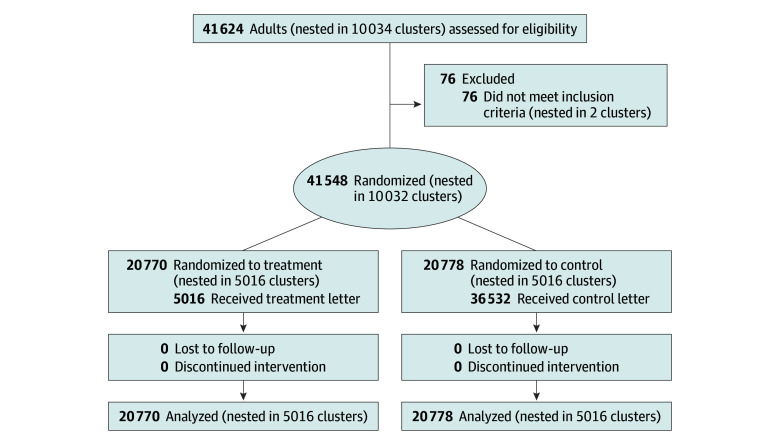
CONSORT Diagram

We analyzed the entire adult registered population of the city of Ravensburg, including 41 548 individuals at 10 032 addresses (Figure 2). These residents had a mean (SD) age of 49.96 (19.04) years; 21 326 (51.3%) were women and 20 222 (48.7%) were men; 35 181 (84.7%) were German nationals, and 6367 (15.3%) were of other nationalities (Table).

**Table.** Baseline Characteristics of Participants by Treatment Condition^a^

Characteristic	Participants, No. (%)				
	Overall (N = 41 548)	Treatment arm		Control arm	
		Cluster representatives (n = 5016)	Cohabitants (n = 15 754)	Cluster representatives (n = 5016)	Cohabitants (n = 15 762)
Mean (SD) cluster size	8.58 (8.30)	4.14 (4.28)	9.99 (8.71)	4.14 (4.30)	10.01 (8.79)
Age, mean (SD), y	49.96 (19.04)	53.22 (19.05)	49.03 (18.84)	53.06 (19.13)	48.85 (18.99)
Sex					
Female	21 326 (51.3)	2596 (51.8)	8010 (50.8)	2585 (51.5)	8135 (51.6)
Male	20 222 (48.7)	2420 (48.2)	7744 (49.2)	2431 (48.5)	7627 (48.4)
Nationality^b^					
German	35 181 (84.7)	4531 (90.3)	13 131 (83.4)	4552 (90.7)	12 967 (82.3)
Other^b^	6367 (15.3)	485 (9.7)	2623 (16.6)	464 (9.3)	2795 (17.7)
Vaccinated at event					
Yes	796 (1.9)	87 (1.7)	283 (1.8)	99 (2.0)	327 (2.1)
No	40 752 (98.1)	4929 (98.3)	15 471 (98.2)	4917 (98.0)	15 435 (97.9)
Primary vaccination at event					
Yes	307 (0.7)	27 (0.5)	129 (0.8)	23 (0.5)	128 (0.8)
No	41 241 (99.3)	4989 (99.5)	15 625 (99.2)	4993 (99.5)	15 634 (99.2)
Booster vaccination at event					
Yes	489 (1.2)	60 (1.2)	154 (1.0)	76 (1.5)	199 (1.3)
No	41 059 (98.8)	4956 (98.8)	15 600 (99.0)	4940 (98.5)	15 563 (98.7)

^a^ For balance tests, see eAppendix 6 in Supplement 2.

^b^ Ravensburg’s city registry includes nationality, which represents the official nationality of the resident as determined by the German government; the other nationality group includes 130 different nationalities.

**Figure 3.**
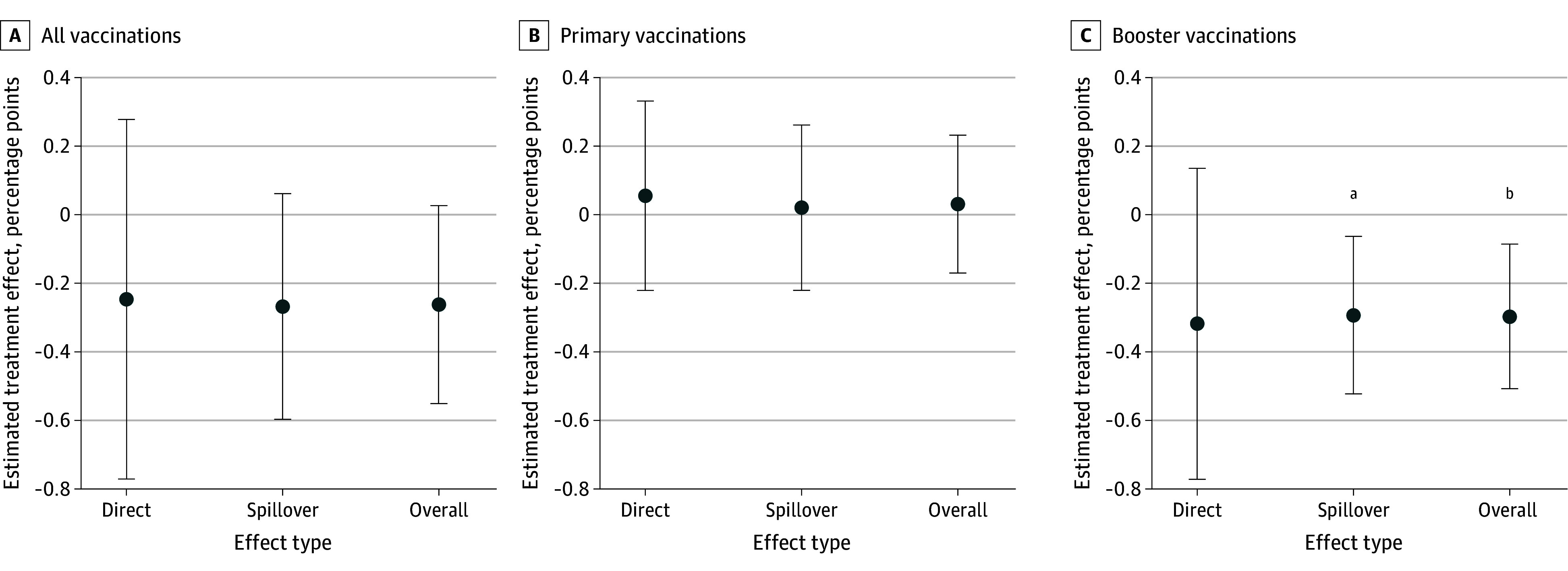
Estimated Treatment Effects on All Vaccinations, Primary Vaccinations (First and Second Doses), and Booster Vaccinations Models included prespecified demographic characteristics and a set of randomization-block indicators as covariates; unadjusted results were nearly identical (eAppendix 11 in Supplement 2). Error bars represent 95% CIs. ^a^*P* < .05. ^b^*P* < .01.

A total of 796 of 41 548 individuals (1.9%) were vaccinated at 1 of the 7 announced public vaccination events. The covariate-adjusted treatment effects are displayed in Figure 3 in a coefficient plot with 95% CIs (for regression table, see eAppendix 11 in Supplement 2). The direct effect of receiving a financial incentive on all vaccinations was directionally negative (−0.25 percentage points [95% CI, −0.77 to 0.28 percentage points]; 87 of 5016 in the treatment group and 99 of 5016 in the control group), although this result was not statistically significant (*P* = .36). Similarly, the spillover effect of the financial incentive on all vaccinations was statistically nonsignificant (−0.27 percentage points [95% CI, −0.60 to 0.06 percentage points]; 283 of 15 754 in the treatment group and 327 of 15 762 in the control group; *P* = .11). The overall effect of being in an address cluster that received the financial incentive was also nonsignificant (−0.26 percentage points; 95% CI, −0.55 to 0.03 percentage points; 370 of 20 770 in the treatment group and 426 of 20 778 in the control group; *P* = .08) (Figure 3A). Panels B and C in Figure 3 present the results for primary and booster vaccinations separately (for regression tables, see eAppendix 11 in Supplement 2). Effects on first doses were not meaningfully different from the effects on primary doses (ie, first or second doses for the 2-dose vaccines).

We found a statistically significant negative spillover effect for booster vaccinations (−0.29 percentage points [95% CI, −0.53 to −0.06 percentage points]; 154 of 15 754 in the treatment group and 199 of 15 762 in the control group; *P* = .01). In other words, untreated cohabitants in a treatment address cluster were less likely to get vaccinated at publicly announced vaccination events than their counterparts in a control address cluster. The direct effect was negative but not statistically significant (−0.32 percentage points [95% CI, −0.77 to 0.14 percentage points]; 60 of 5016 in the treatment group and 76 of 5016 in the control group; *P* = .17), while the overall effect was significantly negative (−0.30 percentage points [95% CI, −0.51 to −0.09 percentage points]; 214 of 20 770 in the treatment group and 275 of 20 778 in the control group; *P* = .006), indicating that individuals in treatment address clusters were less likely to receive a booster vaccination than residents in control address clusters. For statistical power and sensitivity analyses of these effects, see eAppendix 7 in Supplement 2.

**Figure 4.**
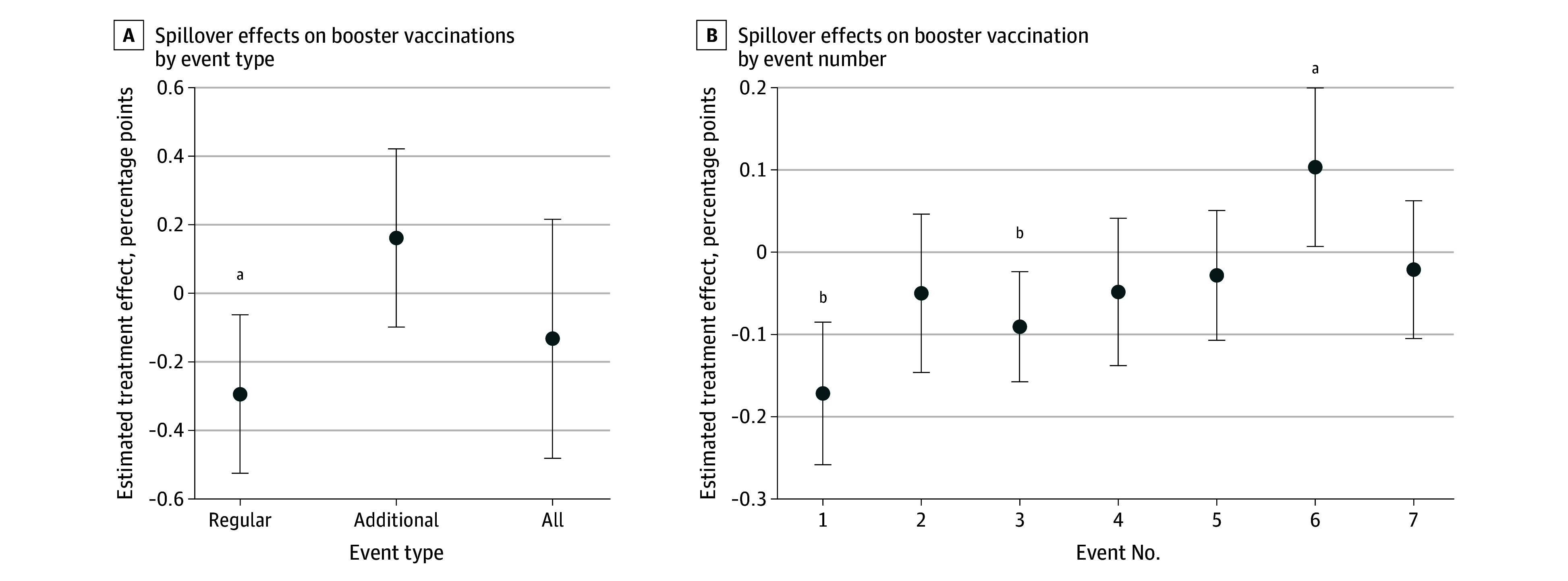
Estimated Social Spillover Effects of Financial Incentives on Booster Vaccinations at Regular, Additional, and All Events and Vaccinations by Event Number This figure identifies at what point in time the negative social spillover occurred. Regular events 1 to 5 occurred before the additional events, and 7 of 11 additional events occurred after the last announced regular event (eAppendix 3 in Supplement 2). For each event, a successful vaccination was defined as occurring only during that event; all other vaccinations (including future vaccinations) were coded as an absence of a vaccination during that event. Models included prespecified demographics and a set of randomization-block indicators as covariates; unadjusted results were nearly identical (eAppendix 6 in Supplement 2). Error bars represent 95% CIs. ^a^*P* < .05. ^b^*P* < .01.

To investigate the intersection of delayed effects and social spillover (ie, when did social spillover occur?), we limited the analysis to vaccinations at the unannounced, additional events (ie, events that occurred predominantly after the announced events). Figure 4A shows a directionally positive spillover effect on booster vaccinations of a similar magnitude to the negative spillover effect (0.16 percentage points [95% CI, −0.10 to 0.42 percentage points]; 224 of 15 469 in the treatment group and 200 of 15 435 in the control group), although this effect was not significant (*P* = .22). When we reran the main analysis with vaccinations at additional events as affirmative vaccinations, the negative spillover effect on boosters disappeared (−0.13 percentage points [95% CI, −0.48 to 0.22 percentage points]; 378 of 15 754 in the treatment group and 399 of 15 762 in the control group; *P* = .46). Additionally, the first 3 events had long waiting lines, and, when treatment effects by event were investigated for delayed effects, we found that the significant negative spillover was localized entirely in the first event (Figure 4B). For additional analyses of delayed effects, see eAppendix 5 in Supplement 2.

## Discussion

Consistent with recent results,^27,28^ this study found that a citywide financial incentive that was randomly allocated to individual households did not significantly increase COVID-19 vaccination rates. When examining only booster vaccinations, a small negative social spillover effect was identified. Analysis of additional events and event-by-event regression suggested that the negative spillover may have been temporary and may be a displacement of COVID-19 vaccinations from advertised events to future unadvertised events. The results illustrate the presence of spillovers and the related pitfalls of incomplete outcome data when evaluating the effectiveness of policy interventions. A random representative sample may produce accurate estimates of direct effects, but most random samples do not capture spillover effects—within a household, for instance. If we measured only direct effects, we may have falsely concluded that financial incentives, at least, did no harm in Ravensburg. By examining social spillover and delayed effects, we identified a small but significant negative effect of financial incentives, which illustrates that a seemingly benign intervention could still lead to adverse policy outcomes.

Missingness of outcome data played an important role in interpreting this unexpected result. Since the city decided to host additional COVID-19 vaccination events that were not mentioned in the letters sent to study participants, we were able to analyze vaccination outside of announced events. Although we saw negative spillover effects for vaccination during advertised events, we also saw positive (though nonsignificant) spillover effects during unadvertised events. The fact that the first advertised events had long lines and latecomers were turned away due to limited vaccine doses may mean that individuals living at the same address as a recipient of the financial incentive assumed that most attendees at the advertised events were there for the financial incentive. Thus, to avoid long lines, these individuals may have postponed vaccination. There is only suggestive evidence of this interpretation given that the positive social spillover effect in later events was not statistically significant.

The null direct effect was also unexpected and could be a ceiling effect, as the city reached a vaccination rate of 70%, which, as prior research found,^29^ was the proportion of people willing to be vaccinated voluntarily in Germany. Another explanation could be that the German-speaking population had lower rates of health literacy and higher rates of conspiracy theory beliefs than residents in other countries.^30,31^ Alternatively, the financial incentives offered may have been too low^32^ or simply ineffective in this context. Individual-level incentives are often effective when the intrinsic benefit of getting vaccinated is only slightly lower than the individual cost of vaccination. However, with the rapid surge in infections and hospitalizations in November 2021, we observed long lines during the initial events; nearly half of all attendees of the events were from outside the city, and most vaccinations during the events were boosters. This observation may indicate that the perceived benefit of getting vaccinated may have already been greater than the perceived cost. Relatedly, individuals may seek to subsidize the costs of a behavior with highly uncertain benefits (as with an experimental drug); at this point in the pandemic in Germany, many individuals may have perceived the benefits of vaccination as substantially more certain. The individual-level incentive may have simply been unnecessary for those who already intended to be vaccinated. Conversely, the lack of effect of the individual-level incentive on first doses may mean that vaccine-hesitant individuals required much higher compensation than what was offered. There were similar takeaways for the community-level incentive, although the community-level incentive may have been too complicated to have widespread positive spillovers and/or the community-level goal may have been perceived as too high (although the goal of 900 vaccinations was ultimately reached).

### Limitations

This trial has some limitations. First, the public events were not the only options for vaccination, so it is possible some vaccination outcomes were not captured in this analysis; however, at the time of this study, vaccine doses and vaccination appointments were extremely limited. Second, vaccinations that occurred after the last vaccination event (December 30, 2021) were not captured. Third, we were not able to measure what proportion of the Ravensburg population opened, read, and understood the treatment and control letters informing them of the public vaccination events.

## Conclusions

This cluster RCT of financial incentives for vaccination illustrates the importance of measuring spillovers from health policy interventions to identify potential unintended consequences before a policy is implemented at scale. The negative social spillover effect found here is a conservative estimate. Since there was compelling preexisting evidence of the possibility of negative social spillover from individual-level financial incentives,^25^ we incorporated community-level incentives in the treatment instead of just replicating Campos-Mercade et al^11^ on a population level. The community-level incentive was designed to ameliorate the possibility of negative spillovers. The fact that we see any negative spillover from the combination of individual-level and community-level incentives should be viewed as a cautionary note regarding the use of monetary incentives in this context. Additionally, the existence of other public health contexts that require immediate community action (such as in the early stages of a burgeoning epidemic) may mean that postponement of a health behavior could have consequences for containment efforts.^33,34^ Therefore, we recommend incorporating measures of spillover into RCTs before a policy is implemented at scale.

## Notes

Supplement 1.
Trial Protocol

Supplement 2. **eAppendix 1.** Treatment and Control Letters **eAppendix 2.** Data Collection **eAppendix 3.** Timeline of Experiment and Relevant Policy Changes **eAppendix 4.** Types of Treatment Effects **eAppendix 5.** Investigating Postponement Spillovers **eAppendix 6.** Robustness Checks **eAppendix 7.** Statistical Power and Sensitivity Analysis **eAppendix 8.** Data and Code Availability **eAppendix 9.** Ethics **eAppendix 10.** Pre-registered Analysis Plan **eAppendix 11.** Additional Analyses **eAppendix 12.** The Development and Prevalence of Evidence-Based Policy-Making
eReferences

Supplement 3.
Data Sharing Statement

## References

[1] Venkataramani AS, Underhill K, Volpp KG. Moving toward evidence-based policy: the value of randomization for program and policy implementation. JAMA. 2020;323(1):21-22. doi:10.1001/jama.2019.18061 31730191

[2] Cairney P, Oliver K. Evidence-based policymaking is not like evidence-based medicine, so how far should you go to bridge the divide between evidence and policy? Health Res Policy Syst. 2017;15(1):35. doi:10.1186/s12961-017-0192-x 28446185 PMC5407004

[3] List JA. Optimally generate policy-based evidence before scaling. Nature. 2024;626(7999):491-499. doi:10.1038/s41586-023-06972-y 38356064

[4] Böhm R, Betsch C, Litovsky Y, . Crowdsourcing interventions to promote uptake of COVID-19 booster vaccines. EClinicalMedicine. 2022;53:101632. doi:10.1016/j.eclinm.2022.101632 36090456 PMC9444232

[5] Serra-Garcia M, Szech N. Incentives and defaults can increase COVID-19 vaccine intentions and test demand. Manage Sci. 2023;69(2):1037-1049. doi:10.1287/mnsc.2022.4405

[6] Mantzari E, Vogt F, Shemilt I, Wei Y, Higgins JPT, Marteau TM. Personal financial incentives for changing habitual health-related behaviors: a systematic review and meta-analysis. Prev Med. 2015;75:75-85. doi:10.1016/j.ypmed.2015.03.001 25843244 PMC4728181

[7] Giles EL, Robalino S, McColl E, Sniehotta FF, Adams J. The effectiveness of financial incentives for health behaviour change: systematic review and meta-analysis. PLoS One. 2014;9(3):e90347. doi:10.1371/journal.pone.0090347 24618584 PMC3949711

[8] Vlaev I, King D, Darzi A, Dolan P. Changing health behaviors using financial incentives: a review from behavioral economics. BMC Public Health. 2019;19(1):1059. doi:10.1186/s12889-019-7407-8 31391010 PMC6686221

[9] Thirumurthy H, Asch DA, Volpp KG. The uncertain effect of financial incentives to improve health behaviors. JAMA. 2019;321(15):1451-1452. doi:10.1001/jama.2019.2560 30907936

[10] Facciorusso A, Demb J, Mohan BP, Gupta S, Singh S. Addition of financial incentives to mailed outreach for promoting colorectal cancer screening: a systematic review and meta-analysis. JAMA Netw Open. 2021;4(8):e2122581. doi:10.1001/jamanetworkopen.2021.22581 34432010 PMC8387849

[11] Campos-Mercade P, Meier AN, Schneider FH, Meier S, Pope D, Wengström E. Monetary incentives increase COVID-19 vaccinations. Science. 2021;374(6569):879-882. doi:10.1126/science.abm0475 34618594 PMC10765478

[12] Schneider FH, Campos-Mercade P, Meier S, Pope D, Wengström E, Meier AN. Financial incentives for vaccination do not have negative unintended consequences. Nature. 2023;613(7944):526-533. doi:10.1038/s41586-022-05512-4 36631607 PMC9833033

[13] Huang Y, Huang X, Yu R. The effectiveness of nonfinancial interventions and monetary incentives on COVID-19 vaccination: a meta-analysis. Health Psychol. 2023;42(6):411-424. doi:10.1037/hea0001288 37126030

[14] Khazanov GK, Stewart R, Pieri MF, . The effectiveness of financial incentives for COVID-19 vaccination: a systematic review. Prev Med. 2023;172:107538. doi:10.1016/j.ypmed.2023.107538 37156430 PMC10163939

[15] Bouckaert N, Gielen AC, Van Ourti T. It runs in the family—influenza vaccination and spillover effects. J Health Econ. 2020;74:102386. doi:10.1016/j.jhealeco.2020.102386 33147513

[16] Banerjee AV, Duflo E, Glennerster R, Kothari D. Improving immunisation coverage in rural India: clustered randomised controlled evaluation of immunisation campaigns with and without incentives. BMJ. 2010;340:c2220. doi:10.1136/bmj.c2220 20478960 PMC2871989

[17] Carpenter CS, Lawler EC. Direct and spillover effects of middle school vaccination requirements. Am Econ J Econ Policy. 2019;11(1):95-125. doi:10.1257/pol.20170067

[18] Frey BS, Oberholzer-Gee F. The cost of price incentives: an empirical analysis of motivation crowding-out. Am Econ Rev. 1997;87(4):745-755. https://www.jstor.org/stable/2951373

[19] Gneezy U, Meier S, Rey-Biel P. When and why incentives (don’t) work to modify behavior. J Econ Perspect. 2011;25(4):191-210. doi:10.1257/jep.25.4.191

[20] Gneezy U, Rustichini A. Pay enough or don’t pay at all. Q J Econ. 2000;115(3):791-810. doi:10.1162/003355300554917

[21] Schulz KF, Altman DG, Moher D; CONSORT Group. CONSORT 2010 statement: updated guidelines for reporting parallel group randomised trials. BMJ. 2010;340:c332. doi:10.1136/bmj.c332 20332509 PMC2844940

[22] German Federal Statistical Office. Type of communication with public authorities. Focus on the digitalization of the Federal Statistical Office’s life situation survey. 2021. Accessed August 12, 2024. https://www.amtlich-einfach.de/DE/Ergebnisse/Buerger2021/Digitalisierung/Digitalisierung_node.html

[23] Benjamin-Chung J, Arnold BF, Berger D, . Spillover effects in epidemiology: parameters, study designs and methodological considerations. Int J Epidemiol. 2018;47(1):332-347. doi:10.1093/ije/dyx201 29106568 PMC5837695

[24] Sorg ET, Haberman CP, Ratcliffe JH, Groff ER. Foot patrol in violent crime hot spots: the longitudinal impact of deterrence and posttreatment effects of displacement. Criminology. 2013;51(1):65-101. doi:10.1111/j.1745-9125.2012.00290.x

[25] Jilke S, Keppeler F, Ternovski J, Vogel D, Yoeli E. Policy makers believe money motivates more than it does. Sci Rep. 2024;14(1):1901. doi:10.1038/s41598-024-51590-x 38253624 PMC10803740

[26] Gerber AS, Green DP. Field experiments: design, analysis, and interpretation. 2012. Accessed August 12, 2024. https://external.dandelon.com/download/attachments/dandelon/ids/CH001CB8AFC4CB182BA9AC1257BC70035C7C0.pdf

[27] Milkman KL, Ellis SF, Gromet DM, . Megastudy shows that reminders boost vaccination but adding free rides does not. Nature. 2024;631(8019):179-188. doi:10.1038/s41586-024-07591-x 38926578 PMC11222156

[28] Chang TY, Jacobson M, Shah M, Kopetsky M, Pramanik R, Shah SB. Reminders, but not monetary incentives, increase COVID-19 booster uptake. Proc Natl Acad Sci U S A. 2023;120(31):e2302725120. doi:10.1073/pnas.2302725120 37487101 PMC10400997

[29] Graeber D, Schmidt-Petri C, Schröder C. Attitudes on voluntary and mandatory vaccination against COVID-19: evidence from Germany. PLoS One. 2021;16(5):e0248372. doi:10.1371/journal.pone.0248372 33970933 PMC8109805

[30] Fiske A, Schönweitz F, Eichinger J, . The COVID-19 vaccine: trust, doubt, and hope for a future beyond the pandemic in Germany. PLoS One. 2022;17(4):e0266659. doi:10.1371/journal.pone.0266659 35390085 PMC8989326

[31] Kuhn SAK, Lieb R, Freeman D, Andreou C, Zander-Schellenberg T. Coronavirus conspiracy beliefs in the German-speaking general population: endorsement rates and links to reasoning biases and paranoia. Psychol Med. 2021;52(16):1-15. 33722315 10.1017/S0033291721001124 PMC8027560

[32] Klüver H, Hartmann F, Humphreys M, Geissler F, Giesecke J. Incentives can spur COVID-19 vaccination uptake. Proc Natl Acad Sci U S A. 2021;118(36):e2109543118. doi:10.1073/pnas.2109543118 34413212 PMC8433545

[33] Adalja AA, Toner E, Inglesby TV. Priorities for the US health community responding to COVID-19. JAMA. 2020;323(14):1343-1344. doi:10.1001/jama.2020.3413 32125355

[34] de Vries DH, Kinsman J, Cremers AL, . Public health preparedness and response synergies between institutional authorities and the community: a qualitative case study of emerging tick-borne diseases in Spain and the Netherlands. BMC Public Health. 2021;21(1):1882. doi:10.1186/s12889-021-11925-z 34663298 PMC8524986

