# Supplementary material for: Financial Incentives for COVID-19 Vaccination: A Cluster Randomized Clinical Trial
Source: JAMA Netw Open. 2025 Feb 7;8(2):e2458542. doi: 10.1001/jamanetworkopen.2024.58542 (PMC11806395; doi:10.1001/jamanetworkopen.2024.58542)
Supplement: Supplement 1. — Trial Protocol [file jamanetwopen-e2458542-s001.pdf]

[English translation of the German original]

Ethics Committee of the
Zeppelin University

29.10.2021

Application for review and approval by the Ethics Committee for the field experiment: "The Effect of Monetary Incentives on COVID-19 Vaccination Uptake: A Randomized Control Trial"

Dear Ethics Committee,

With this letter, we are applying for an assessment and approval by the Ethics Committee in accordance with the university guidelines for the above-mentioned research project. In addition to the completed checklist university checklist, please find the trial protocol attached.

Best regards

Florian Keppeler, on behalf of the research project team

<u>Attachments:</u>
Study description
Checklist
Trial protocol

STUDY DESCRIPTION

Research project team:

- Florian Keppeler, Dr., Postdoctoral Researcher, Chair of Public Management & Public Policy, Zeppelin University
- Sebastian Jilke, Ph.D., Associate Professor, McCourt School of Public Policy, Georgetown University, Washington DC
- Dominik Vogel, Prof. Dr., Junior Professor of Business Administration, esp. Public Management, University of Hamburg
- John Ternovski, Ph.D. Cand., Yale University and Research Associate, McCourt School of Public Policy, Georgetown University, Washington DC

Planned data collection period: 01.11.2021 – ca. 30.04.2022

Project description:

Project objective and research question:

The aim of the project is to analyze the extent to which monetary incentives (offered in an official letter) lead to people being vaccinated against COVID-19.

Vaccination campaigns against COVID-19 aim to protect the community as a whole in addition to individual protection by building herd immunity. As vaccination is an effective means of combating the spread of the viral disease, promoting vaccination rates is an important element of public health policy. However, in many regions, not least in Baden-Württemberg, the vaccination rate is too low (as of 10.10.2021. approx. 60%).[1] Herd immunity as a political goal is nowhere near being achieved in many areas, not only in Germany. Recently, it has been suggested in the political debate and by various scientific disciplines that incentives should be created to improve vaccination rates. This is considered a milder means compared to other political measures such as mandatory vaccination. The present study aims to make an empirical contribution by conducting a field experiment (N = 41,881 adults in 10,035 address clusters).

In particular, the research design tests the effects of a monetary incentive as part of a municipal vaccination campaign in cooperation with the city of Ravensburg. Against the background of the current pandemic and the question of official vaccination campaigns

---

[1] https://sozialministerium.baden-wuerttemberg.de/fileadmin/redaktion/m-sm/intern/downloads/Downloads_Gesundheitsschutz/Corona_Gesamtzahl-Impfungen-Landkreise-BW.pdf, accessed on 12.10.2021.

or stricter political measures, the project has a very high scientific and practical relevance (Cheng et al. 2020; Lunn et al. 2020; Keppeler et al. 2021; Loomba et al. 2021; Patel 2021).

Specifically, the effect of two official information letters sent by the mayor to the adult population of the city of Ravensburg is analyzed as part of a field experiment (randomized control trial). The following treatment and control groups are analyzed in comparison:

- Control group A: Official letter emphasizing the benefits of vaccination against COVID-19 and advertising weekend vaccination appointments at the town hall.

- Treatment group B: Official letter emphasizing the benefits of vaccination against COVID-19 and advertising weekend vaccination appointments at the town hall. In addition, a 20-euro shopping voucher is offered if the person contacted is unvaccinated and attends the vaccination. Furthermore, the person contacted will be offered an additional voucher of 20 euros if more than 900 people from the city area are vaccinated on the municipal vaccination dates. This 20-euro incentive also applies to people who have already been vaccinated and who motivate another person to get vaccinated.

With this study design, the causal effects of monetary incentives and possible indirect network effects of the incentives within the address clusters can be analyzed. The dependent variable is the actual vaccination at one of the municipal vaccination appointments. For this purpose, the name and address data of those people who actually get vaccinated are collected by the city of Ravensburg. In addition, the letters contain individual online links to the information website, which make it possible to measure the information behavior for the respective treatment/control group.

Background, research gap and literature:

Current and future COVID-19 vaccination efforts are initially aimed at protecting the vaccinated person from infection and severe disease progression. Monetary vaccination incentives show promising effects in initial, current field and survey experiments (Campos-Mercade Pol et al. 2021; Klüver et al. 2021), are also discussed critically (Largent and Miller 2021).

Against this background, the present empirical study can make a relevant contribution to research. For example, the indirect network effects (Miguel and Kremer 2004), e.g. within address clusters, have not yet been investigated. Network effects occur when people in the treatment group who receive a letter with a monetary incentive convince other people in the same address cluster to also be vaccinated. On the basis of comparable research in the field of general vaccination research (Halloran 2012;

Benjamin-Chung et al. 2018), as well as in other policy areas (Miguel and Kremer 2004; Angelucci and De Giorgi 2009), it can be assumed that such network effects could be pronounced (Benjamin-Chung et al. 2018). Due to the staggered incentive in the treatment group, network effects are further strengthened and can in all likelihood have a greater impact than the direct effects of the treatment. In the course of the COVID-19 discussion, such staggered incentives were proposed by prominent economists,[2] but not yet empirically tested.

Current scientific findings on the effects of monetary incentives on vaccination rates in the German context are based on survey experiments (Klüver et al. 2021). However, there is a lack of field-experimental studies that analyze specific measures with high external validity (Brewer et al. 2017; Patel 2021). The American Psychological Association emphasizes the importance of field studies or field experiments during COVID-19 vaccination efforts: „Interventions need to be tested in a real-world context.“[3] Nobel Prize winner Esther Duflo's Poverty Action Lab is also currently calling for action: “[…] research on how to boost adoption of preventive measures could help us understand how to increase immunization uptake.“[4]

Against the background of this urgent research gap, research is being conducted in this field experiment in collaboration with the city of Ravensburg as part of an already planned official campaign.

The results are intended to contribute to public management and public health research in order to better understand the extent to which monetary incentives can actually help to increase the vaccination rate. The analysis also promises to provide concrete, practicable options for action for political decision-makers in practice.

Research design:

Through the planned field experiment (Harrison and List 2004; Levitt and List 2009; Eden 2017; Banerjee 2020), the findings can make a significant contribution to scientific knowledge in the context of the study. The design promises large-scale empirical findings. In addition, the use of a real setting enables high external validity compared to other research approaches (Eden 2017).

---

[2] https://www.brookings.edu/opinions/want-herd-immunity-pay-people-to-take-the-vaccine/, accessed on 19.10.2021
[3] https://www.apa.org/monitor/2021/03/covid-19-vaccines, accessed on 9.3.2021
[4] https://www.povertyactionlab.org/blog/2-18-21/closing-covid-trust-deficit, accessed on 9.3.2021

In this project, a field experiment is being carried out with the city of Ravensburg. The Lord Mayor is already planning to write to households in the city of Ravensburg in the first week of November and ask them to get vaccinated on one of the dates organized by the city. This official vaccination campaign will be used as an opportunity for accompanying scientific research for the purpose of an analysis in the form of a "between-group design" with two groups (visualized in the figure below).

Figure 1: Visualization of the field experiment procedure

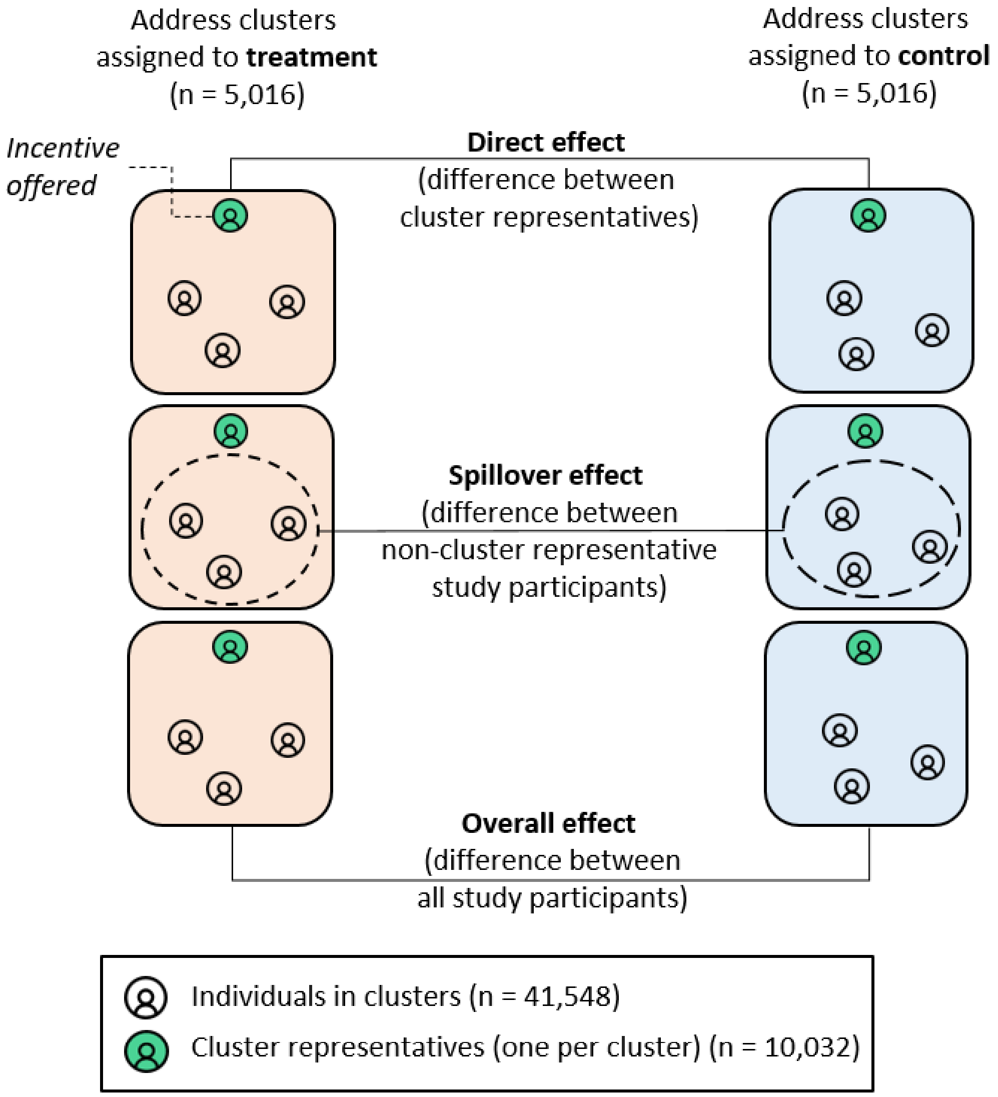

<u>Randomization:</u> A two-stage randomization procedure is used. To begin with, the address clusters in the Ravensburg urban area are randomly divided into two groups of equal size (A, B) on the basis of register data from the residents' registration office. An address cluster consists of all persons registered at the same address. People within each address cluster are then randomly selected and contacted. This design makes it possible to examine not only the direct effects on the person contacted, but also possible network effects on other people living at the same address.

<u>Measurement:</u> The city of Ravensburg will measure which person actually gets vaccinated at one of the vaccination appointments organized by the city (collection of name and address data).

It will also measure how many people visit the website of the city of Ravensburg shown on the respective letter to obtain information on vaccination and vaccination dates (access to links A, B). The city will also record how many people from which group have contacted us by telephone.

Whether person X was motivated by another person (M) to get vaccinated is determined by the fact that person X, who gets vaccinated, carries the letter addressed to person M with them when they get vaccinated or provides corresponding information. This information is also collected by the city employees.

<u>Data protection and data security:</u> Personal data (e.g. address data or IP addresses) remain with the city of Ravensburg and the German part of the research team. The following data is transmitted to the research team:

- Name and address of the person to be contacted so that the research team can randomly assign the persons to the groups. This list with the assignment of people to the experimental groups can then be merged by the city of Ravensburg with the list of people who were actually vaccinated during the campaign. This enables the dependent variable to be measured.

- Information on the undeliverable letters per group

- Overview of the link accesses (links in Figure 1) and the number of clicks on the respective website outlined above.

The processing of data for scientific purposes is carried out in accordance with the German Federal Data Protection Act and the EU General Data Protection Regulation. The City of Ravensburg has also completed its own data protection review for the vaccination campaign.

Selection of participants: The city of Ravensburg will write to the adult citizens in the city area on a person-by-person basis. Allocation to the groups will be randomized as described above.

Treatment: As described above, in addition to the vaccination, randomly selected members of the treatment group will be offered a shopping voucher worth EUR 20. In addition, if the number of people vaccinated exceeds 900 during the vaccination campaign appointments, a further shopping voucher worth 20 euros will be offered. This incentive is also available to people who have already been vaccinated. Participants in the treatment group will be informed in the letter that they belong to a randomly selected group of people who will be offered such an incentive. The city of Ravensburg does not have the financial means to offer such an incentive to everyone, which is why the network effect design is effective.

What happens to the participants? The adult citizens of the city of Ravensburg receive a letter with a link to a website of the city of Ravensburg. The people who are randomly assigned to the treatment group are informed about a monetary incentive for the vaccination, which is staggered (20 euros immediately after the vaccination, 20 euros if a certain number of vaccinations are achieved as part of the municipal vaccination campaign).

UNIVERSITY SELF-ASSESSMENT CHECKLIST

	Yes	No	Don't know
1. Does the study avoid obtaining an informed consent of participants?	☒	☐	☐
2. Will the study collect and use data that is not anonymized (e.g., name, video or audio recordings from participants)[1]	☒	☐	☐
3. Will the study involve vulnerable individuals or groups who cannot themselves provide informed consent (e.g. children, people with learning disabilities, clinical populations)?	☐	☒	☐
4. Does the study involve participants that are uniformed about their participation in the study?	☒	☐	☐
5. Are the participants asked to provide intimate, sensitive or distressing information (e.g. about illegal or deviant behavior, sexual orientation, traumatic experiences)?	☐	☒	☐
6. Could the study negatively affect participant's physical integrity (e.g., taking medications or alcohol, taking blood, physical strain through physical exertion)?	☐	☒	☐
7. Could the study negatively affect participant's psychological integrity (e.g., triggering mental stress, severe emotions, pain or other negative effects that go beyond an everyday level)?	☐	☒	☐
8. Is there a substantial dependency relationship between the participants and any of the involved researchers?	☐	☒	☐
9. Will the participants be intentionally deceived or mislead by the researcher (e.g., by manipulated performance feedback, by providing false information about the objectives of the investigation)?	☐	☒	☐
10. Does the study involve coercive financial or non-financial incentives (i.e., incentives that can threaten the voluntary choice to participate in the study)?	☐	X	☐
11. Is the purpose of the study to significantly influence participant's life (e.g., influencing people's voting behavior, people's job search behavior or outcomes)?	X	☐	☐
12. Does any member of the research team have any association that poses or could be perceived as posing a conflict of interest in connection with the results of the study?	☐	X	☐

A) No informed consent (checklist #1 and #4)

The letter to the adult citizens of the city of Ravensburg states: *"The vaccination campaign is scientifically monitored"*. Furthermore, the treatment group is informed in the letter that they were selected "randomly" according to the randomization. However, the population is not informed in detail that they are taking part in a field experiment. The advantage of this approach is that the external validity of the results is higher than in a laboratory experiment, for example, as it is a real situation with a real vaccination schedule (Harrison and List 2004). Providing information about the field experiment in advance would make the field experiment design impossible or distort the results. Since all letters contain information about the COVID-19 vaccination or the vaccination dates, the interest in knowledge clearly outweighs the interest in information in advance from a research perspective. A detailed explanation of accompanying research could lead to already hesitant/vaccine-skeptical people being further deterred. The Hawthorne effect is particularly worrying in this context, because it is acceptable that a reference to accompanying research could already influence vaccination behavior if a feeling of scientific observation arises. In the popular debate, it is not uncommon for people to argue against vaccination on the grounds that they are being used as test subjects. It is precisely this feeling that must be avoided in the context of vaccination.

For this research project, we are guided by medical standards and the procedure described in the relevant literature (Benjamin-Chung et al. 2018). Recent publications also show that this approach is internationally recognized and practiced in the scientific community (Campos-Mercade Pol et al. 2021; Chang et al. 2021).

Furthermore, the principle of informed consent is usually sufficiently taken into account if the research project is carried out by public institutions (in this case the city of Ravensburg) and their consent has been obtained. The project is intended to examine or test a public benefit or service program (here: the effects of the vaccination campaign).[5] *The population contacted by the city will not be deceived and will receive all information on opportunities and risks. In particular, before the vaccination, the usual information is provided by the medical staff and a written consent to the vaccination is given. The city of Ravensburg will report publicly on the study once it has been completed and the research team has evaluated the results.*

[5] Poverty Action Lab https://www.povertyactionlab.org/sites/default/files/research-resources/rr_irb_annotated-informed-consent-checklist_0.pdf, p. 4 f., accessed on 28.10.2021.

The field experiment will not expose the adult citizens of the city of Ravensburg to any additional burden or stress; rather, they will receive the necessary information on vaccination or vaccination dates and may have less research effort than third parties dealing with the vaccination decision. They would be exposed to these letters even if the experiment had not been carried out, as the city of Ravensburg is planning to send such a letter anyway. The experimental character results solely from the randomization of a monetary incentive, which does not result in any disadvantages for the participants. Randomizing monetary incentives is a comparatively fairer option than awarding such incentives on a "first come, first served" basis, for example, which would favor people with more resources. The letters can also be ignored without consequences and the website links do not have to be visited. There is therefore no or only a minor impairment or restriction. It is not a form of deception, as there is a real possibility for all groups to attend a vaccination appointment. The population will be informed by the city of Ravensburg after the study that this is a scientifically supported procedure and that there were addressee groups with different letters.

B) Personal data (Checklist #2)
For the field experiment, personal data is used for the purposes of contacting and measuring the effects, which the city of Ravensburg collects after its own data protection review and may make available for research purposes. For research purposes, the data will be pseudonymized or anonymized as far and as soon as possible, especially for the evaluation after the cover letter.

C) Influence on individual life (Checklist #11)
A vaccination can be described as having a significant influence on individual life. From a scientific point of view, in the vast majority of cases this is a very positive influence on individual and public welfare. Incidentally, the people taking part agree to the vaccination of their own free will and receive the medical information required by law before being vaccinated.

In this context, it should be emphasized that this field experiment is about the vaccination itself. The city of Ravensburg would carry out a corresponding information campaign to increase the vaccination rate with incentives anyway, even if there was no cooperation within the framework of a field experiment. In addition, the official information website linked in the letter presents the potential risks of vaccination according to the official information from the Federal Ministry of Health, in comparison to the risks of a COVID-19 infection.

As confirmed in relevant published articles (Brewer et al. 2017; Lunn et al. 2020), from a research perspective, the interest in measures to increase the vaccination rate

outweighs the conceivable adverse effects on individuals due to incentives or information letters (Patel 2021). As stated at the beginning, the American Psychological Association emphasizes the importance of field studies and field experiments during COVID-19 vaccination efforts: "Interventions need to be tested in a real-world context." [6]. There is also considerable public interest in the knowledge gained from this study.

Bibliography

Angelucci, Manuela, and Giacomo De Giorgi. 2009. Indirect Effects of an Aid Program: How Do Cash Transfers Affect Ineligibles' Consumption? *American Economic Review* 99: 486–508.

Banerjee, Abhijit Vinayak. 2020. Field experiments and the practice of economics. *American Economic Review* 110: 1937–1951.

Benjamin-Chung, Jade et al. 2018. Spillover Effects in Epidemiology: Parameters, Study Designs and Methodological Considerations. *International Journal of Epidemiology* 47: 332–347.

Brewer, Noel T., Gretchen B. Chapman, Alexander J. Rothman, Julie Leask, and Allison Kempe. 2017. Increasing Vaccination: Putting Psychological Science Into Action. *Psychological Science in the Public Interest* 18: 149–207.

Campos-Mercade Pol et al. 2021. Monetary Incentives Increase Covid-19 Vaccinations. *Science* 0: eabm0475.

Chang, Tom, Mireille Jacobson, Manisha Shah, Rajiv Pramanik, and Samir B. Shah. 2021. *Financial Incentives and Other Nudges Do Not Increase COVID-19 Vaccinations among the Vaccine Hesitant*. Cambridge, Mass: National Bureau of Economic Research.

Cheng, Cindy, Joan Barceló, Allison Spencer Hartnett, Robert Kubinec, and Luca Messerschmidt. 2020. COVID-19 Government Response Event Dataset (CoronaNet v.1.0). *Nature Human Behaviour* 4: 756–768.

Eden, Dov. 2017. Field Experiments in Organizations. *Annual Review of Organizational Psychology and Organizational Behavior* 4: 91–122.

Halloran, M. Elizabeth. 2012. The Minicommunity Design to Assess Indirect Effects of Vaccination. *Epidemiologic Methods* 1.

Harrison, Glenn W, and John A List. 2004. Field Experiments. *Journal of Economic Literature* 42: 1009–1055.

Keppeler, Florian, Martin Sievert, and Sebastian Jilke. 2021. How Local Government Vaccination Campaigns Can Increase Willingness to Get Vaccinated Against

[6] https://www.apa.org/monitor/2021/03/covid-19-vaccines, accessed on 9.3.2021

Covid-19: A Field Experiment on Psychological Ownership. *SSRN Electronic Journal* https://doi.org/10.2139/ssrn.3905470.

Klüver, Heike, Felix Hartmann, Macartan Humphreys, Ferdinand Geissler, and Johannes Giesecke. 2021. Incentives Can Spur Covid-19 Vaccination Uptake. *Proceedings of the National Academy of Sciences* 118.

Largent, Emily A., and Franklin G. Miller. 2021. Problems With Paying People to Be Vaccinated Against COVID-19. *JAMA* 325: 534.

Levitt, Steven D., and John A. List. 2009. Field experiments in economics: The past, the present, and the future. *European Economic Review* 53: 1–18.

Loomba, Sahil, Alexandre de Figueiredo, Simon J. Piatek, Kristen de Graaf, and Heidi J. Larson. 2021. Measuring the impact of COVID-19 vaccine misinformation on vaccination intent in the UK and USA. *Nature Human Behaviour* 5.

Lunn, Peter D. et al. 2020. Using Behavioral Science to help fight the Coronavirus. *Journal of Behavioral Public Administration* 3: 1–15.

Miguel, Edward, and Michael Kremer. 2004. Worms: Identifying Impacts on Education and Health in the Presence of Treatment Externalities. *Econometrica* 72: 159–217.

Patel, Mitesh S. 2021. Test behavioural nudges to boost COVID immunization. *Nature* 590: 185–185.

TRIAL PROTOCOL
Draft, to be pre-registered on Open Science Framework

Hypotheses
H1-1 (direct or "total" effect #1): Offering a set of monetary incentives increases the number of unique website visits (on a municipal webpage with information on COVID-19 vaccination) among treated subjects in treatment clusters compared to equivalent subjects in control clusters (i.e., in each control cluster, the person who would have received the treatment letter had they been assigned to a treatment cluster). H1-2 (direct or "total" effect #2): Offering a set of monetary incentives increases COVID-19 vaccinations (tracked at seven city vaccinations events) among treated subjects in treatment clusters compared to equivalent subjects in control clusters (i.e., in each control cluster, the person who would have received the treatment letter had they been assigned to a treatment cluster). H2 (spillover effect): Offering a set of monetary incentives increases COVID-19 vaccinations among untreated individuals in treatment clusters compared to untreated subjects in control clusters (minus the individual who would have received a treatment letter in the control cluster if they were assigned to a treatment cluster). H3-1 (overall effect #1): Offering a set of monetary incentives increases unique website visits among treated and untreated individuals in treatment clusters compared to untreated subjects in control clusters. H3-2 (overall effect #2): Offering a set of monetary incentives increases COVID-19 vaccinations among treated and untreated individuals in treatment

Design Plan
Study type
Experiment

Blinding
No blinding is involved in this study.

Study design
The design is a blocked, two-stage (individual and household) randomized controlled field experiment.

Randomization
The study will be conducted in a medium-sized city in Germany. Treatment assignment follows a two-stage randomization process. In the first stage, all housing addresses within the city will be randomized into two groups (i.e., intervention group and control group), blocked by household size. In the second stage, one resident of each address in both groups will be randomly selected (a “cluster representative”). The cluster

representatives in the intervention group only will be sent the intervention letter. All remaining residents will be sent the control letter. The randomization will be conducted in R (version 4.1.1) using the function “sample” with the seed set to 2021.

Sampling Plan
Existing Data
Registration prior to creation of data

Data collection procedures
The study will be conducted in the city of Ravensburg, Germany. All residents of the city (age 18 and older) will be subject to the trial (as per official residency records). The only notable exception is that the study excludes the inmates of a local prison (n=257) and the second largest cluster of the city (n=75). Treatment assignment follows a two-stage randomization process. In the first stage, all housing addresses within the city will be randomized into two groups (i.e., intervention group and control group), blocked by household size. In the second stage, one resident of each address in both groups will be randomly selected (a “cluster representative”). The cluster representatives in the intervention group only will be sent the intervention letter. All remaining residents will be sent the control letter. The treatment group will receive a letter from the mayor of the city inviting them to get a COVID-19 vaccination at one of seven public vaccination events. The letter offers two financial incentives. If the recipient is not already vaccinated and gets vaccinated at the vaccination events, they will receive 20 Euros (in the form of a shopping voucher). Additionally, another 20 Euro incentive is offered if more than 900 city residents get vaccinated at one of the seven vaccination events. If the recipient is already vaccinated, they can still get the second incentive if they give the treatment letter to someone else and that individual brings it to one of the seven vaccination events, gets vaccinated, and more than 900 city residents get vaccinated during the one of the seven vaccination events. The control group will get the same letter as the treatment group but without the offer of monetary incentives.

Sample size
The sample consists of all residents of the city (n = 41,549) clustered into 10,033 addresses. The only notable exception is that the study excludes the inmates of a local prison (n=257) and the second largest cluster of the city (n = 75).

Sample size rationale
The sample size is determined by the population of the city. Based on simulation-based power calculations, we are able to detect a minimal direct or “total” effect of 2.6 percentage points, a spillover effect of 1.5 percentage points, and an overall effect of 1.3 percentage points with approximately 80% statistical power (alpha = 0.05). This

calculation is based on the assumption that 20% of the population of the city i not eligible for a primary vaccine or booster and that eligibility is uniformly distributed across individuals. We project that up to 20% of the control group will get vaccinated at these events. The code for the power calculation is attached under "data collection procedures".

Stopping rule
The effect of the treatment will be assessed by measuring how many people get vaccinated at the seven public vaccination events on 2021-11-13, 2021-11-19, 2021-11-20, 2021-11-26, 2021-11-27, 2021-12-10, and 2021-12-11.

Variables
Manipulated variables
The treatment group will receive a letter from the mayor of the city inviting them to get a COVID-19 vaccination at one of seven public vaccination events. The letter offers two financial incentives. If the recipient is not already vaccinated and gets vaccinated at the vaccination events, they will receive 20 Euros (in the form of a shopping voucher). Additionally, another 20 Euro incentive is offered if more than 900 city residents get vaccinated at one of the seven vaccination events. If the recipient is already vaccinated, they can still get the second incentive if they give the treatment letter to someone else and that individual brings it to one of the seven vaccination events, gets vaccinated, and more than 900 city residents get vaccinated during the one of the seven vaccination events. The control group will get the same letter as the treatment group but without the offer of monetary incentives.

Measured variables
Primary measures: Information uptake is measured by whether a participant visited the informational website mentioned in the letter (unique website visits). The website is prominently displayed in both treatment and control letters. Each URL and QR-code is unique to the individual recipient. Vaccination uptake is measured via an on-site record of administered vaccinations during the seven public vaccination events. The records will distinguish between primary vaccines (i.e., first and second doses) and boosters. Covariate measures: These data will be collected from municipal administrative records and will be used as covariates to increase precision - Age - Sex - Indicator for whether the individual is a non-German national Additional measures: To determine an individual’s close social network, we use address data from municipal administrative records to identify shared addresses and generate unique clusters. As an alternative specification, we use shared last name and shared address to generate a secondary cluster id. The treatment letters given to other residents by fully-vaccinated treatment letter recipients will be collected at the public vaccination events to track which

subjects were referred to get vaccinated by the treatment-letter recipient. We will also collect information (self-reports) whether patients are part of a vulnerable high-risk group which the German federal government prioritizes in the vaccine distribution.

Analysis Plan

Statistical models

We will use OLS regression with a treatment indicator, block fixed effects, and control covariates (consisting of a binary indicator for female, an indicator for non-German nationality, age, and age squared) in all subsequent analyses unless indicated otherwise. To ensure robustness, a secondary analysis will regress outcomes against the treatment indicator without controls. H1-1 (direct or "total" effect #1): Information uptake regressed against treatment group for only cluster representatives, with robust standard errors. H1-2 (direct or "total" effect #2): Vaccination uptake regressed against treatment group for only cluster representatives, with robust standard errors. H2 (spillover effect): Vaccination uptake regressed against treatment group for all individuals who are not cluster-representatives, with robust clustered standard errors. H3-1 (overall effect #1): Information uptake regressed against treatment group for all individuals, with robust clustered standard errors. H3-2 (overall effect #2): Vaccination uptake regressed against treatment group for all individuals, with robust clustered standard errors.

Inference criteria

We will use p-values (threshold = 0.05) associated with the treatment indicator coefficient in our regressions.

Data exclusion

Aside from the exclusion of the two largest clusters, since we use block fixed effects, the one subject in a single-person household that was randomly determined to be in a block size of one will be dropped from analysis.

Exploratory analysis

For the analysis, all residents living at the same address will be treated as members of the same social network. We will test an alternative operationalization of a social network by considering all residents with the same last name who reside in the same address cluster as members of the same social network. We will also explore whether the hypothesized effects differ by German vs. non-German nationals. To better map out social influence, we will also compare which letters were given to individuals living in the same address as the treatment-letter recipient, which were given to individuals in the same address with the same last name, and which were given to individuals at a different address. Additionally, we will conduct analysis restricted to 1) first doses

(excluding second doses and booster shots), 2) first and second doses (excluding booster shots), and 3) boosters (excluding first and second doses). To explore whether the treatment effects in H1-2, H2, and H3-2 are driven by an increase in booster shots, we will perform the analyses as described in H1-2, H2, and H3-2 but using a multinomial logistic regression with no record of vaccination equal to zero, primary vaccination equal to 1, and booster vaccination equal to 2. A Wald test will be used to determine if the treatment coefficient in terms of primary vaccination is statistically significantly different from that of boosters.

Other

Other

This preregistration is an update to a previous one. We published it prior to collecting any data. The update was necessary to add exploratory analyses to capture regulatory interventions by government bodies that might occur during the study period and could affect the effects of the treatment as well as to capture decisions made by the involved city. We, therefore, added the last three paragraphs under "exploratory analysis", and specified that people getting vaccinated multiple times will only be counted once (see "Data exclusion")
