# Supplementary material for: Financial Incentives for COVID-19 Vaccination: A Cluster Randomized Clinical Trial
Source: JAMA Netw Open. 2025 Feb 7;8(2):e2458542. doi: 10.1001/jamanetworkopen.2024.58542 (PMC11806395; doi:10.1001/jamanetworkopen.2024.58542)
Supplement: Supplement 2. — eAppendix 1. Treatment and Control Letters eAppendix 2. Data Collection eAppendix 3. Timeline of Experiment and Relevant Policy Changes eAppendix 4. Types of Treatment Effects eAppendix 5. Investigating Postponement Spillovers eAppendix 6. Robustness Checks eAppendix 7. Statistical Power and Sensitivity Analysis eAppendix 8. Data and Code Availability eAppendix 9. Ethics eAppendix 10. Pre-registered Analysis Plan eAppendix 11. Additional Analyses eAppendix 12. The Development and Prevalence of Evidence-Based Policy-Making eReferences [file jamanetwopen-e2458542-s002.pdf]

Supplemental Online Content

Ternovski J, Jilke S, Keppeler F, Vogel D. Financial incentives for COVID-19 vaccination: a cluster randomized clinical trial. *JAMA Netw Open*. 2025;8(2):e2458542. doi:10.1001/jamanetworkopen.2024.58542

eAppendix 1. Treatment and Control Letters
eAppendix 2. Data Collection
eAppendix 3. Timeline of Experiment and Relevant Policy Changes
eAppendix 4. Types of Treatment Effects
eAppendix 5. Investigating Postponement Spillovers
eAppendix 6. Robustness Checks
eAppendix 7. Statistical Power and Sensitivity Analysis
eAppendix 8. Data and Code Availability
eAppendix 9. Ethics
eAppendix 10. Pre-registered Analysis Plan
eAppendix 11. Additional Analyses
eAppendix 12. The Development and Prevalence of Evidence-Based Policy-Making
eReferences

This supplemental material has been provided by the authors to give readers additional information about their work.

© 2025 Ternovski J et al. *JAMA Network Open*

eAppendix 1. Treatment and Control Letters

1.1 Control Letter (English Translation)

[Personalized Address]

Get your COVID-19 vaccination now!

Hello [ACADEMIC TITLE] [NAME],

We are contacting you today for your coronavirus vaccination.

Are you not yet fully vaccinated? Then please go for vaccination on one of the following dates:

- **13.11.:** Saturday, 9 a.m.-2 p.m.
- **19-20.11.:** Friday, 3-9 p.m. or Saturday, 9 a.m.-2 p.m.
- **26-27.11.:** Friday, 3-9 p.m. or Saturday, 9 a.m.-2 p.m.
- **10-11.12.:** Friday, 3-9 p.m. or Saturday, 9 a.m.-2 p.m.

Place: City Hall, Marienplatz 26, 88212 Ravensburg **(without appointment)**

Are you already fully vaccinated? Then motivate your environment from Ravensburg now to a vaccination on one of the mentioned dates.

Available are vaccines from BioNTech/Pfizer, Moderna and Johnson & Johnson. Please bring, if available, vaccination certificate, identity card and insurance card of the health insurance. For more information, please visit the following website: [INDIVIDUAL LINK].

Kind regards

[Signature, name, and picture of the local Mayor, chairman of the local association of the medical profession, leading physician of the local public clinic]

[Text on the right side in the column: Contact details of the sender, i.e. the Mayor, date, reference number, phone number and email address for questions, text: "all info via the link or directly via the QR code", translation note for the languages "English, Spanish, Russian, Arabic, Turkish", additional note: "This vaccination campaign is scientifically accompanied. It is supported by:", logos of sponsors]

1.2 Treatment Letter (English Translation)

[Personalized Address]

Get your COVID-19 vaccination now, receive a 20 Euro voucher and secure the chance to win another 20 Euro!

Hello [ACADEMIC TITLE] [NAME],

Congratulations! The city of Ravensburg is giving away shopping vouchers. You are one of 5,000 **randomly selected** people from Ravensburg who can receive these limited vouchers.

Then please go for vaccination on one of the following dates.

© 2025 Ternovski J et al. *JAMA Network Open*

Are you not yet fully vaccinated? Then please go for vaccination on one of the following dates. **You will receive your 20 Euros voucher for this** by mail after the vaccination.

- **13.11.:** Saturday, 9 a.m.-2 p.m.
- **19-20.11.:** Friday, 3-9 p.m. or Saturday, 9 a.m.-2 p.m.
- **26-27.11.:** Friday, 3-9 p.m. or Saturday, 9 a.m.-2 p.m.
- **10-11.12.:** Friday, 3-9 p.m. or Saturday, 9 a.m.-2 p.m.

Place: City Hall, Marienplatz 26, 88212 Ravensburg **(without appointment)**

After your vaccination on one of these dates, you also have a **chance to win**: if more than 900 people from Ravensburg get vaccinated on these dates, you will receive **your 2nd voucher worth 20 Euros** by mail.

Are you already fully vaccinated? Then motivate your environment from Ravensburg now to a vaccination on one of the mentioned dates.If this person shows your letter at one of the mentioned dates, you secure your **chance to win**: If more than 900 people from Ravensburg get vaccinated, you will receive **your 20 Euros voucher** by mail.

Available are vaccines from BioNTech/Pfizer, Moderna and Johnson & Johnson. Please bring, if available, vaccination certificate, identity card and insurance card of the health insurance. For more information, please visit the following website: [INDIVIDUAL LINK].

Kind regards

[The rest of the text is identical to the control letter.]

eAppendix 2. Data Collection

This section describes how our outcome variables were collected.

The variable "**information uptake"** describes whether a letter recipient accessed the personalized link or QR code that was prominently displayed on both the control and treatment letters.[1] The links led to a city website, which informed residents about COVID-19 vaccines and the upcoming city-organized vaccination events, and encouraged them to get vaccinated. As noted in the letter, the website could be viewed in German and–as an additional service for migrant residents–in other languages commonly spoken in the region (English, Spanish, Russian, Arabic, and Turkish).

The link addresses incorporated a randomly-generated five-character code (e.g., 123AB) to identify individual click-behavior (i.e., which recipient visited the website and which did not). For ease of access and to enable us to measure link clicks in accordance with EU GDPR regulations, the links shortening web application "rebrand.ly" and its secure API were used to create the personalized links (i.e., link.com/123AB) and generate the "unique clicks" variable.

The variable **"vaccination uptake"** describes whether a resident was vaccinated at one of the advertised public vaccination events. This data was collected as follows.

The persons who arrived at the vaccination site to be vaccinated were asked to wait in line outside of the site in order to reduce risk of infection. City security staff was present on-site. Initially, security staff invited the first person in line into the vaccination site and progressed through the line in this manner until the vaccine doses were depleted for the day. Once vaccine doses were depleted the remaining individuals in the line were asked to return on a different day. Since this approach risked disincentivizing vaccination, a waitlist system was introduced starting with the fourth announced event (11/26/2021). Under the waitlist system, the city distributed numbered paper cards, with the total number of cards determined by the number available vaccine doses. The numbers on the cards were associated with certain vaccination appointment times, so that waitlisted people could come back to the vaccination site instead of waiting in long lines outside of the site. This had the benefit of reducing wait time and the risk of infection.

The process inside the vaccination site building was the same with and without the waitlist system. Once inside, the individual to be vaccinated proceeded to the front desk where city officials collected data. The individual was asked to present an ID or comparable document. Officials at the front desk would copy this information into a spreadsheet. Then, the individual was directed to a different table (in the same physical space), where medical staff would provide a medical consultation as required by law. In other words, the medical staff would provide information about the type of vaccination that would be administered and answer individual health questions. If the individual decided to opt out after the medical consultation, the data collection team was informed and made a note of this in the collected data. There were four opt-outs; all four were coded as unvaccinated in all analyses in the study. Finally, the vaccination was administered and individuals were supervised afterward in a monitoring room in case of adverse (i.e., allergic) reactions. The entirety of this data collection process was overseen by one of the researchers at all events. The researchers then matched the collected data with the list of letter recipients.

[1]We tracked both whether or not a personalized link/QR code was accessed *and* how often it was accessed.

© 2025 Ternovski J et al. *JAMA Network Open*

eAppendix 3. Timeline of Experiment and Relevant Policy Changes

This population-level field experiment was conducted in the city of Ravensburg, Germany in the fall of 2021. Details of the policy context at the time the experiment was conducted can be found in the SM (Sections 1.1 and 1.10) of a related paper, Jilke et al. (2023). We provide an extended version of the timeline in Jilke et al. (2023, SM, p. 4) here:

10/29/2021	The Zeppelin University ethics committee issues its approval of this study.
10/29/2021	All Ravensburg residents are randomized to be sent either a treatment letter (with information about the city's upcoming free, public vaccination events AND individual-level and community-level financial incentives) or a control letter (identical to the treatment letter but with no individual-level or community-level financial incentives).
11/05/2021	Treatment and control letters are sent out.
11/05/2021	The federal health ministry and the state ministries in Germany recommend booster vaccination for all persons aged 18 and over.
11/10/2021	Federal and state authorities decide to offer only the Moderna vaccine to persons aged 30 and older, allotting the Pfizer/BioNTech vaccine for younger persons. (There were very few doses of the Pfizer/BioNTech vaccine available in Germany, at this time.)
11/13/2021	The first of the seven announced vaccination events takes place.
11/15/2021	Due to high demand and long lines at the first vaccination event, Ravensburg organizes 11 additional vaccination events. There is no additional mailed communication about these events, however the original letters included a QR code to a government website and a hotline number. From this point on, the website and hotline included information about the additional, unannounced events.
11/17/2021	The regional health ministry of Baden-Wuerttemberg introduces the "2G rule," restricting access to most shops and restaurants to people who either 1) received the first and second dose or 2) recovered from COVID-19.
11/18/2021	The Standing Committee on Vaccination, an independent expert committee in Germany, recommends booster vaccination for all persons older than 18, six months after the second dose.
11/19/2021	The second of the seven announced vaccination events takes place.
11/20/2021	The third of the seven announced vaccination events takes place.
11/22/2021	The federal health ministry restricts the delivery of the BioNtech/Pfizer vaccine to reserve it for minors under the age of 18 and offers Moderna vaccines as an alternative.
11/26/2021	The fourth of the seven announced vaccination events takes place. Waitlist system first introduced.
11/26/2021	The German air force starts to transport patients to other parts of the country and neighboring countries due to overcrowded hospitals.
11/27/2021	The fifth of the seven announced vaccination events takes place.
11/27/2021	The federal health ministry officially confirms the first cases of Omicron in Germany and further restricts travel from other countries.

11/30/2021	Chancellor Scholz and the heads of German state governments announce that the 2G rule has become the nationwide standard and confirm plans for a vaccination mandate.
12/01/2021	The first of the additional (unannounced) vaccination events takes place.
12/04/2021	The regional health ministry of Baden-Wuerttemberg introduces the "2G+ rule", restricting access to most shops and restaurants to people who either 1) received the first and second dose or 2) recovered from COVID-19 and can provide a recent negative test.
12/08/2021	The state of Baden-Wuerttemberg opens further vaccination centers exclusively for booster vaccinations.
12/08/2021	The second of the additional (unannounced) vaccination events takes place.
12/09/2021	The third of the additional (unannounced) vaccination events takes place.
12/10/2021	The sixth of the seven announced vaccination events takes place.
12/11/2021	The seventh of the seven announced vaccination events takes place.
12/15/2021	The fourth of the additional (unannounced) vaccination events takes place.
12/16/2021	The fifth of the additional (unannounced) vaccination events takes place.
12/17/2021	The sixth of the additional (unannounced) vaccination events takes place.
12/18/2021	The seventh of the additional (unannounced) vaccination events takes place.
12/21/2021	The national government and the heads of German state governments announce stricter social restrictions. Starting on 12/28/2021, private gatherings of vaccinated and recovered persons are restricted to a maximum of ten persons; private gatherings of unvaccinated persons are limited to the person's own household and a maximum of two persons from another household. The Standing Committee on Vaccination recommends booster vaccinations for all persons older than 18 years of age, three months after the second dose.
12/22/2021	The eighth of the additional (unannounced) vaccination events takes place.
12/23/2021	The ninth of the additional (unannounced) vaccination events takes place.
12/29/2021	The tenth of the additional (unannounced) vaccination events takes place.
12/30/2021	The eleventh of the additional (unannounced) vaccination events takes place.

eAppendix 4. Types of Treatment Effects

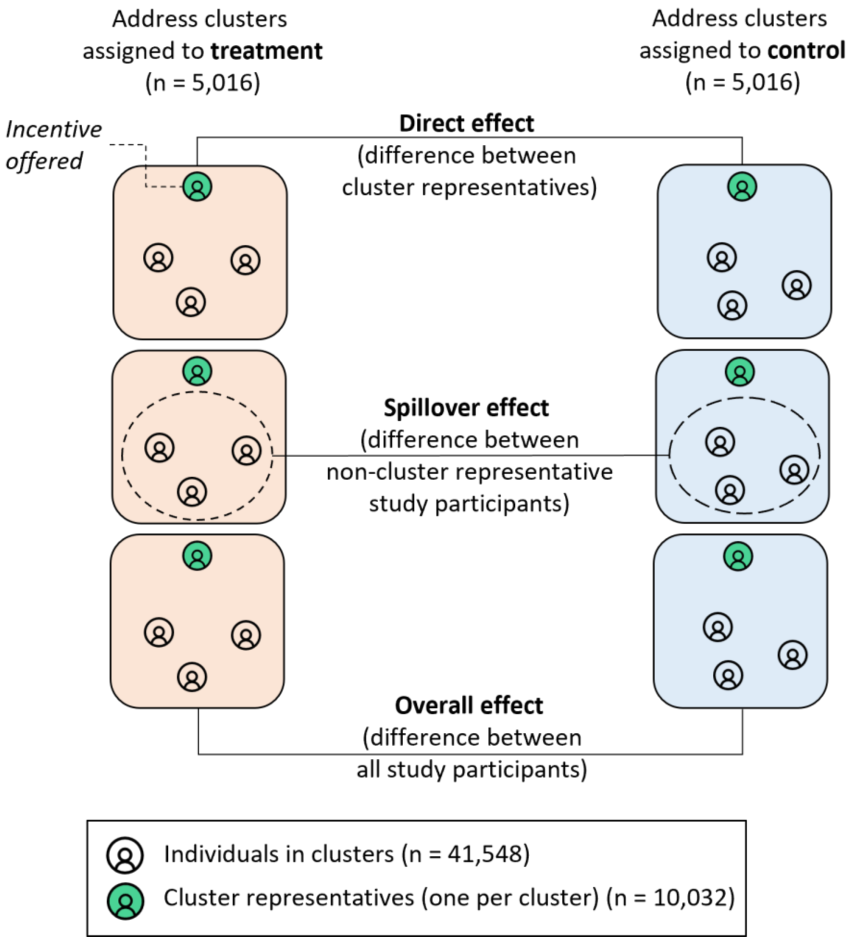

Figure 1: Illustration of the estimated effects. The figure depicts randomization into treatment (left column) and control address-clusters (right column), the randomized selection of address-cluster representatives (green circles), and the estimated parameters of interest (direct, spillover, and overall effects).

© 2025 Ternovski J et al. *JAMA Network Open*

eAppendix 5. Investigating Postponement Spillovers

Field studies looking at vaccination outcomes must consider censored data. Like most countries,[2] Germany does not have a national vaccination registry, so it is not possible to observe whether an individual was vaccinated outside of the city-organized events. Our study was initially expected to focus on only the public events announced in the letters sent out by the city. However, when city leadership decided to organize additional events, this presented us with a unique opportunity to investigate the possibility that direct or indirect exposure to the monetary incentives may make a participant more likely to get vaccinated at other venues.[3] In other words, we are able to observe outcome data that may normally be censored.

Table 2: Treatment effects by type of vaccination considering only additional vaccination events. Covariate-adjusted OLS regression estimates of the effects of the treatment on vaccinations. Effects are estimated for primary vaccinations (first and second dose) and booster vaccinations separately.

	**Primary vaccination**			**Booster vaccination**		
	(1) Direct Effect	(2) Spillover Effect	(3) Overall Effect	(4) Direct Effect	(5) Spillover Effect	(6) Overall Effect
Treatment	0.195+	-0.083	-0.015	0.033	0.161	0.133
	(0.115)	(0.078)	(0.066)	(0.215)	(0.132)	(0.116)
Age	-0.003	-0.003	-0.005	0.044	-0.009	-0.001
	(0.022)	(0.012)	(0.010)	(0.034)	(0.021)	(0.018)
Age^2	0.000	0.000	0.000	0.000	0.000	0.000
	(0.000)	(0.000)	(0.000)	(0.000)	(0.000)	(0.000)
Female	-0.015	0.044	0.023	0.036	0.156	0.106
	(0.147)	(0.075)	(0.060)	(0.250)	(0.129)	(0.103)
Non-German	1.089**	1.000***	0.998***	-0.241	-0.255	-0.303+
	(0.347)	(0.178)	(0.161)	(0.417)	(0.207)	(0.182)
Std. Errors	Hetero-skedasticity-robust	by address cluster	by address cluster	Hetero-skedasticity-robust	by address cluster	by address cluster
n	9,736	30,479	40,215	9,814	30,763	40,577

Estimates are expressed in terms of percentage points. Standard errors in parentheses. + $p < 0.1$, * $p < 0.05$, ** $p < 0.01$, *** $p < 0.001$.

In Table 2, we present our main analyses only for the additional events not advertised in the

[2] Few countries have national vaccination registries (Papagiannis et al., 2022) and the systems that do exist have fared poorly in data quality audits – "10–60% of immunization records lack important information or contain errors" (Atkinson et al., 2020).

[3] Our pre-registration plan was updated as soon as the possibility of additional events was made known to us (11/12/2021).

treatment and control letters. We are underpowered in this analysis but see suggestive evidence that the backlashes we report in the main text may be at least partially explained by displacement. Specifically, we observe a positive spillover effect for boosters of 0.2 percentage points (though it is not statistically significant). Given that our main analysis found a negative booster spillover effect of similar magnitude in the opposite direction (see Table 3 of the main text), this is suggestive evidence that individuals in the same household as someone who received a financial incentive may have postponed vaccination to a future event that was not announced in the treatment letter.

There is additional circumstantial evidence that comports with this interpretation. The first three announced events had long lines and there were cases of people being turned away due to insufficient vaccine doses. Additionally, 55.9% of attendees at the advertised events were from outside of the city of Ravensburg, which would potentially crowd out impacts of our treatment, as these individuals were never randomized. An individual residing in the same household as someone who received a monetary incentive may feel that long lines at the advertised events are indicative of people wanting to claim their monetary incentive. As such, these cohabitants of treatment letter recipients may seek to get vaccinated at other events.

The additional events are not the only alternative venues where a Ravensburg resident may seek out vaccination. Still, at the time of this study, vaccine doses were of extremely limited supply in Germany and appointments were hard to come by. To get some sense as to the availability of other vaccination options, we asked all those who attended the public vaccination events whether their physician offers COVID-19 vaccinations. Because this survey conditions on attending the public vaccination events (i.e., survey respondents are systematically different from people who didn't attend the public vaccination events), these data are likely to have high-levels of self-selection and should be viewed only as a rough benchmark of our participants' alternative vaccination options. From Table 3, we see that few participants in the survey viewed vaccination at their GP's office as a viable alternative.

Table 3: Potential displacement effects. Participants were asked whether they already planned to get vaccinated by a GP in the following weeks

Answer	Total
No or 'I do not have a GP'	272 (34.17%)
Yes	129 (16.21%)
GP has no appointments left	263 (33.04%)
GP does not vaccinate	88 (11.06%)
No answer	44 (5.53%)

To investigate whether long lines had any differential impact on a treatment household and control household members' decision to wait in line to get vaccinated, we present treatment and control vaccinations by day and time of day in Fig. 2 and 3. The results of these analyses are not clear, but there does seem to be some association between experimental conditions and the time of day the individual was vaccinated.

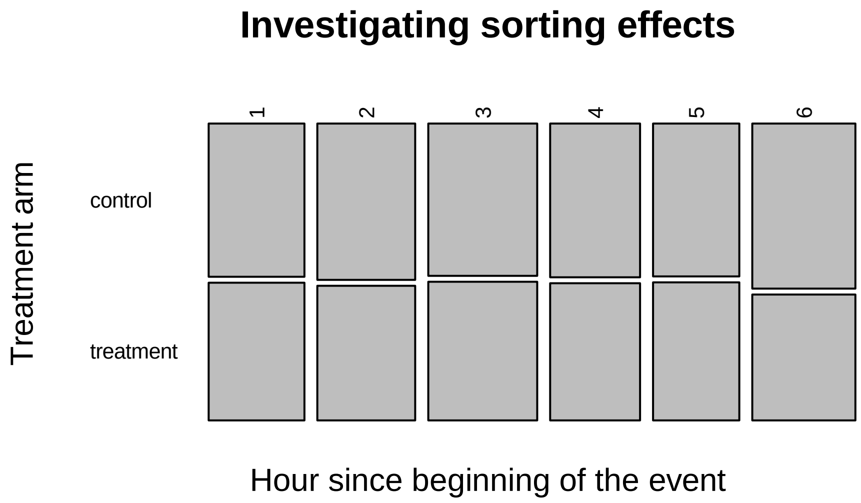

Figure 2: Distribution of vaccinations administered by treatment arm and hours since the beginning of the event. Figure considers all regular events.

We note that the first three days had long lines and there was more demand than supply of vaccine doses. The remaining days had expanded supplies of vaccines and used a waitlist. The waitlist system is described in detail above, in Section 2, Data Collection. We find that 78.6% of participants in treatment households got vaccinated after being on the waitlist compared to only 45.0% in control households (p=.109).

While the sum of this evidence is far from strong, it does provide some support for the theory that long lines may have had an effect on how the monetary incentive directly and indirectly affected vaccination behaviors.

© 2025 Ternovski J et al. *JAMA Network Open*

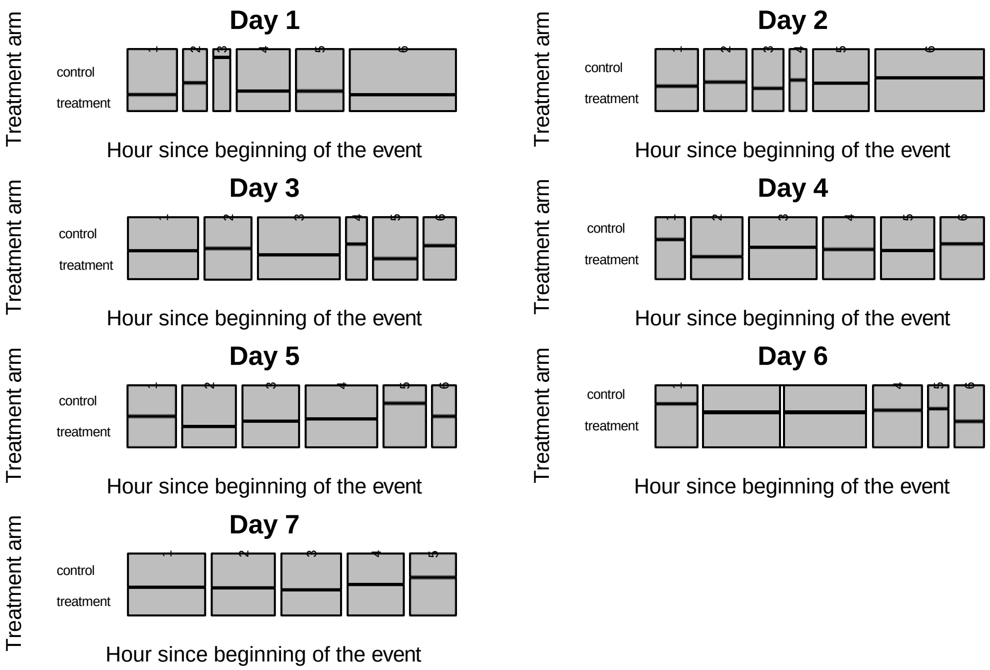

Figure 3: Daily distribution of vaccinations administered by treatment arm and hours since the beginning of the event.

eAppendix 6. Robustness Checks

1.3 Alternative Cluster Specifications

Registry address data does not include apartment numbers and, for some larger address-clusters, potential outcomes may not be correlated among residents of the same address. To address this possibility, we pre-registered an analysis that reformulated clusters to a shared address and a shared last name.[4] As seen in Tables S3-S8, this specification yields results that are broadly in line with our main analyses.

1.3.1 Same Name and Address as Clusters

Table 4: Main results with clustered standard errors defined as shared name and address. Covariate-adjusted OLS regression estimates of the effects of the treatment on vaccinations and information uptake.

	(1) Spillover effect Vaccinations	(2) Overall effect Clicks	(3) Overall effect Vaccinations
Treatment	-0.269+	0.000	-0.263+
	(0.162)	(0.147)	(0.143)
Age	0.014	0.140***	0.017
	(0.023)	(0.021)	(0.020)
Age^2	0.000	-0.001***	0.000
	(0.000)	(0.000)	(0.000)
Female	-0.018	-0.002	0.045
	(0.152)	(0.153)	(0.125)
Non-German	0.961***	0.283	1.031***
	(0.261)	(0.235)	(0.239)
Std. Errors	by name-address cluster	by name-address cluster	by name-address cluster
n	31,516	41,548	41,548

Estimates are expressed in terms of percentage points. Standard errors in parentheses. + $p < 0.1$, * $p < 0.05$, ** $p < 0.01$, *** $p < 0.001$.

Table 5 uses the same specification but estimates primary vaccinations (first and second dose) and booster vaccinations separately.

[4] This alternative is also imperfect as it does not take into account all variants of hyphenated names or co-residents with different last names.

Table 5: Main results with clustered standard errors defined as shared name and address (by vaccination type). Covariate-adjusted OLS regression estimates of the effects of the treatment on vaccinations.

	Primary vaccination		Booster vaccination	
	(1) Spillover effect	(2) Overall effect	(3) Spillover effect	(4) Overall effect
Treatment	0.021	0.032	-0.295*	-0.299**
	(0.107)	(0.090)	(0.123)	(0.112)
Age	-0.026	-0.026+	0.041*	0.043**
	(0.016)	(0.014)	(0.017)	(0.016)
Age^2	0.000	0.000	0.000	0.000
	(0.000)	(0.000)	(0.000)	(0.000)
Female	-0.228*	-0.184*	0.208+	0.231*
	(0.101)	(0.080)	(0.116)	(0.098)
Non-German	1.444***	1.460***	-0.478***	-0.420**
	(0.222)	(0.203)	(0.141)	(0.130)
Std. Errors	by name-address cluster	by name-address cluster	by name-address cluster	by name-address cluster
n	31,163	41,059	31,259	41,241

Estimates are expressed in terms of percentage points. Standard errors in parentheses. + p < 0.1, * p < 0.05, ** p < 0.01, *** p < 0.001.

Though we did not pre-register additional cluster specifications, for completeness we include two additional analyses. In the subsequent subsections, we present our main results with precinct-cluster standard errors and another analysis where we limit the sample to residents of small clusters only.

© 2025 Ternovski J et al. *JAMA Network Open*

1.3.2 Precincts as Clusters

With this specification, we use the 60 precincts in the city to calculate clustered standard errors. Table 6 provides the main results with this alternative specification.

Table 6: Main results with precincts as clusters. Covariate-adjusted OLS regression estimates of the effects of the treatment on vaccinations and information uptake.

	(1) Spillover effect Vaccinations	(2) Overall effect Clicks	(3) Overall effect Vaccinations
Treatment	-0.269	0.000	-0.263+
	(0.162)	(0.108)	(0.153)
Age	0.014	0.140***	0.017
	(0.014)	(0.009)	(0.012)
Age^2	0.000	-0.001***	0.000
	(0.000)	(0.000)	(0.000)
Female	-0.018	-0.002	0.045
	(0.096)	(0.099)	(0.076)
Non-German	0.961***	0.283+	1.031***
	(0.237)	(0.145)	(0.217)
Std. Errors	by precinct cluster	by precinct cluster	by precinct cluster
n	31,516	41,548	41,548

Estimates are expressed in terms of percentage points. Standard errors in parentheses. + p < 0.1, * p < 0.05, ** p < 0.01, *** p < 0.001.

Table 7 also uses the precincts as social networks but estimates primary vaccinations (first and second dose) and booster vaccinations separately.

Table 7: Main results with precincts as clusters (by vaccination type). Covariate-adjusted OLS regression estimates of the effects of the treatment on vaccinations.

	Primary vaccination		Booster vaccination	
	(1) Spillover effect	(2) Overall effect	(3) Spillover effect	(4) Overall effect
Treatment	0.021	0.032	-0.295***	-0.299***
	(0.125)	(0.103)	(0.075)	(0.082)
Age	-0.026*	-0.026**	0.041***	0.043***
	(0.011)	(0.010)	(0.011)	(0.010)
Age^2	0.000	0.000	0.000*	0.000*
	(0.000)	(0.000)	(0.000)	(0.000)
Female	-0.228***	-0.184***	0.208*	0.231**
	(0.041)	(0.038)	(0.101)	(0.071)
Non-German	1.444***	1.460***	-0.478***	-0.420***
	(0.245)	(0.223)	(0.043)	(0.045)
Std. Errors	by precinct cluster	by precinct cluster	by precinct cluster	by precinct cluster
n	31,163	41,059	31,259	41,241

Estimates are expressed in terms of percentage points. Standard errors in parentheses. + $p < 0.1$, * $p < 0.05$, ** $p < 0.01$, *** $p < 0.001$.

1.3.3 Small Clusters

With this alternative specification, we restricted the sample to addresses with less than five residents. Table 8 depicts the main results.

Table 8: Main results with small clusters (less than five members) only. Covariate-adjusted OLS regression estimates of the effects of the treatment on vaccinations and information uptake.

	(1) Spillover effect Vaccinations	(2) Overall effect Clicks	(3) Overall effect Vaccinations
Treatment	-0.516+	-0.443*	-0.501*
	(0.266)	(0.220)	(0.213)
Age	0.035	0.132***	0.040
	(0.041)	(0.036)	(0.032)
Age^2	0.000	-0.001***	0.000
	(0.000)	(0.000)	(0.000)
Female	0.398	-0.028	0.330+
	(0.282)	(0.244)	(0.186)
Non-German	0.700	0.695	0.944+
	(0.598)	(0.523)	(0.502)
Std. Errors	by address-cluster	by address-cluster	by address-cluster
n	10,130	17,658	17,658

Estimates are expressed in terms of percentage points. Standard errors in parentheses. + p < 0.1, * p < 0.05, ** p < 0.01, *** p < 0.001.

Table 9 shows the results for primary vaccinations (first and second dose) and booster vaccinations separately.

Table 9: Main results with small clusters (less than five members) only (by vaccination type). Covariate-adjusted OLS regression estimates of the effects of the treatment on vaccinations.

	**Primary vaccination**		**Booster vaccination**	
	(1) Spillover effect	(2) Overall effect	(3) Spillover effect	(4) Overall effect
Treatment	0.228	0.113	-0.738**	-0.612**
	(0.142)	(0.105)	(0.227)	(0.187)
Age	-0.041	-0.035+	0.077*	0.074**
	(0.026)	(0.019)	(0.032)	(0.026)
Age^2	0.000	0.000	0.000	0.000+
	(0.000)	(0.000)	(0.000)	(0.000)
Female	-0.064	-0.023	0.465*	0.363*
	(0.164)	(0.099)	(0.233)	(0.159)
Non-German	1.241*	1.441***	-0.564	-0.499+
	(0.483)	(0.417)	(0.358)	(0.290)
Std. Errors	by address-cluster	by address-cluster	by address-cluster	by address-cluster
n	9,996	17,409	10,075	17,574

Estimates are expressed in terms of percentage points. Standard errors in parentheses. + p < 0.1, * p < 0.05, ** p < 0.01, *** p < 0.001.

Though our analysis of small clusters reduces our sample size, we recover a negative spillover and overall effect with a smaller p-value than in our main analysis. This evidence supports the interpretation that social interactions between recipients of the treatment letter and the control led to fewer control-letter cohabitants of treatment-letter recipients seeking vaccination at the advertised public events. As mentioned in the main text, there is suggestive evidence that these individuals sought out other vaccination opportunities, such as with the additional events not mentioned in the letters.

1.4 Alternative methods to code vaccination

As mentioned in Section 2, the city organized eleven additional vaccination events during and after the study period. These additional events were not mentioned in the initial letter, however, starting on November 15th, 2021, residents who visited the website or called the information hotline could learn about these additional events. 714 residents attended and were vaccinated at one of these additional events. In our main analysis, vaccination at these events is coded as unvaccinated. To illustrate the robustness of our main analysis, we use all possible methods to code vaccination here.

The following tables replicate our main analyses including all individuals who were vaccinated at additional events (i.e., if an individual was vaccinated at an additional event their vaccination status is set =1 instead of zero, as in our main analysis.)

Table 10: Treatment effects with regular and additional vaccination events coded as successful vaccinations. Covariate-adjusted OLS regression estimates of the effects of the treatment on vaccinations.

	(1) Direct effect	(2) Spillover effect	(3) Overall effect
Treatment	-0.014	-0.200	-0.155
	(0.356)	(0.232)	(0.203)
Age	0.073	0.001	0.011
	(0.061)	(0.033)	(0.028)
Age^2	0.000	0.000	0.000
	(0.001)	(0.000)	(0.000)
Female	0.137	0.163	0.167
	(0.417)	(0.209)	(0.169)
Non-German	2.065**	1.674***	1.696***
	(0.782)	(0.399)	(0.361)
Std. Errors	Hetero-skedasticity-robust	by address cluster	by address cluster
n	10,032	31,516	41,548

Estimates are expressed in terms of percentage points. Standard errors in parentheses. + p < 0.1, * p < 0.05, ** p < 0.01, *** p < 0.001.

© 2025 Ternovski J et al. *JAMA Network Open*

Table 11: Treatment effects with regular and additional vaccination events coded as successful vaccinations (by vaccination type). Covariate-adjusted OLS regression estimates of the effects of the treatment on vaccinations. Effects are estimated for primary vaccinations (first and second dose) and booster vaccinations separately.

	Primary vaccination			Booster vaccination		
	(1) Direct Effect	(2) Spillover Effect	(3) Overall Effect	(4) Direct Effect	(5) Spillover Effect	(6) Overall Effect
Treatment	0.256	-0.063	0.014	-0.267	-0.132	-0.164
	(0.184)	(0.153)	(0.127)	(0.314)	(0.178)	(0.160)
Age	-0.030	-0.029	-0.031+	0.105*	0.033	0.042+
	(0.034)	(0.019)	(0.017)	(0.052)	(0.027)	(0.024)
Age^2	0.000	0.000	0.000	-0.001	0.000	0.000
	(0.000)	(0.000)	(0.000)	(0.000)	(0.000)	(0.000)
Female	-0.176	-0.177	-0.155	0.323	0.350*	0.333*
	(0.226)	(0.124)	(0.100)	(0.360)	(0.171)	(0.140)
Non-German	2.554***	2.426***	2.442***	-0.469	-0.716**	-0.709**
	(0.574)	(0.325)	(0.297)	(0.563)	(0.250)	(0.224)
Std. Errors	Hetero-skedasticity-robust	by address cluster	by address cluster	Hetero-skedasticity-robust	by address cluster	by address cluster
n	9,786	30,738	40,524	9,950	31,118	41,068

Estimates are expressed in terms of percentage points. Standard errors in parentheses. + p < 0.1, * p < 0.05, ** p < 0.01, *** p < 0.001.

1.5 P-value robustness

An alternative method to estimate p-values for our main analysis would be to use randomization inference (as described in e.g., Gerber & Green, 2012). The results are shown in Table 12.

Table 12: Randomization inference p-values. Results of 1,000 iterations of randomly permuting the treatment assignment vector, while keeping the rest of our data frame fixed

Effect Type	p-value
Direct effect (information uptake)	0.190
Direct effect (vaccinations)	0.198
Spillover effect (vaccinations)	0.075
Overall effect (information uptake)	0.483
Overall effect (vaccinations)	0.061

1.6 Treatment Effects Over Time

As pre-registered, we correct for multiple comparisons using the Holm-Bonferroni correction.

1.6.1 Effects by Weekends

Table 13: Direct treatment effects for each vaccination event weekend. Covariate- adjusted OLS regression.

	(1) Weekend 1	(2) Weekend 2	(3) Weekend 3	(4) Weekend 4
Treatment	-0.064	-0.048	-0.007	-0.122
	(0.120)	(0.150)	(0.144)	(0.135)
Age	0.022	0.004	0.012	-0.003
	(0.020)	(0.027)	(0.026)	(0.024)
Age^2	0.000	0.000	0.000	0.000
	(0.000)	(0.000)	(0.000)	(0.000)
Female	0.212	-0.010	-0.178	0.084
	(0.138)	(0.172)	(0.179)	(0.143)
Non-German	0.121	0.056	-0.070	1.175**
	(0.192)	(0.295)	(0.330)	(0.359)
n	9,881	9,901	9,896	9,892

Estimates are expressed in terms of percentage points. Heteroskedasticity-robust standard errors in parentheses. + p < 0.1, * p < 0.05, ** p < 0.01, *** p < 0.001.

© 2025 Ternovski J et al. *JAMA Network Open*

Table 14: Direct treatment effects on primary vaccinations for each vaccination event weekend. Covariate-adjusted OLS regression.

	(1) Weekend 1	(2) Weekend 2	(3) Weekend 3	(4) Weekend 4
Treatment	-0.042	0.115	0.034	-0.052
	(0.050)	(0.085)	(0.076)	(0.069)
Age	-0.009	-0.001	-0.004	-0.014
	(0.009)	(0.016)	(0.015)	(0.012)
Age^2	0.000	0.000	0.000	0.000
	(0.000)	(0.000)	(0.000)	(0.000)
Female	0.007	-0.138	-0.043	-0.003
	(0.051)	(0.108)	(0.102)	(0.076)
Non-German	0.244	0.216	0.232	0.842*
	(0.154)	(0.223)	(0.253)	(0.284)
n	9,852	9,864	9,860	9,858

Estimates are expressed in terms of percentage points. Heteroskedasticity-robust standard errors in parentheses. + p < 0.1, * p < 0.05, ** p < 0.01, *** p < 0.001.

© 2025 Ternovski J et al. *JAMA Network Open*

Table 15: Direct treatment effects on booster vaccinations for each vaccination event weekend. Covariate-adjusted OLS regression.

	(1)<br>Weekend 1	(2)<br>Weekend 2	(3)<br>Weekend 3	(4)<br>Weekend 4
Treatment	-0.022	-0.164	-0.040	-0.084
	(0.110)	(0.123)	(0.122)	(0.119)
Age	0.031	0.005	0.016	0.010
	(0.018)	(0.022)	(0.021)	(0.020)
Age^2	0.000	0.000	0.000	0.000
	(0.000)	(0.000)	(0.000)	(0.000)
Female	0.205	0.127	-0.136	0.097
	(0.129)	(0.134)	(0.148)	(0.126)
Non-German	-0.124	-0.160	-0.305	0.366
	(0.115)	(0.193)	(0.213)	(0.225)
n	9,875	9,883	9,882	9,880

Estimates are expressed in terms of percentage points. Heteroskedasticity-robust standard errors in parentheses. + p < 0.1, * p < 0.05, ** p < 0.01, *** p < 0.001.

© 2025 Ternovski J et al. *JAMA Network Open*

Table 16: Spillover treatment effects for each vaccination event weekend. Covariate-adjusted OLS regression.

	(1)<br>Weekend 1	(2)<br>Weekend 2	(3)<br>Weekend 3	(4)<br>Weekend 4
Treatment	-0.287*	-0.090	-0.045	0.139
	(0.102)	(0.077)	(0.083)	(0.082)
Age	0.013	-0.006	0.020	-0.013
	(0.010)	(0.012)	(0.013)	(0.013)
Age^2	0.000	0.000	0.000	0.000
	(0.000)	(0.000)	(0.000)	(0.000)
Female	0.012	-0.007	-0.044	0.015
	(0.064)	(0.076)	(0.089)	(0.078)
Non-German	0.136	0.216	0.246	0.405*
	(0.191)	(0.143)	(0.150)	(0.146)
Std. Errors	by address cluster	by address cluster	by address cluster	by address cluster
n	31,010	31,058	31,099	31,067

Estimates are expressed in terms of percentage points. Standard errors (clustered by address clusters) in parentheses. + $p < 0.1$, * $p < 0.05$, ** $p < 0.01$, *** $p < 0.001$.

© 2025 Ternovski J et al. *JAMA Network Open*

Table 17: Spillover treatment effects on primary vaccinations for each vaccination event weekend. Covariate-adjusted OLS regression.

	(1)<br>Weekend 1	(2)<br>Weekend 2	(3)<br>Weekend 3	(4)<br>Weekend 4
Treatment	-0.116	0.049	0.029	0.060
	(0.093)	(0.049)	(0.057)	(0.047)
Age	-0.003	-0.013	0.008	-0.019+
	(0.006)	(0.009)	(0.009)	(0.008)
Age^2	0.000	0.000	0.000	0.000
	(0.000)	(0.000)	(0.000)	(0.000)
Female	-0.053	-0.022	-0.102	-0.056
	(0.045)	(0.049)	(0.060)	(0.048)
Non-German	0.244	0.365**	0.369**	0.510***
	(0.188)	(0.122)	(0.126)	(0.120)
n	30,956	30,969	30,992	30,964

Estimates are expressed in terms of percentage points. Standard errors (clustered by address clusters) in parentheses. + $p < 0.1$, * $p < 0.05$, ** $p < 0.01$, *** $p < 0.001$.

Table 18: Spillover treatment effects on booster vaccinations for each vaccination event weekend. Covariate-adjusted OLS regression.

	(1) Weekend 1	(2) Weekend 2	(3) Weekend 3	(4) Weekend 4
Treatment	-0.172***	-0.141+	-0.075	0.080
	(0.044)	(0.060)	(0.062)	(0.066)
Age	0.015	0.007	0.012	0.007
	(0.008)	(0.009)	(0.009)	(0.010)
Age^2	0.000	0.000	0.000	0.000
	(0.000)	(0.000)	(0.000)	(0.000)
Female	0.065	0.015	0.057	0.070
	(0.046)	(0.058)	(0.066)	(0.061)
Non-German	-0.107*	-0.146	-0.122	-0.104
	(0.036)	(0.075)	(0.083)	(0.082)
n	30,960	30,995	31,013	31,009

Estimates are expressed in terms of percentage points. Standard errors (clustered by address clusters) in parentheses. + p < 0.1, * p < 0.05, ** p < 0.01, *** p < 0.001.

© 2025 Ternovski J et al. *JAMA Network Open*

Table 19: Overall treatment effects for each vaccination event weekend. Covariate- adjusted OLS regression.

	(1) Weekend 1	(2) Weekend 2	(3) Weekend 3	(4) Weekend 4
Treatment	-0.232*	-0.083	-0.037	0.078
	(0.086)	(0.071)	(0.074)	(0.072)
Age	0.014	-0.004	0.016	-0.009
	(0.009)	(0.011)	(0.011)	(0.011)
Age^2	0.000	0.000	0.000	0.000
	(0.000)	(0.000)	(0.000)	(0.000)
Female	0.060	0.025	-0.066	0.025
	(0.056)	(0.064)	(0.071)	(0.063)
Non-German	0.124	0.200	0.222	0.524***
	(0.173)	(0.127)	(0.135)	(0.141)
Std. Errors	by address cluster	by address cluster	by address cluster	by address cluster
n	40,891	40,959	40,995	40,959

Estimates are expressed in terms of percentage points. Standard errors (clustered by address clusters) in parentheses. + $p < 0.1$, * $p < 0.05$, ** $p < 0.01$, *** $p < 0.001$.

© 2025 Ternovski J et al. *JAMA Network Open*

Table 20: Overall treatment effects on primary vaccinations for each vaccination event weekend. Covariate-adjusted OLS regression.

	(1) Weekend 1	(2) Weekend 2	(3) Weekend 3	(4) Weekend 4
Treatment	-0.097	0.062	0.031	0.036
	(0.075)	(0.043)	(0.047)	(0.041)
Age	-0.004	-0.010	0.004	-0.017+
	(0.005)	(0.007)	(0.008)	(0.007)
Age^2	0.000	0.000	0.000	0.000
	(0.000)	(0.000)	(0.000)	(0.000)
Female	-0.033	-0.035	-0.077	-0.044
	(0.036)	(0.041)	(0.047)	(0.037)
Non-German	0.239	0.345**	0.360**	0.558***
	(0.169)	(0.108)	(0.113)	(0.116)
n	40,808	40,833	40,852	40,822

Estimates are expressed in terms of percentage points. Standard errors (clustered by address clusters) in parentheses. + p < 0.1, * p < 0.05, ** p < 0.01, *** p < 0.001.

© 2025 Ternovski J et al. *JAMA Network Open*

Table 21: Overall treatment effects on booster vaccinations for each vaccination event weekend. Covariate-adjusted OLS regression.

	(1)<br>Weekend 1	(2)<br>Weekend 2	(3)<br>Weekend 3	(4)<br>Weekend 4
Treatment	-0.135**	-0.147*	-0.069	0.042
	(0.042)	(0.056)	(0.058)	(0.059)
Age	0.019*	0.007	0.011	0.007
	(0.007)	(0.008)	(0.008)	(0.009)
Age²	0.000	0.000	0.000	0.000
	(0.000)	(0.000)	(0.000)	(0.000)
Female	0.092	0.061	0.011	0.069
	(0.043)	(0.050)	(0.053)	(0.050)
Non-German	-0.115**	-0.143+	-0.138	-0.030
	(0.034)	(0.067)	(0.074)	(0.080)
n	40,835	40,878	40,895	40,889

Estimates are expressed in terms of percentage points. Standard errors (clustered by address clusters) in parentheses. + p < 0.1, * p < 0.05, ** p < 0.01, *** p < 0.001.

1.6.2 Effects by Day

Table 22: Direct treatment effects for each vaccination event. Covariate-adjusted OLS regression.

	(1) Event 1	(2) Event 2	(3) Event 3	(4) Event 4	(5) Event 5	(6) Event 6	(7) Event 7
Treatment	-0.064	-0.033	-0.015	0.020	-0.027	0.004	-0.139
	(0.120)	(0.103)	(0.109)	(0.114)	(0.088)	(0.099)	(0.096)
Age	0.022	0.011	-0.007	0.013	-0.001	0.001	-0.005
	(0.020)	(0.016)	(0.022)	(0.019)	(0.017)	(0.017)	(0.017)
Age^2	0.000	0.000	0.000	0.000	0.000	0.000	0.000
	(0.000)	(0.000)	(0.000)	(0.000)	(0.000)	(0.000)	(0.000)
Female	0.212	-0.269	0.259	-0.123	-0.056	0.025	0.069
	(0.138)	(0.110)	(0.132)	(0.148)	(0.101)	(0.108)	(0.100)
Non-German	0.121	-0.087	0.144	-0.214	0.142	0.540	0.670
	(0.192)	(0.224)	(0.192)	(0.232)	(0.237)	(0.234)	(0.278)
n	9,881	9,872	9,875	9,877	9,865	9,870	9,868

Estimates are expressed in terms of percentage points. Heteroskedasticity-robust standard errors in parentheses. + $p < 0.1$, * $p < 0.05$, ** $p < 0.01$, *** $p < 0.001$.

© 2025 Ternovski J et al. *JAMA Network Open*

Table 23: Direct treatment effects on primary vaccinations for each vaccination event. Covariate-adjusted OLS regression.

	(1) Event 1	(2) Event 2	(3) Event 3	(4) Event 4	(5) Event 5	(6) Event 6	(7) Event 7
Treatment	-0.042	0.082	0.033	0.041	-0.007	0.005	-0.057
	(0.050)	(0.058)	(0.063)	(0.041)	(0.064)	(0.038)	(0.057)
Age	-0.009	0.002	-0.003	0.004	-0.008	-0.011	-0.003
	(0.009)	(0.008)	(0.014)	(0.005)	(0.014)	(0.008)	(0.009)
Age^2	0.000	0.000	0.000	0.000	0.000	0.000	0.000
	(0.000)	(0.000)	(0.000)	(0.000)	(0.000)	(0.000)	(0.000)
Female	0.007	-0.162	0.024	-0.028	-0.015	-0.071	0.068
	(0.051)	(0.072)	(0.080)	(0.057)	(0.084)	(0.042)	(0.063)
Non-German	0.244	0.014	0.203	0.001	0.231	0.359	0.487
	(0.154)	(0.137)	(0.177)	(0.119)	(0.224)	(0.178)	(0.224)
n	9,852	9,854	9,856	9,850	9,856	9,850	9,854

Estimates are expressed in terms of percentage points. Heteroskedasticity-robust standard errors in parentheses. + $p < 0.1$, * $p < 0.05$, ** $p < 0.01$, *** $p < 0.001$.

© 2025 Ternovski J et al. *JAMA Network Open*

Table 24: Direct treatment effects on booster vaccinations for each vaccination event. Covariate-adjusted OLS regression.

	(1) Event 1	(2) Event 2	(3) Event 3	(4) Event 4	(5) Event 5	(6) Event 6	(7) Event 7
Treatment	-0.022	-0.116	-0.048	-0.020	-0.020	-0.002	-0.083
	(0.110)	(0.086)	(0.089)	(0.106)	(0.061)	(0.091)	(0.077)
Age	0.031	0.009	-0.004	0.009	0.007	0.012	-0.002
	(0.018)	(0.014)	(0.017)	(0.019)	(0.009)	(0.015)	(0.014)
Age^2	0.000	0.000	0.000	0.000	0.000	0.000	0.000
	(0.000)	(0.000)	(0.000)	(0.000)	(0.000)	(0.000)	(0.000)
Female	0.205	-0.108	0.235	-0.095	-0.042	0.096	0.001
	(0.129)	(0.083)	(0.105)	(0.137)	(0.057)	(0.099)	(0.077)
Non-German	-0.124	-0.102	-0.059	-0.216	-0.090	0.182	0.184
	(0.115)	(0.178)	(0.075)	(0.200)	(0.075)	(0.152)	(0.167)
n	9,875	9,864	9,865	9,873	9,855	9,866	9,860

Estimates are expressed in terms of percentage points. Heteroskedasticity-robust standard errors in parentheses. + p < 0.1, * p < 0.05, ** p < 0.01, *** p < 0.001.

© 2025 Ternovski J et al. *JAMA Network Open*

Table 25: Spillover treatment effects for each vaccination event. Covariate-adjusted OLS regression.

	(1) Event 1	(2) Event 2	(3) Event 3	(4) Event 4	(5) Event 5	(6) Event 6	(7) Event 7
Treatment	-0.287*	-0.030	-0.062	-0.022	-0.025	0.126	0.016
	(0.102)	(0.058)	(0.052)	(0.063)	(0.055)	(0.063)	(0.052)
Age	0.013	-0.004	-0.002	0.013	0.007	0.000	-0.013
	(0.010)	(0.009)	(0.009)	(0.010)	(0.009)	(0.010)	(0.008)
Age^2	0.000	0.000	0.000	0.000	0.000	0.000	0.000
	(0.000)	(0.000)	(0.000)	(0.000)	(0.000)	(0.000)	(0.000)
Female	0.012	-0.009	0.002	-0.011	-0.033	0.020	-0.003
	(0.064)	(0.054)	(0.053)	(0.067)	(0.059)	(0.061)	(0.049)
Non-German	0.136	0.014	0.205	0.208	0.040	0.093	0.314*
	(0.191)	(0.094)	(0.109)	(0.117)	(0.092)	(0.100)	(0.105)
n	31,010	30,986	30,978	31,019	30,986	31,003	30,970

Estimates are expressed in terms of percentage points. Standard errors (clustered by address clusters) in parentheses. + $p < 0.1$, * $p < 0.05$, ** $p < 0.01$, *** $p < 0.001$.

Table 26: Spillover treatment effects on primary vaccinations for each vaccination event. Covariate-adjusted OLS regression.

	(1)<br>Event 1	(2)<br>Event 2	(3)<br>Event 3	(4)<br>Event 4	(5)<br>Event 5	(6)<br>Event 6	(7)<br>Event 7
Treatment	-0.116	0.020	0.029	0.027	0.003	0.023	0.036
	(0.093)	(0.030)	(0.039)	(0.044)	(0.037)	(0.038)	(0.028)
Age	-0.003	-0.008	-0.006	0.005	0.003	-0.006	-0.014*
	(0.006)	(0.005)	(0.007)	(0.007)	(0.006)	(0.006)	(0.005)
Age^2	0.000	0.000	0.000	0.000	0.000	0.000	0.000*
	(0.000)	(0.000)	(0.000)	(0.000)	(0.000)	(0.000)	(0.000)
Female	-0.053	0.006	-0.028	-0.061	-0.042	-0.005	-0.050
	(0.045)	(0.028)	(0.040)	(0.044)	(0.042)	(0.038)	(0.029)
Non-	0.244	0.107	0.259+	0.271*	0.101	0.177	0.336***
German	(0.188)	(0.070)	(0.101)	(0.100)	(0.074)	(0.079)	(0.087)
n	30,956	30,929	30,946	30,956	30,942	30,943	30,927

Estimates are expressed in terms of percentage points. Standard errors (clustered by address clusters) in parentheses. + $p < 0.1$, * $p < 0.05$, ** $p < 0.01$, *** $p < 0.001$.

© 2025 Ternovski J et al. *JAMA Network Open*

Table 27: Spillover treatment effects on booster vaccinations for each vaccination event. Covariate-adjusted OLS regression.

	(1)<br>Event 1	(2)<br>Event 2	(3)<br>Event 3	(4)<br>Event 4	(5)<br>Event 5	(6)<br>Event 6	(7)<br>Event 7
Treatment	-0.172***	-0.050	-0.091*	-0.049	-0.028	0.104	-0.021
	(0.044)	(0.049)	(0.034)	(0.046)	(0.041)	(0.049)	(0.043)
Age	0.015	0.004	0.003	0.008	0.004	0.006	0.001
	(0.008)	(0.007)	(0.005)	(0.007)	(0.006)	(0.007)	(0.007)
Age^2	0.000	0.000	0.000	0.000	0.000	0.000	0.000
	(0.000)	(0.000)	(0.000)	(0.000)	(0.000)	(0.000)	(0.000)
Female	0.065	-0.015	0.030	0.049	0.009	0.025	0.047
	(0.046)	(0.046)	(0.036)	(0.051)	(0.042)	(0.047)	(0.041)
Non-German	-0.107*	-0.093	-0.054	-0.063	-0.061	-0.084	-0.021
	(0.036)	(0.063)	(0.042)	(0.062)	(0.055)	(0.064)	(0.052)
n	30,960	30,963	30,938	30,969	30,950	30,966	30,949

Estimates are expressed in terms of percentage points. Standard errors (clustered by address clusters) in parentheses. + $p < 0.1$, * $p < 0.05$, ** $p < 0.01$, *** $p < 0.001$.

© 2025 Ternovski J et al. *JAMA Network Open*

Table 28: Overall treatment effects for each vaccination event. Covariate-adjusted OLS regression.

	(1)<br>Event 1	(2)<br>Event 2	(3)<br>Event 3	(4)<br>Event 4	(5)<br>Event 5	(6)<br>Event 6	(7)<br>Event 7
Treatment	-0.232*	-0.032	-0.054	-0.014	-0.025	0.097	-0.019
	(0.086)	(0.053)	(0.048)	(0.057)	(0.047)	(0.055)	(0.047)
Age	0.014	0.000	-0.004	0.011	0.005	0.001	-0.010
	(0.009)	(0.008)	(0.008)	(0.009)	(0.007)	(0.008)	(0.007)
Age^2	0.000	0.000	0.000	0.000	0.000	0.000	0.000
	(0.000)	(0.000)	(0.000)	(0.000)	(0.000)	(0.000)	(0.000)
Female	0.060	-0.042	0.068	-0.034	-0.031	0.016	0.011
	(0.056)	(0.044)	(0.047)	(0.055)	(0.046)	(0.049)	(0.040)
Non-German	0.124	0.009	0.194	0.156	0.069	0.151	0.378**
	(0.173)	(0.087)	(0.094)	(0.105)	(0.083)	(0.097)	(0.102)
n	40,891	40,858	40,853	40,896	40,851	40,873	40,838

Estimates are expressed in terms of percentage points. Standard errors (clustered by address clusters) in parentheses. + p < 0.1, * p < 0.05, ** p < 0.01, *** p < 0.001.

Table 29: Overall treatment effects on primary vaccinations for each vaccination event. Covariate-adjusted OLS regression.

	(1) Event 1	(2) Event 2	(3) Event 3	(4) Event 4	(5) Event 5	(6) Event 6	(7) Event 7
Treatment	-0.097	0.034	0.028	0.030	0.002	0.019	0.017
	(0.075)	(0.027)	(0.034)	(0.035)	(0.032)	(0.030)	(0.026)
Age	-0.004	-0.005	-0.005	0.004	0.000	-0.007	-0.010
	(0.005)	(0.005)	(0.006)	(0.006)	(0.005)	(0.005)	(0.004)
Age^2	0.000	0.000	0.000	0.000	0.000	0.000	0.000
	(0.000)	(0.000)	(0.000)	(0.000)	(0.000)	(0.000)	(0.000)
Female	-0.033	-0.034	-0.002	-0.054	-0.023	-0.016	-0.027
	(0.036)	(0.025)	(0.033)	(0.034)	(0.032)	(0.029)	(0.022)
Non-German	0.239	0.094	0.252*	0.233*	0.130	0.199*	0.362***
	(0.169)	(0.066)	(0.087)	(0.088)	(0.069)	(0.076)	(0.086)
n	40,808	40,783	40,802	40,806	40,798	40,793	40,781

Estimates are expressed in terms of percentage points. Standard errors (clustered by address clusters) in parentheses. + $p < 0.1$, * $p < 0.05$, ** $p < 0.01$, *** $p < 0.001$.

Table 30: Overall treatment effects on booster vaccinations for each vaccination event. Covariate-adjusted OLS regression.

	(1) Event 1	(2) Event 2	(3) Event 3	(4) Event 4	(5) Event 5	(6) Event 6	(7) Event 7
Treatment	-0.135**	-0.066	-0.082+	-0.044	-0.026	0.079	-0.035
	(0.042)	(0.045)	(0.033)	(0.045)	(0.035)	(0.045)	(0.038)
Age	0.019*	0.006	0.001	0.007	0.005	0.007	0.000
	(0.007)	(0.006)	(0.005)	(0.007)	(0.005)	(0.007)	(0.006)
Age^2	0.000	0.000	0.000	0.000	0.000	0.000	0.000
	(0.000)	(0.000)	(0.000)	(0.000)	(0.000)	(0.000)	(0.000)
Female	0.092	-0.008	0.070	0.019	-0.008	0.031	0.038
	(0.043)	(0.037)	(0.034)	(0.042)	(0.033)	(0.038)	(0.034)
Non-German	-0.115**	-0.085	-0.058	-0.077	-0.061	-0.047	0.017
	(0.034)	(0.057)	(0.035)	(0.059)	(0.047)	(0.063)	(0.051)
n	40,835	40,827	40,803	40,842	40,805	40,832	40,809

Estimates are expressed in terms of percentage points. Standard errors (clustered by address clusters) in parentheses. + p < 0.1, * p < 0.05, ** p < 0.01, *** p < 0.001.

1.6.3 Effects by Day (Cumulative)

Table 31: Direct treatment effects for each vaccination event (Cumulative). Covariate-adjusted OLS regression.

	(1) Event 1	(2) Event 2	(3) Event 3	(4) Event 4	(5) Event 5	(6) Event 6	(7) Event 7
Treatment	-0.064	-0.096	-0.124	-0.103	-0.130	-0.126	-0.247
	(0.120)	(0.158)	(0.190)	(0.220)	(0.236)	(0.254)	(0.269)
Age	0.022	0.033	0.025	0.038	0.037	0.037	0.033
	(0.020)	(0.026)	(0.034)	(0.039)	(0.042)	(0.045)	(0.048)
Age^2	0.000	0.000	0.000	0.000	0.000	0.000	0.000
	(0.000)	(0.000)	(0.000)	(0.000)	(0.000)	(0.000)	(0.000)
Female	0.212	-0.057	0.192	0.069	0.014	0.054	0.112
	(0.138)	(0.176)	(0.219)	(0.262)	(0.280)	(0.297)	(0.310)
Non-German	0.121	0.033	0.174	-0.038	0.105	0.611	1.235
	(0.192)	(0.295)	(0.350)	(0.418)	(0.477)	(0.526)	(0.587)
n	9,881	9,907	9,936	9,967	9,986	10,010	10,032

Estimates are expressed in terms of percentage points. Heteroskedasticity-robust standard errors in parentheses. + $p < 0.1$, * $p < 0.05$, ** $p < 0.01$, *** $p < 0.001$.

Table 32: Direct treatment effects on primary vaccinations for each vaccination event (Cumulative). Covariate-adjusted OLS regression.

	(1) Event 1	(2) Event 2	(3) Event 3	(4) Event 4	(5) Event 5	(6) Event 6	(7) Event 7
Treatment	-0.042	0.041	0.074	0.114	0.107	0.113	0.056
	(0.050)	(0.076)	(0.099)	(0.107)	(0.124)	(0.130)	(0.142)
Age	-0.009	-0.007	-0.010	-0.006	-0.013	-0.025	-0.028
	(0.009)	(0.012)	(0.018)	(0.019)	(0.023)	(0.024)	(0.026)
Age²	0.000	0.000	0.000	0.000	0.000	0.000	0.000
	(0.000)	(0.000)	(0.000)	(0.000)	(0.000)	(0.000)	(0.000)
Female	0.007	-0.155	-0.131	-0.159	-0.173	-0.226	-0.159
	(0.051)	(0.088)	(0.119)	(0.132)	(0.156)	(0.159)	(0.171)
Non-German	0.244	0.257	0.458	0.459	0.685	1.010+	1.483**
	(0.154)	(0.206)	(0.270)	(0.295)	(0.368)	(0.404)	(0.457)
n	9,852	9,860	9,870	9,874	9,884	9,888	9,896

Estimates are expressed in terms of percentage points. Heteroskedasticity-robust standard errors in parentheses. + p < 0.1, * p < 0.05, ** p < 0.01, *** p < 0.001.

© 2025 Ternovski J et al. *JAMA Network Open*

Table 33: Direct treatment effects on booster vaccinations for each vaccination event (Cumulative). Covariate-adjusted OLS regression.

	(1) Event 1	(2) Event 2	(3) Event 3	(4) Event 4	(5) Event 5	(6) Event 6	(7) Event 7
Treatment	-0.022	-0.137	-0.198	-0.217	-0.237	-0.237	-0.319
	(0.110)	(0.139)	(0.163)	(0.193)	(0.202)	(0.221)	(0.233)
Age	0.031	0.040	0.035	0.044	0.051	0.063	0.061
	(0.018)	(0.023)	(0.029)	(0.034)	(0.035)	(0.038)	(0.040)
Age^2	0.000	0.000	0.000	0.000	0.000	0.000	0.000
	(0.000)	(0.000)	(0.000)	(0.000)	(0.000)	(0.000)	(0.000)
Female	0.205	0.098	0.322	0.227	0.185	0.279	0.279
	(0.129)	(0.153)	(0.185)	(0.228)	(0.235)	(0.254)	(0.264)
Non-German	-0.124	-0.225	-0.286	-0.501	-0.589	-0.407	-0.224
	(0.115)	(0.212)	(0.225)	(0.299)	(0.308)	(0.343)	(0.380)
n	9,875	9,893	9,912	9,939	9,948	9,968	9,982

Estimates are expressed in terms of percentage points. Heteroskedasticity-robust standard errors in parentheses. + $p < 0.1$, * $p < 0.05$, ** $p < 0.01$, *** $p < 0.001$.

© 2025 Ternovski J et al. *JAMA Network Open*

Table 34: Spillover treatment effects for each vaccination event (Cumulative). Covariate-adjusted OLS regression.

	(1) Event 1	(2) Event 2	(3) Event 3	(4) Event 4	(5) Event 5	(6) Event 6	(7) Event 7
Treatment	-0.287*	-0.312*	-0.370*	-0.386*	-0.409*	-0.285	-0.269
	(0.102)	(0.118)	(0.128)	(0.139)	(0.149)	(0.162)	(0.169)
Age	0.013	0.009	0.007	0.020	0.026	0.027	0.014
	(0.010)	(0.013)	(0.016)	(0.018)	(0.020)	(0.022)	(0.023)
Age^2	0.000	0.000	0.000	0.000	0.000	0.000	0.000
	(0.000)	(0.000)	(0.000)	(0.000)	(0.000)	(0.000)	(0.000)
Female	0.012	0.005	0.007	0.000	-0.032	-0.012	-0.018
	(0.064)	(0.082)	(0.098)	(0.118)	(0.131)	(0.143)	(0.150)
Non-German	0.136	0.144	0.342	0.535	0.571	0.660	0.961*
	(0.191)	(0.211)	(0.234)	(0.256)	(0.272)	(0.289)	(0.304)
n	31,010	31,090	31,162	31,275	31,355	31,452	31,516

Estimates are expressed in terms of percentage points. Standard errors (clustered by address clusters) in parentheses. + $p < 0.1$, * $p < 0.05$, ** $p < 0.01$, *** $p < 0.001$.

© 2025 Ternovski J et al. *JAMA Network Open*

Table 35: Spillover treatment effects on primary vaccinations for each vaccination event (Cumulative). Covariate-adjusted OLS regression.

	(1)<br>Event 1	(2)<br>Event 2	(3)<br>Event 3	(4)<br>Event 4	(5)<br>Event 5	(6)<br>Event 6	(7)<br>Event 7
Treatment	-0.116	-0.092	-0.063	-0.037	-0.035	-0.014	0.021
	(0.093)	(0.096)	(0.103)	(0.109)	(0.115)	(0.121)	(0.124)
Age	-0.003	-0.010	-0.016	-0.010	-0.008	-0.013	-0.026
	(0.006)	(0.008)	(0.011)	(0.013)	(0.014)	(0.015)	(0.016)
Age^2	0.000	0.000	0.000	0.000	0.000	0.000	0.000
	(0.000)	(0.000)	(0.000)	(0.000)	(0.000)	(0.000)	(0.000)
Female	-0.053	-0.048	-0.075	-0.133	-0.174	-0.178	-0.228
	(0.045)	(0.053)	(0.066)	(0.078)	(0.089)	(0.095)	(0.099)
Non-German	0.244	0.343	0.597*	0.856**	0.950***	1.119***	1.444***
	(0.188)	(0.197)	(0.219)	(0.235)	(0.246)	(0.257)	(0.269)
n	30,956	30,979	31,019	31,069	31,105	31,142	31,163

Estimates are expressed in terms of percentage points. Standard errors (clustered by address clusters) in parentheses. + p < 0.1, * p < 0.05, ** p < 0.01, *** p < 0.001.

© 2025 Ternovski J et al. *JAMA Network Open*

Table 36: Spillover treatment effects on booster vaccinations for each vaccination event (Cumulative). Covariate-adjusted OLS regression.

	(1)<br>Event 1	(2)<br>Event 2	(3)<br>Event 3	(4)<br>Event 4	(5)<br>Event 5	(6)<br>Event 6	(7)<br>Event 7
Treatment	-0.172***	-0.221**	-0.311***	-0.354***	-0.378***	-0.274*	-0.295*
	(0.044)	(0.067)	(0.075)	(0.089)	(0.098)	(0.110)	(0.118)
Age	0.015	0.019	0.023	0.031+	0.035+	0.041+	0.041+
	(0.008)	(0.010)	(0.012)	(0.013)	(0.014)	(0.016)	(0.017)
Age^2	0.000	0.000	0.000	0.000	0.000	0.000	0.000
	(0.000)	(0.000)	(0.000)	(0.000)	(0.000)	(0.000)	(0.000)
Female	0.065	0.053	0.082	0.134	0.142	0.165	0.208
	(0.046)	(0.064)	(0.073)	(0.089)	(0.098)	(0.108)	(0.115)
Non-German	-0.107*	-0.199*	-0.253*	-0.318*	-0.374**	-0.457**	-0.478**
	(0.036)	(0.073)	(0.084)	(0.105)	(0.118)	(0.134)	(0.142)
n	30,960	31,017	31,049	31,112	31,156	31,216	31,259

Estimates are expressed in terms of percentage points. Standard errors (clustered by address clusters) in parentheses. + $p < 0.1$, * $p < 0.05$, ** $p < 0.01$, *** $p < 0.001$.

© 2025 Ternovski J et al. *JAMA Network Open*

Table 37: Overall treatment effects for each vaccination event (Cumulative). Covariate-adjusted OLS regression.

	(1)<br>Event 1	(2)<br>Event 2	(3)<br>Event 3	(4)<br>Event 4	(5)<br>Event 5	(6)<br>Event 6	(7)<br>Event 7
Treatment	-0.232*	-0.261*	-0.312*	-0.320*	-0.342*	-0.248	-0.263
	(0.086)	(0.101)	(0.111)	(0.123)	(0.131)	(0.142)	(0.148)
Age	0.014	0.014	0.010	0.021	0.025	0.026	0.017
	(0.009)	(0.012)	(0.014)	(0.016)	(0.018)	(0.019)	(0.021)
Age^2	0.000	0.000	0.000	0.000	0.000	0.000	0.000
	(0.000)	(0.000)	(0.000)	(0.000)	(0.000)	(0.000)	(0.000)
Female	0.060	0.019	0.084	0.052	0.023	0.038	0.045
	(0.056)	(0.070)	(0.084)	(0.100)	(0.109)	(0.118)	(0.124)
Non-German	0.124	0.128	0.316	0.462	0.525	0.670+	1.031**
	(0.173)	(0.192)	(0.211)	(0.231)	(0.246)	(0.263)	(0.278)
n	40,891	40,997	41,098	41,242	41,341	41,462	41,548

Estimates are expressed in terms of percentage points. Standard errors (clustered by address clusters) in parentheses. + $p < 0.1$, * $p < 0.05$, ** $p < 0.01$, *** $p < 0.001$.

© 2025 Ternovski J et al. *JAMA Network Open*

Table 38: Overall treatment effects on primary vaccinations for each vaccination event (Cumulative). Covariate-adjusted OLS regression.

	(1) Event 1	(2) Event 2	(3) Event 3	(4) Event 4	(5) Event 5	(6) Event 6	(7) Event 7
Treatment	-0.097	-0.061	-0.033	-0.003	-0.002	0.016	0.032
	(0.075)	(0.079)	(0.085)	(0.090)	(0.096)	(0.100)	(0.103)
Age	-0.004	-0.009	-0.015	-0.010	-0.010	-0.016	-0.026
	(0.005)	(0.007)	(0.009)	(0.011)	(0.012)	(0.013)	(0.014)
Age^2	0.000	0.000	0.000	0.000	0.000	0.000	0.000
	(0.000)	(0.000)	(0.000)	(0.000)	(0.000)	(0.000)	(0.000)
Female	-0.033	-0.067	-0.068	-0.120	-0.142	-0.157	-0.184
	(0.036)	(0.044)	(0.055)	(0.064)	(0.071)	(0.076)	(0.079)
Non-German	0.239	0.326	0.575*	0.798***	0.919***	1.109***	1.460***
	(0.169)	(0.179)	(0.197)	(0.211)	(0.223)	(0.233)	(0.246)
n	40,808	40,839	40,889	40,943	40,989	41,030	41,059

Estimates are expressed in terms of percentage points. Standard errors (clustered by address clusters) in parentheses. + $p < 0.1$, * $p < 0.05$, ** $p < 0.01$, *** $p < 0.001$.

Table 39: Overall treatment effects on booster vaccinations for each vaccination event (Cumulative). Covariate-adjusted OLS regression.

	(1) Event 1	(2) Event 2	(3) Event 3	(4) Event 4	(5) Event 5	(6) Event 6	(7) Event 7
Treatment	-0.135**	-0.201**	-0.283***	-0.320***	-0.344***	-0.265*	-0.299*
	(0.042)	(0.062)	(0.071)	(0.084)	(0.091)	(0.101)	(0.108)
Age	0.019*	0.024*	0.025*	0.031*	0.036*	0.043*	0.043*
	(0.007)	(0.009)	(0.010)	(0.012)	(0.013)	(0.015)	(0.016)
Age^2	0.000	0.000	0.000	0.000	0.000	0.000	0.000
	(0.000)	(0.000)	(0.000)	(0.000)	(0.000)	(0.000)	(0.000)
Female	0.092	0.085	0.155	0.176	0.168	0.196	0.231
	(0.043)	(0.055)	(0.064)	(0.077)	(0.083)	(0.091)	(0.097)
Non-	-0.115**	-0.199**	-0.257**	-0.333**	-0.392**	-0.438**	-0.420**
German	(0.034)	(0.067)	(0.076)	(0.096)	(0.106)	(0.123)	(0.132)
n	40,835	40,910	40,961	41,051	41,104	41,184	41,241

Estimates are expressed in terms of percentage points. Standard errors (clustered by address clusters) in parentheses. + p < 0.1, * p < 0.05, ** p < 0.01, *** p < 0.001.

1.7 Balance Tests

Table 40: Balance tests. OLS regression estimates of the effects of the socio-demographic variables on treatment arm assignment.

	(1) Direct effect	(2) Spillover effect	(3) Overall effect
Age	0.000	0.000	0.000
	(0.000)	(0.000)	(0.000)
Female	0.003	-0.009+	-0.006+
	(0.013)	(0.005)	(0.003)
Non-German	0.019	-0.021	-0.015
	(0.024)	(0.014)	(0.013)
Cluster size	-0.056	-0.047	-0.048
	(0.060)	(0.055)	(0.055)
n	10,032	31,516	41,548

Standard errors (clustered by address clusters) in parentheses. + p < 0.1, * p < 0.05, ** p < 0.01, *** p < 0.001.

eAppendix 7. Statistical Power and Sensitivity Analysis

We estimated power as part of our pre-registration plan (see below, Section 10 – Pre-registered Analysis Plan, Sample Size). For more realistic *post hoc* estimates of power, we used the observed Intraclass Correlation Coefficient (ICC) in the control group and the sample sizes in our data in order to calculate the minimum detectable effect (MDE) for each of our estimators (using the power command in Stata 15.1). The results are presented in Table 41.

Table 41: Probability of observing the treatment effect.

Effect Type	MDE	Power
Direct effect (information uptake)	0.010	80%
Direct effect (vaccinations)	0.011	83%
Spillover effect (information uptake)	0.005	79%
Spillover effect (vaccinations)	0.006	82%
Overall effect (information uptake)	0.005	80%
Overall effect (vaccinations)	0.006	83%

As recommended in (Lakens 2022), we also include sensitivity power analyses for all the effect types. As seen in Figures 4-15, our study is very-well powered to detect even very small effects. While we use Cohen's *d* to illustrate effect sizes in a more standardized fashion, we caution against using default interpretations of Cohen's *d* from outside of a public policy context. While a *d* of 0.1 is often considered trivial in the fields of psychology and education (e.g., Lakens, Scheel, Isager 2018), a *d* of 0.1 can be highly consequential in a public health context (e.g., Lakens 2013). The effect sizes we observed in our data are depicted in red and we note the effect size and power for all statistically significant effects at p=0.1.

© 2025 Ternovski J et al. *JAMA Network Open*

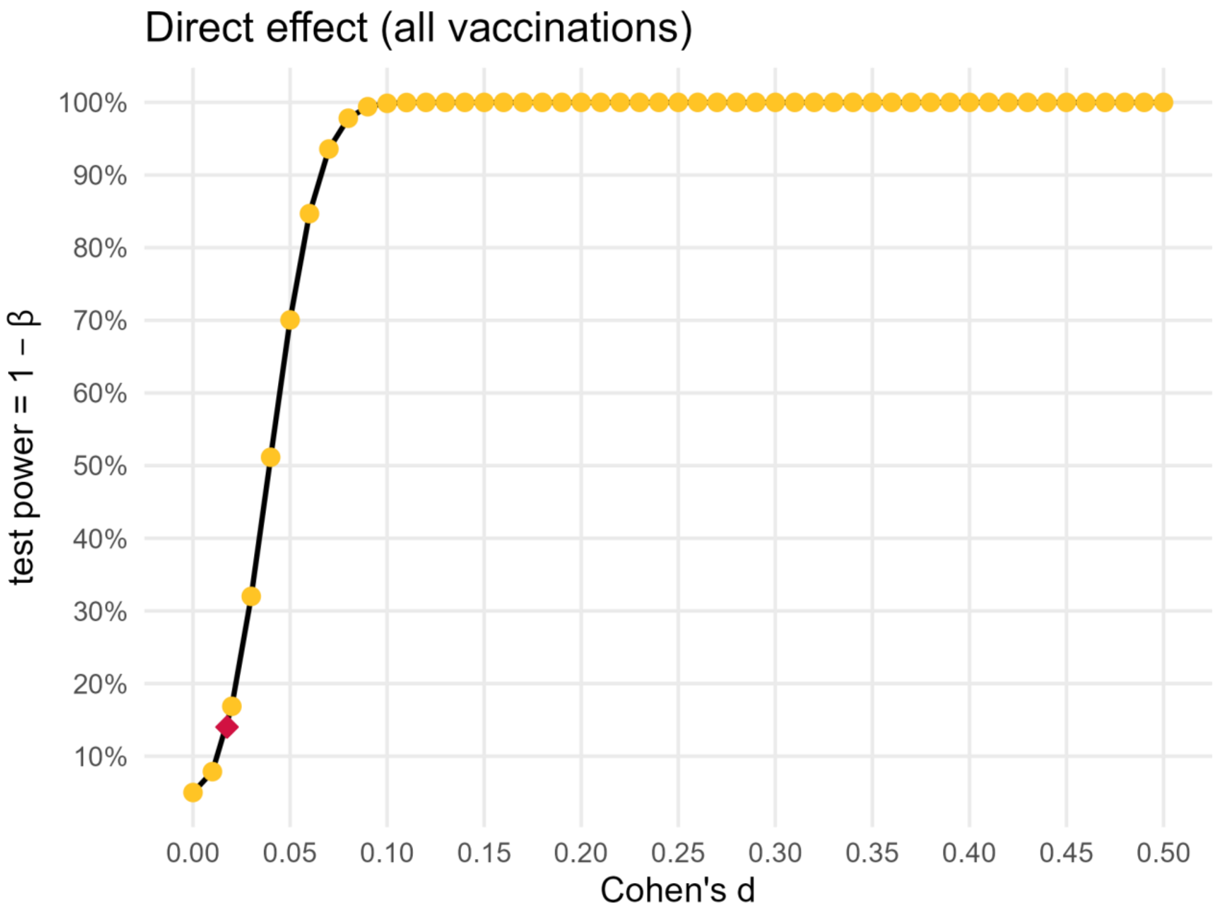

Figure 4: Sensitivity analysis of the direct effect on all vaccinations.

© 2025 Ternovski J et al. *JAMA Network Open*

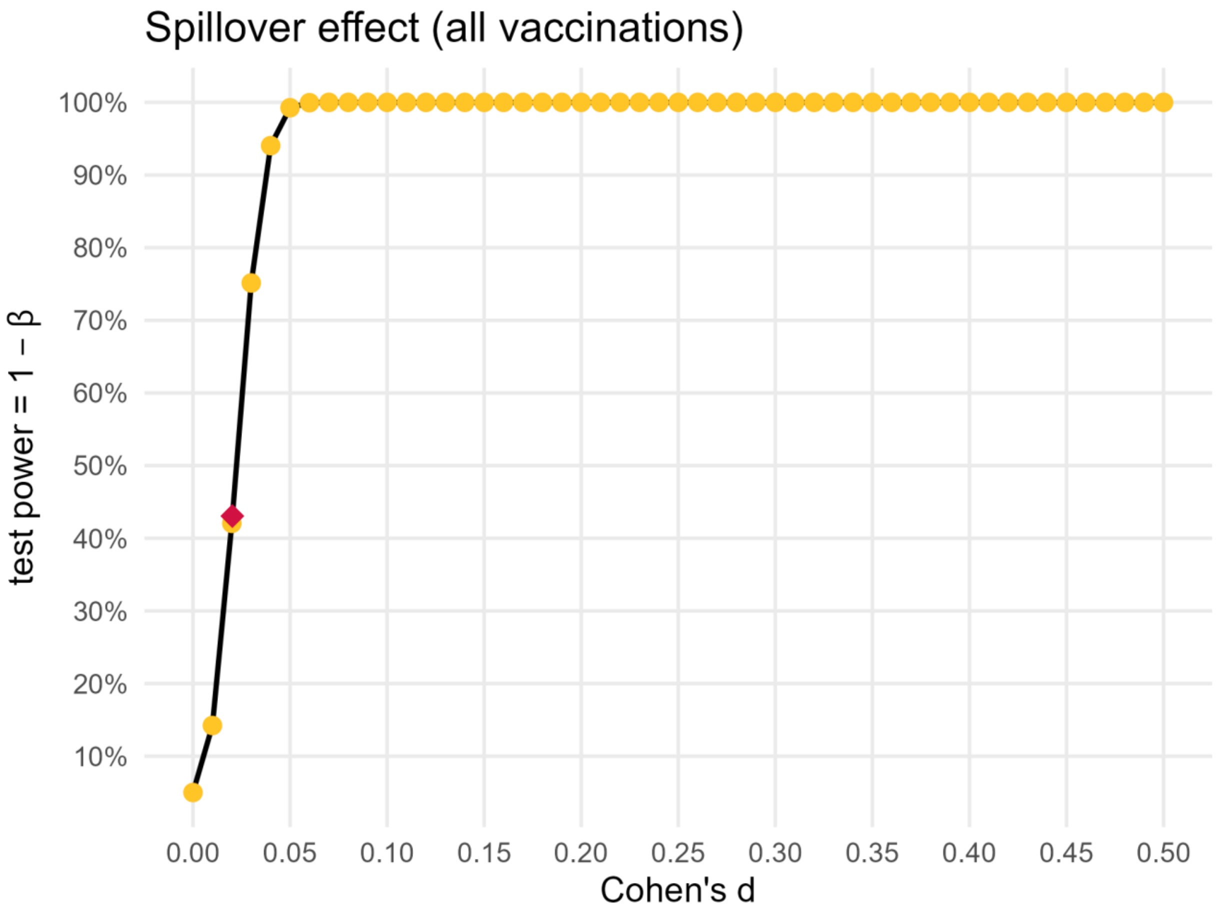

Figure 5: Sensitivity analysis of the spillover effect on all vaccinations.

© 2025 Ternovski J et al. *JAMA Network Open*

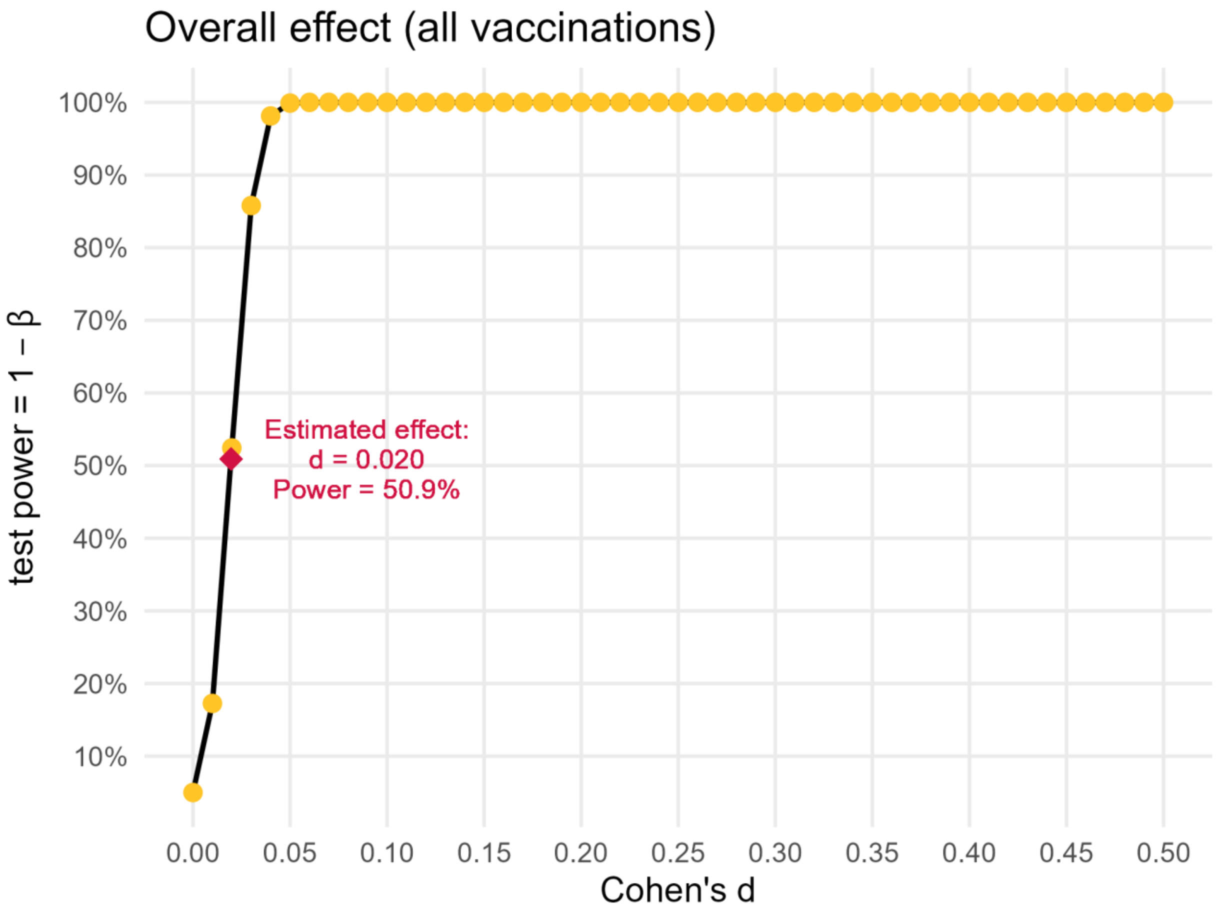

Figure 6: Sensitivity analysis of the overall effect on all vaccinations.

© 2025 Ternovski J et al. *JAMA Network Open*

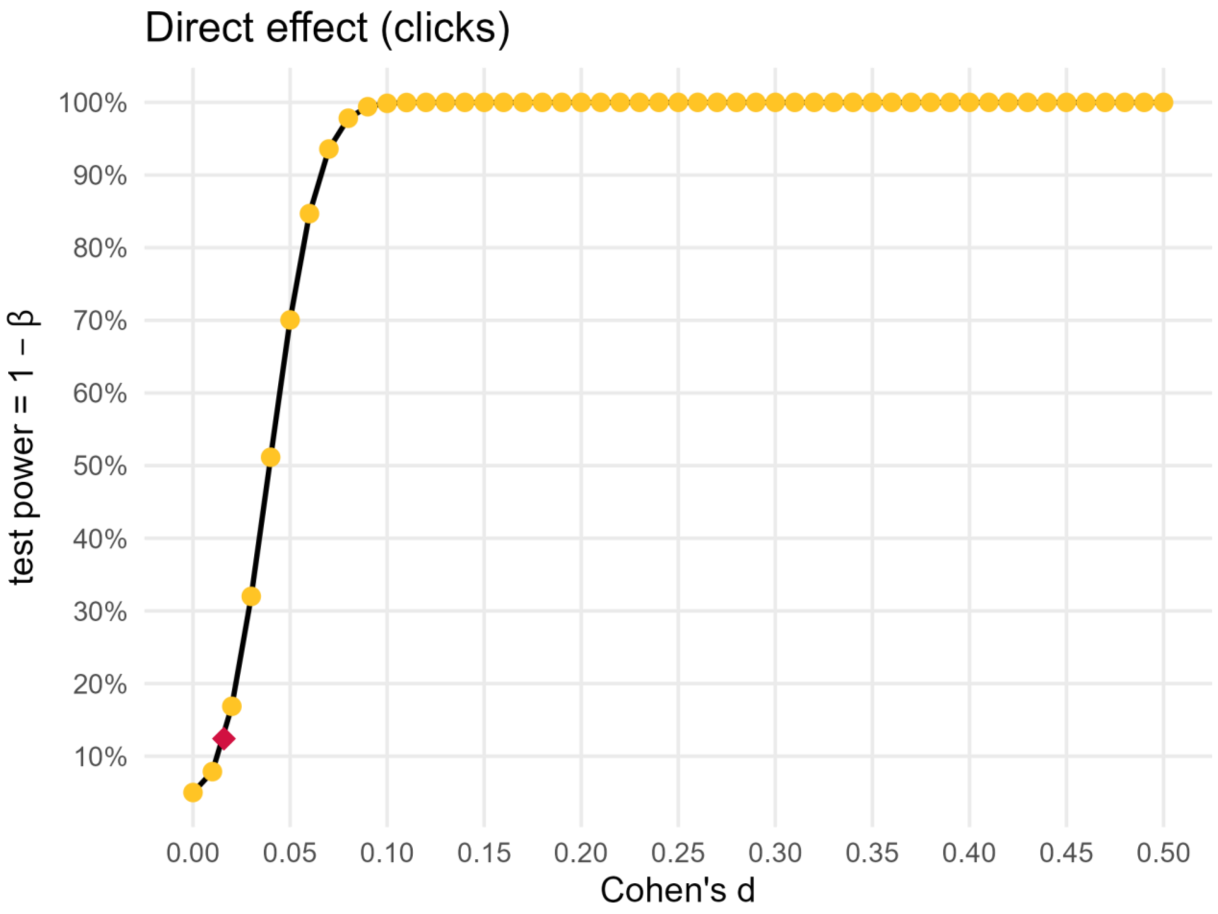

Figure 7: Sensitivity analysis of the direct effect on all information uptake (clicks).

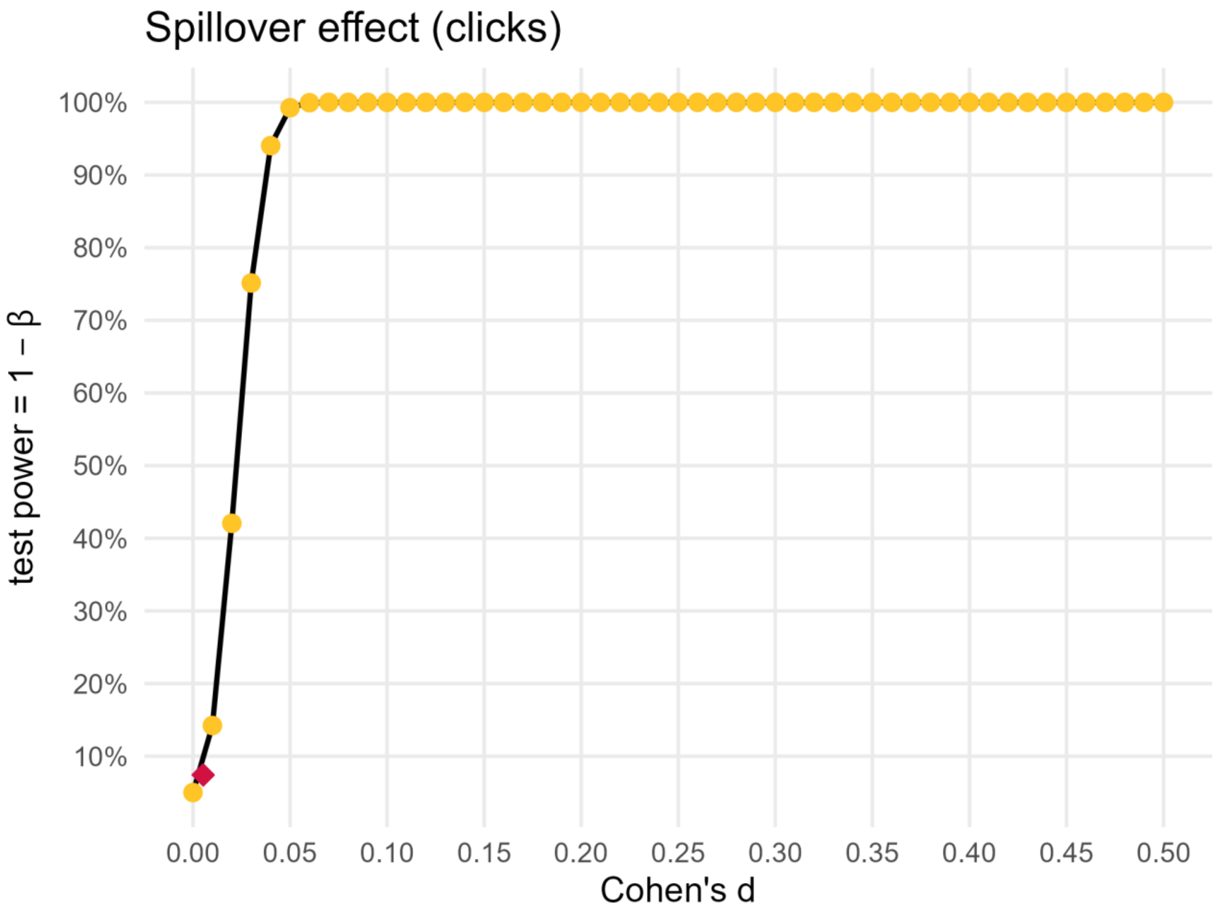

Figure 8: Sensitivity analysis of the spillover effect on all information uptake (clicks).

© 2025 Ternovski J et al. *JAMA Network Open*

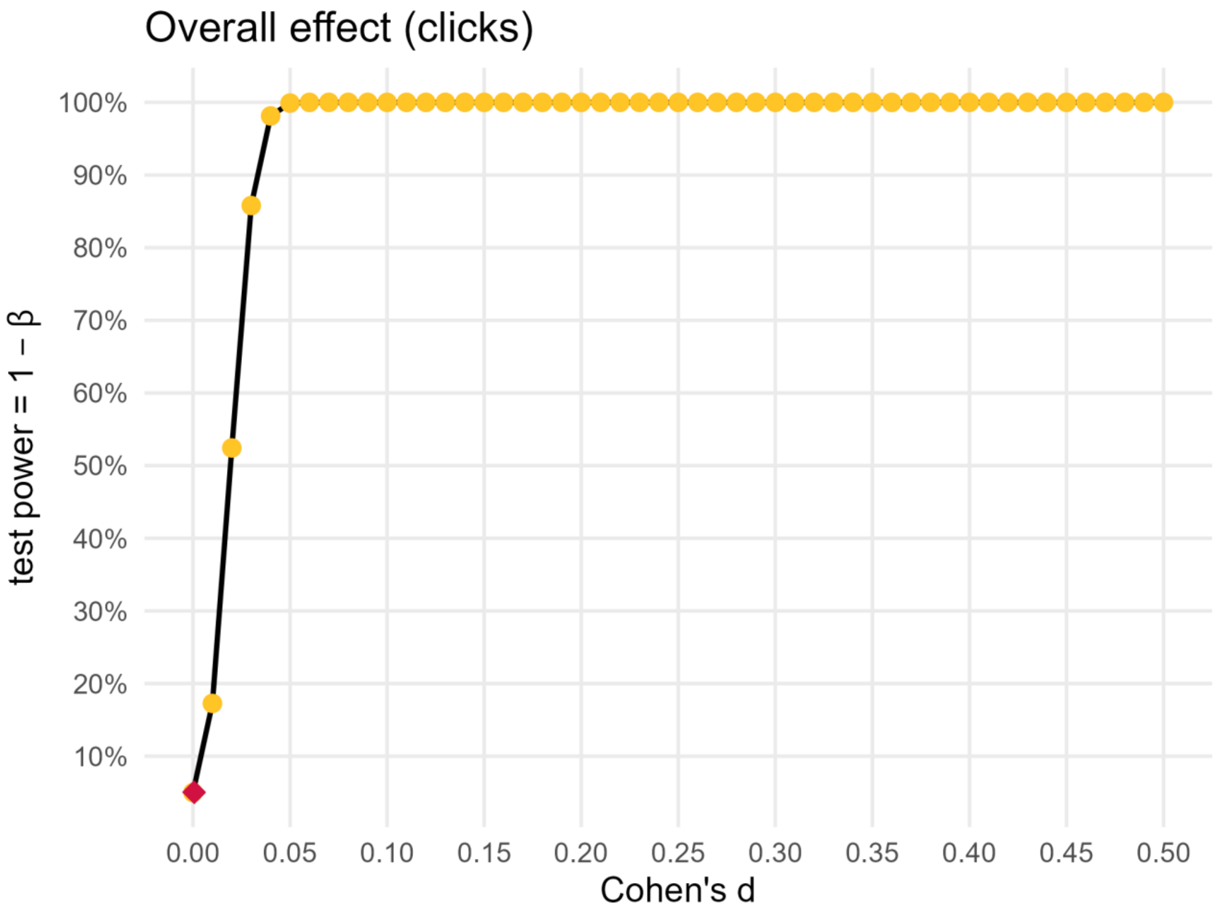

Figure 9: Sensitivity analysis of the overall effect on information uptake (clicks).

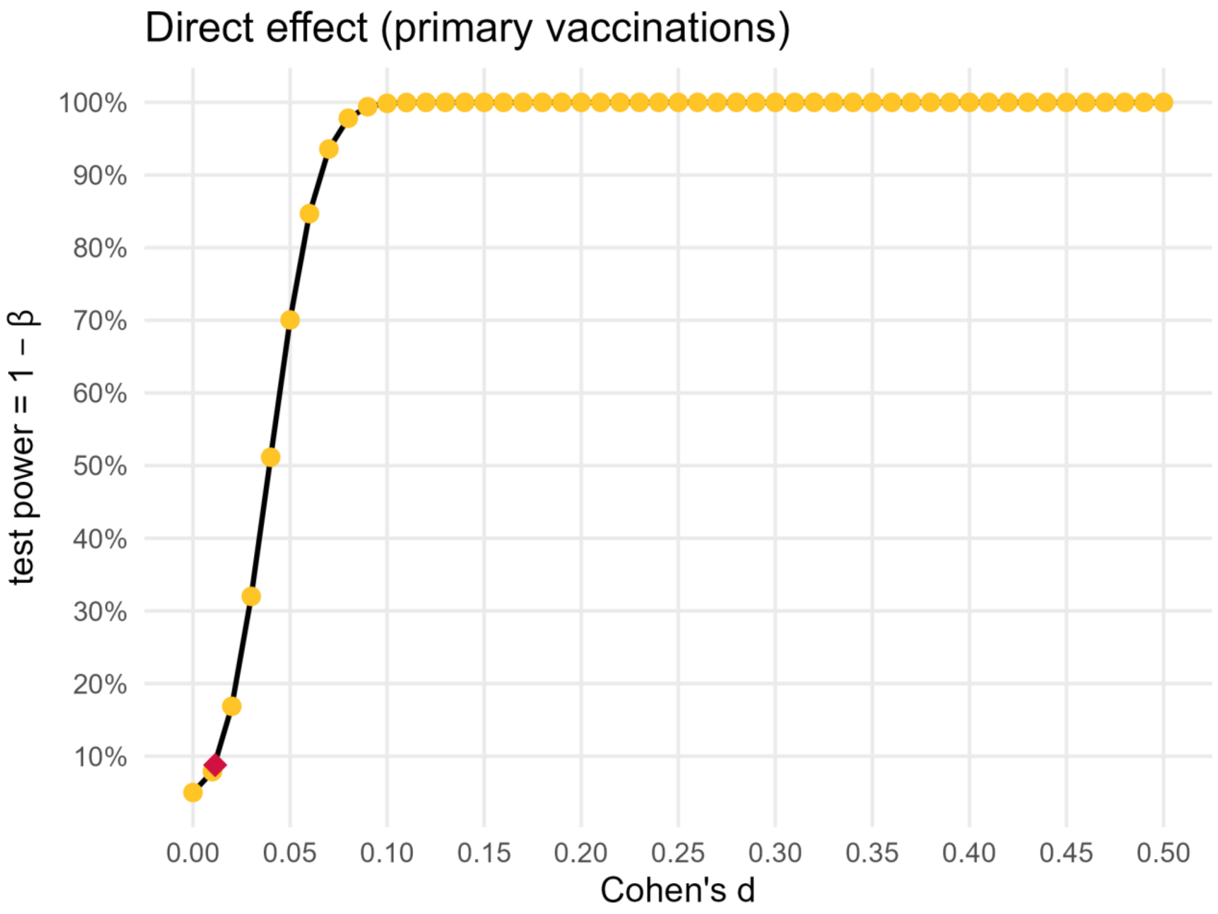

Figure 10: Sensitivity analysis of the direct effect on primary vaccinations.

© 2025 Ternovski J et al. *JAMA Network Open*

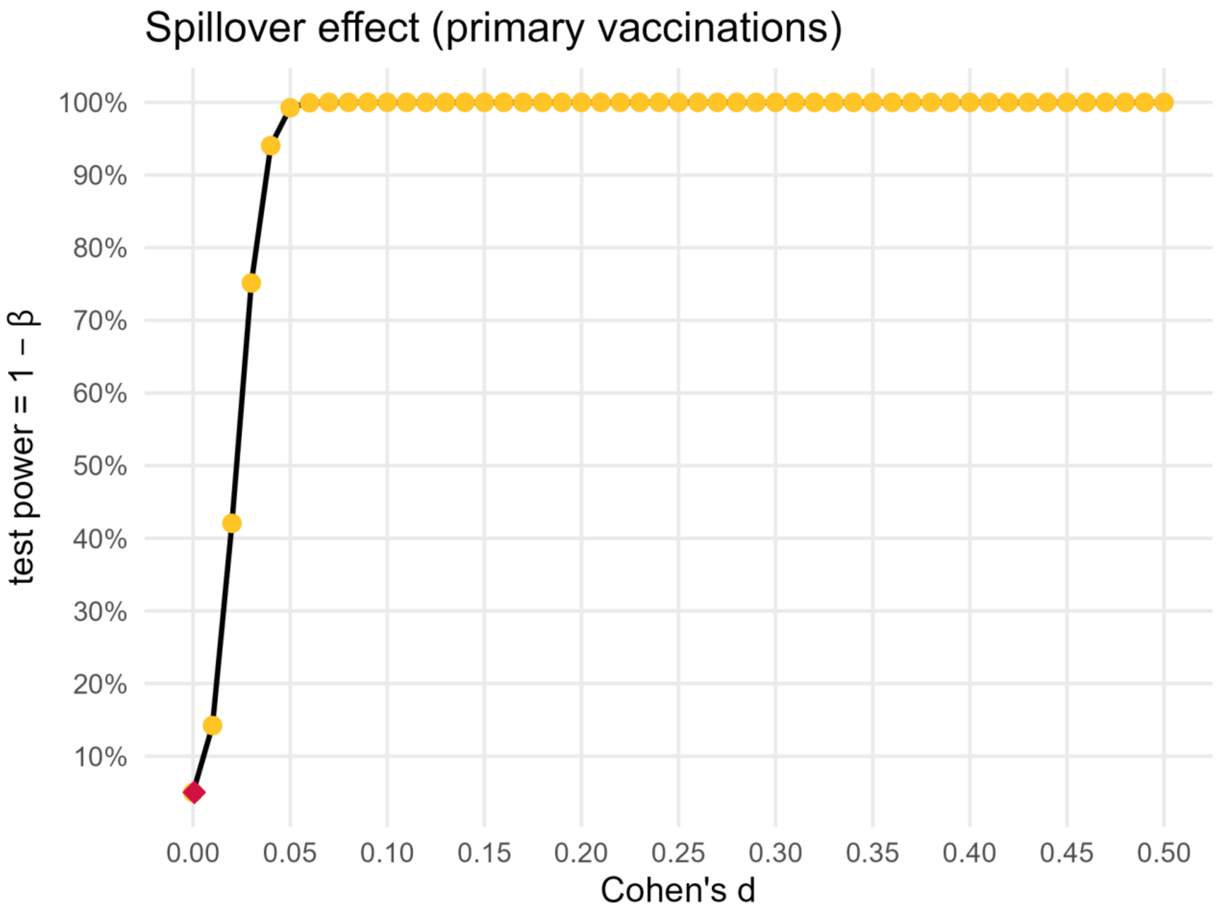

Figure 11: Sensitivity analysis of the spillover effect on primary vaccinations.

© 2025 Ternovski J et al. *JAMA Network Open*

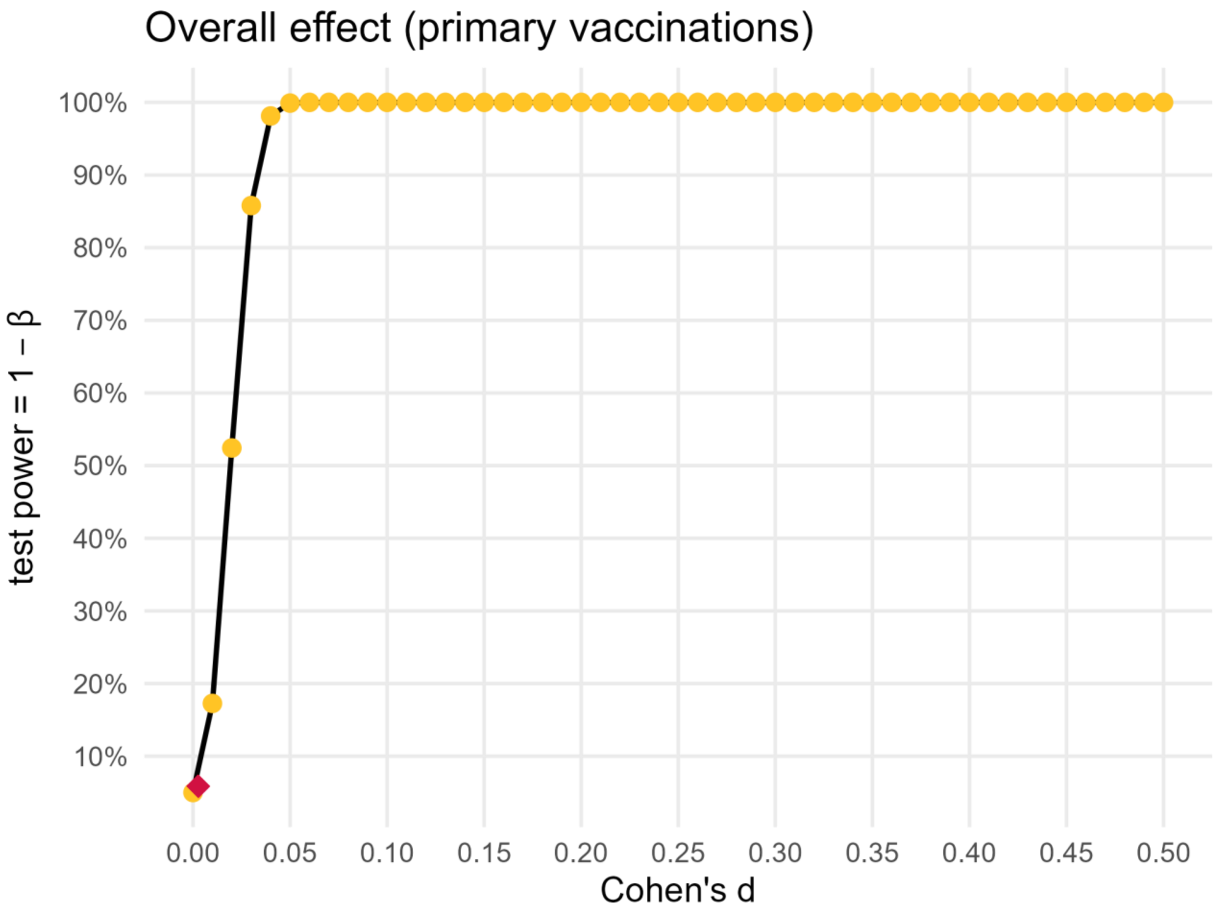

Figure 12: Sensitivity analysis of the overall effect on primary vaccinations.

© 2025 Ternovski J et al. *JAMA Network Open*

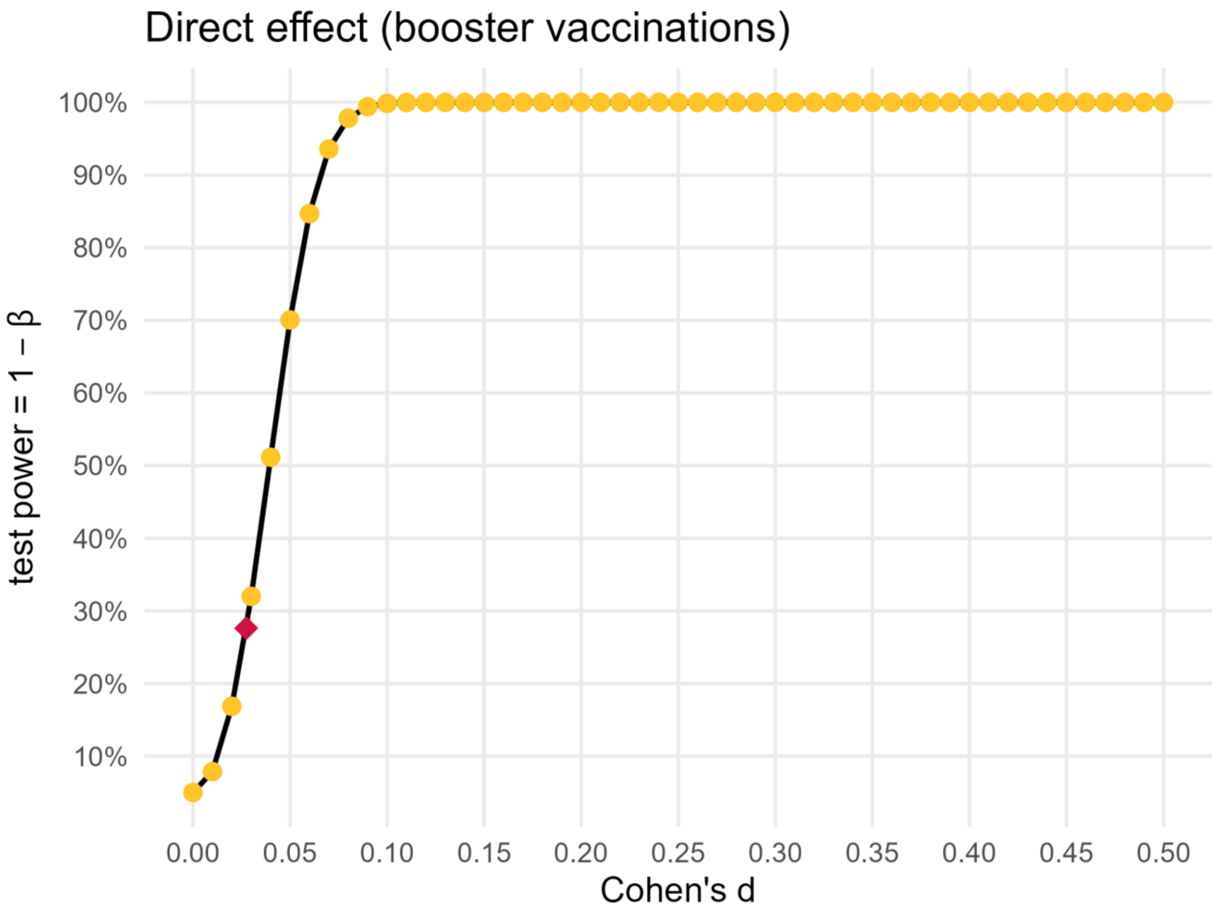

Figure 13: Sensitivity analysis of the direct effect on booster vaccinations.

© 2025 Ternovski J et al. *JAMA Network Open*

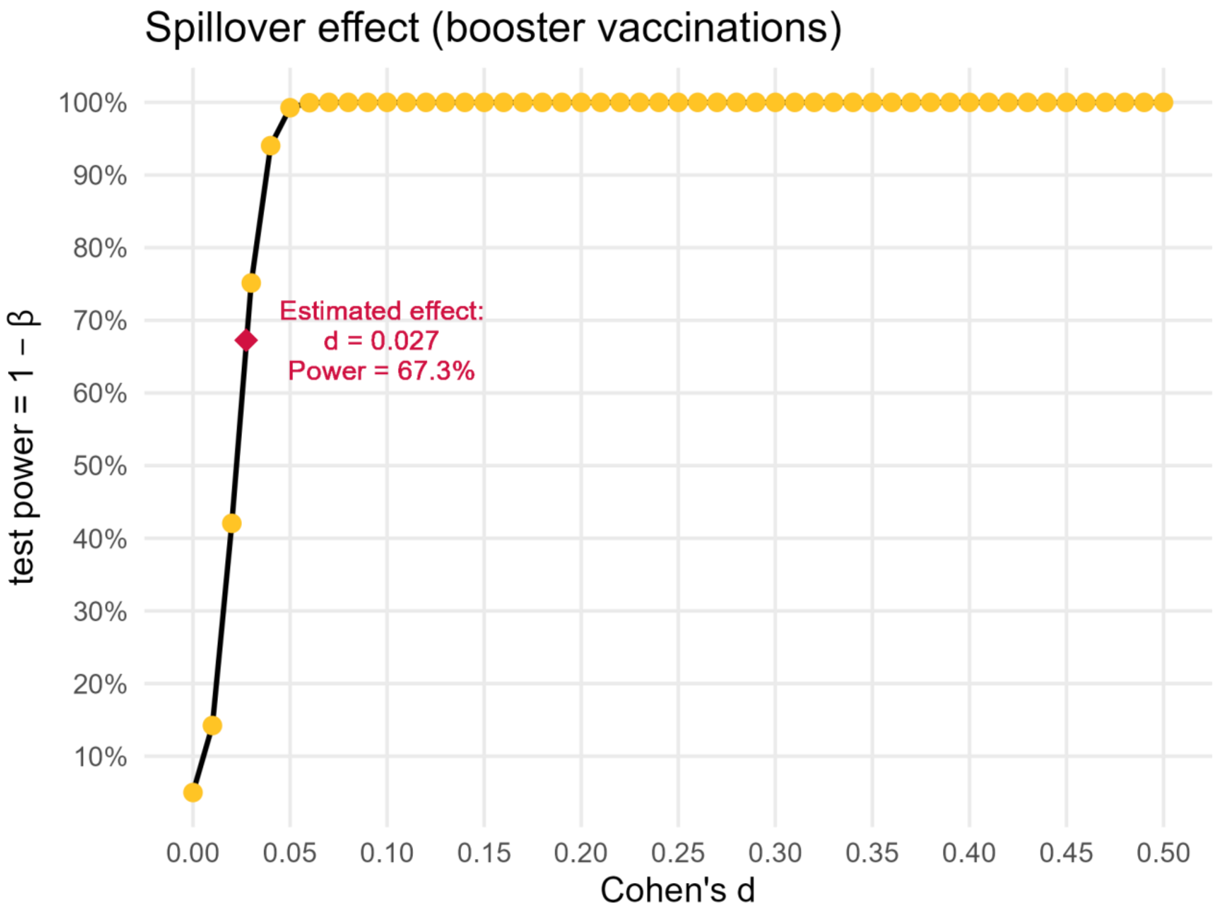

Figure 14: Sensitivity analysis of the spillover effect on booster vaccinations.

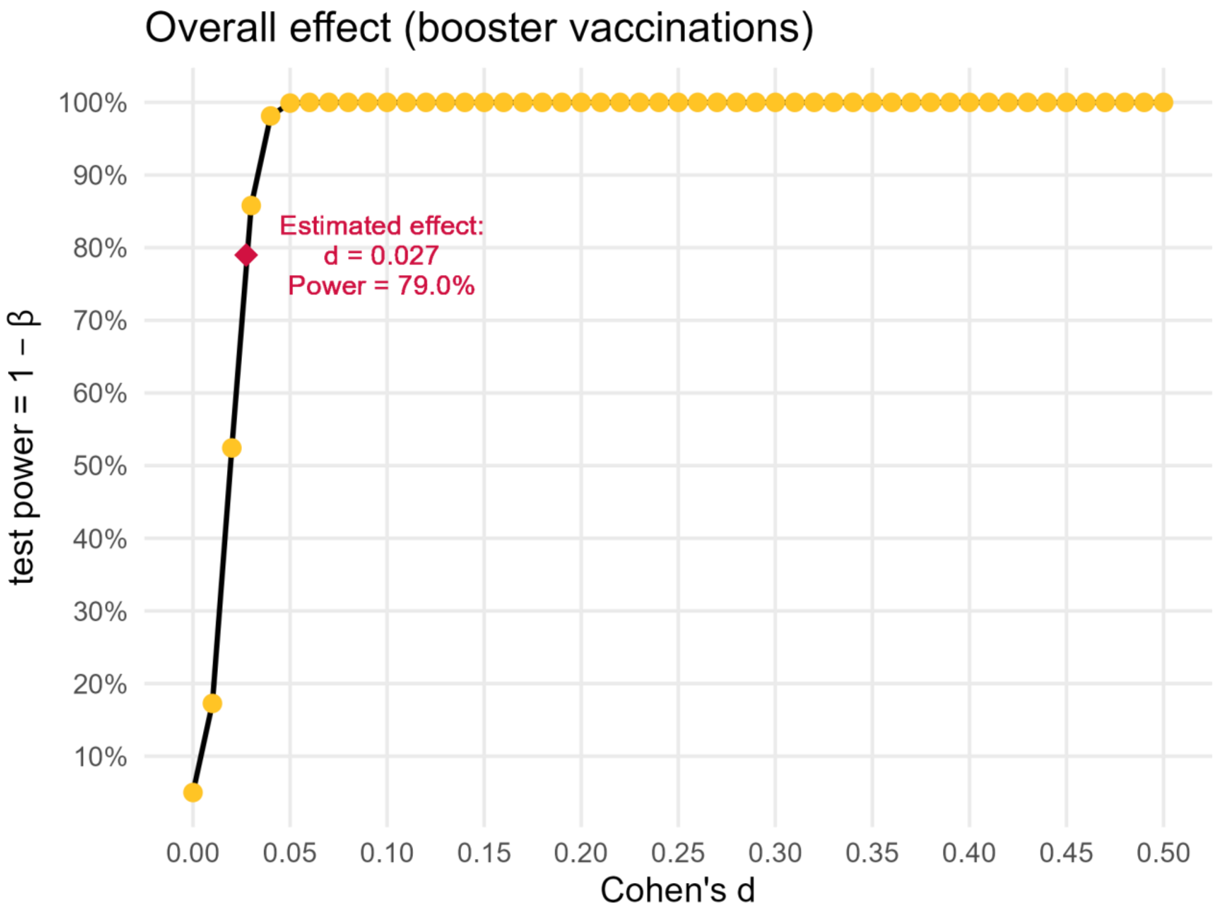

Figure 15: Sensitivity analysis of the overall effect on booster vaccinations.

To supplement this power analysis with a more precise formulation of the magnitude of effects we would be able to observe with our data, we applied randomization inference to the observed outcome data. For every treatment effect we present in Figure 3 of the article, we randomly permute the treatment assignment vector (in the same way that random assignment was initially conducted [i.e., block and cluster random assignment]) for 1,000 iterations and calculate the synthetic "null" treatment effect. This results in a synthetic null distribution of observed treatment effects, which yields a standard randomization inference p-value (as seen in Section 6.3). We then iteratively incorporate a treatment effect in the synthetic distribution by increasing the probability of the synthetic outcome variable for synthetic treatment participants by 0.1 percentage points. (Individuals who are assigned to the synthetic control keep their randomly permuted outcomes.) We then calculate the treatment estimator of interest (either the direct, overall, or spillover effect) in this synthetic data. We repeat for 1,000 iterations to generate a synthetic distribution where the true effect is known. We compare the observed "true" treatment effect to this synthetic distribution of a known synthetic effect. We repeat until the probability of observing the true treatment effect in our observed data is less than .05 in the synthetic distribution. The results for all of our estimators are presented below in Table 42.

Table 42: Probability of observing the treatment effect.

Effect Type	If true effect size is at least	How unlikely is it to appear as observed in our data?
Direct effect (information uptake)	0.010	3.5%
Direct effect (vaccinations)	0.003	4.6%
Spillover effect (vaccinations)	0.003	4.6%
Overall effect (information uptake)	0.015 (with a constant spillover effect of 0.0005)	2.9%
Overall effect (vaccinations)	0.003 (with a constant spillover effect of 0.002)	1.6%

eAppendix 8. Data and Code Availability

To avoid inadvertently disclosing participants' sensitive health and socio-demographic information, we do not provide the full data publicly. However, we provide a reduced dataset that includes the outcome variables (i.e, vaccination and information uptake), treatment condition (i.e., treatment arm and cluster representative status), and a cluster ID that represents the individual's household.

Additionally, we salted the data to increase anonymity. Specifically, we randomly selected three vaccinated and three unvaccinated participants in the treatment and control arms, respectively, and inverted their vaccination uptake record. We did this twice: once for clusters with 25 or more cohabitants and once for clusters with less than 25 cohabitants. This procedure results in 24 inverted outcome measures. This way, interested researchers can reproduce our main results without exposing participants to potential de-anonymization. While the replication data excludes covariates and all our reported results include covariate adjustment, we note that covariate adjustment does not meaningfully alter any results. Furthermore, we provide the full R analysis code and its output in an additional document.

© 2025 Ternovski J et al. *JAMA Network Open*

eAppendix 9. Ethics

The study was approved by Zeppelin University's ethics committee on October 29th, 2022. The committee waived individuals' informed consent as it relates to 1) being included in the study and 2) the collection of data as to whether or not an individual was observed consenting to be vaccinated at one of the vaccination events. In strict compliance with the guidelines of the German public health authorities, all vaccination events included a mandatory consultation by a doctor prior to vaccination and the participant's signed confirmation of consent to get vaccinated. The Zeppelin University ethics committee waived participants' informed consent as it relates to being included in the study and the collection of data as to whether or not a participant was observed consenting to be vaccinated at the public event.

© 2025 Ternovski J et al. *JAMA Network Open*

eAppendix 10. Pre-registered Analysis Plan

We pre-registered the data collection, analysis, and hypotheses at the Open Science Framework (https://osf.io/2trmb/?view_only=4d4cd0da2f86424a812c6b76282c78e5) and at ISRCTN (study record #59503725). The Open Science Framework pre-registered analysis plan is reproduced here in full, for convenience.

1.8 Study Information

1.8.1 Hypotheses

H1-1 (direct or "total" effect #1): Offering a set of monetary incentives increases the number of unique website visits (on a municipal webpage with information on COVID-19 vaccination) among treated subjects in treatment clusters compared to equivalent subjects in control clusters (i.e., in each control cluster, the person who would have received the treatment letter had they been assigned to a treatment cluster).

H1-2 (direct or "total" effect #2): Offering a set of monetary incentives increases COVID-19 vaccinations (tracked at seven city vaccinations events) among treated subjects in treatment clusters compared to equivalent subjects in control clusters (i.e., in each control cluster, the person who would have received the treatment letter had they been assigned to a treatment cluster).

H2 (spillover effect): Offering a set of monetary incentives increases COVID-19 vaccinations among untreated individuals in treatment clusters compared to untreated subjects in control clusters (minus the individual who would have received a treatment letter in the control cluster if they were assigned to a treatment cluster).

H3-1 (overall effect #1): Offering a set of monetary incentives increases unique website visits among treated and untreated individuals in treatment clusters compared to untreated subjects in control clusters.

H3-2 (overall effect #2): Offering a set of monetary incentives increases COVID-19 vaccinations among treated and untreated individuals in treatment

1.9 Design Plan

1.9.1 Study Type

Experiment - A researcher randomly assigns treatments to study subjects, this includes field or lab experiments. This is also known as an intervention experiment and includes randomized controlled trials.

1.9.2 Blinding

No blinding is involved in this study.

1.10 Study Design

The design is a blocked, two-stage (individual and household) randomized controlled field experiment.

1.10.1 Randomization

The study will be conducted in a medium-sized city in Germany. Treatment assignment follows a two-stage randomization process. In the first stage, all housing addresses within the city will be randomized into two groups (i.e., intervention group and control group), blocked by household size. In the second stage, one resident of each address in both groups will be randomly selected (a "cluster representative"). The cluster representatives in the intervention group only will be sent the intervention letter. All remaining residents will be sent the control letter.

The randomization will be conducted in R (version 4.1.1) using the function "sample" with the seed set to 2021.

1.11 Sampling Plan

1.11.1 Existing Data

Registration prior to creation of data.

1.11.2 Data collection procedures

The study will be conducted in the city of Ravensburg, Germany. All residents of the city (age 18 and older) will be subject to the trial (as per official residency records). The only notable exception is that the study excludes the inmates of a local prison (n=257) and the second largest cluster of the city (n=75). Treatment assignment follows a two-stage randomization process. In the first stage, all housing addresses within the city will be randomized into two groups (i.e., intervention group and control group), blocked by household size. In the second stage, one resident of each address in both groups will be randomly selected (a "cluster representative"). The cluster representatives in the intervention group only will be sent the intervention letter. All remaining residents will be sent the control letter.

The treatment group will receive a letter from the mayor of the city inviting them to get a COVID-19 vaccination at one of seven public vaccination events. The letter offers two financial incentives. If the recipient is not already vaccinated and gets vaccinated at the vaccination events, they will receive 20 Euros (in the form of a shopping voucher). Additionally, another 20 Euro incentive is offered if more than 900 city residents get vaccinated at one of the seven vaccination events. If the recipient is already vaccinated, they can still get the second incentive if they give the treatment letter to someone else and that individual brings it to one of the seven vaccination events, gets vaccinated, and more than 900 city residents get vaccinated during one of the seven vaccination events.

The control group will get the same letter as the treatment group but without the offer of monetary incentives.

1.12 Sample Size

The sample consists of all residents of the city (n = 41,549) clustered into 10,033 addresses. The only notable exception is that the study excludes the inmates of a local prison (n=257) and the second largest cluster of the city (n = 75).

1.12.1 Sample size rationale

The sample size is determined by the population of the city. Based on simulation-based power calculations, we are able to detect a minimal direct or "total" effect of 2.6 percentage points, a spillover effect of 1.5 percentage points, and an overall effect of 1.3 percentage points with approximately 80% statistical power (alpha = 0.05). This calculation is based on the assumption that 20% of the population of the city is not eligible for a primary vaccine or booster and that eligibility is uniformly distributed across individuals. We project that up to 20% of the control group will get vaccinated at these events.

1.12.2 Stopping rule

The effect of the treatment will be assessed by measuring how many people get vaccinated at the seven public vaccination events on 2021-11-13, 2021-11-19, 2021-11-20, 2021-11-26, 2021-11-27, 2021-12-10, and 2021-12-11.

1.13 Variables

1.13.1 Manipulated variables

The treatment group will receive a letter from the mayor of the city inviting them to get a COVID-19 vaccination at one of seven public vaccination events. The letter offers two financial incentives. If the recipient is not already vaccinated and gets vaccinated at the vaccination events, they will receive 20 Euros (in the form of a shopping voucher). Additionally, another 20 Euro incentive is offered if more than 900 city residents get vaccinated at one of the seven vaccination events. If the recipient is already vaccinated, they can still get the second incentive if they give the treatment letter to someone else and that individual brings it to one of the seven vaccination events, gets vaccinated, and more than 900 city residents get vaccinated during one of the seven vaccination events.

The control group will get the same letter as the treatment group but without the offer of monetary incentives

1.13.2 Measured variables

Primary measures:

Information uptake is measured by whether a participant visited the informational website mentioned in the letter (unique website visits). The website is prominently displayed in both treatment and control letters. Each URL and QR-code is unique to the individual recipient.

Vaccination uptake is measured via an on-site record of administered vaccinations during the seven public vaccination events. The records will distinguish between primary vaccines (i.e., first and second doses) and boosters.

1.13.3 Covariate measures

These data will be collected from municipal administrative records and will be used as covariates to increase precision

- Age
- Sex
- Indicator for whether the individual is a non-German national

1.13.4 Additional measures

To determine an individual's close social network, we use address data from municipal administrative records to identify shared addresses and generate unique clusters. As an alternative specification, we use shared last name and shared address to generate a secondary cluster id. The treatment letters given to other residents by fully-vaccinated treatment letter recipients will be collected at the public vaccination events to track which subjects were referred to get vaccinated by the treatment-letter recipient.

We will also collect information (self-reports) whether patients are part of a vulnerable high-risk group which the German federal government prioritizes in the vaccine distribution.

1.14 Analysis Plan

1.14.1 Statistical models

We will use OLS regression with a treatment indicator, block fixed effects, and control covariates (consisting of a binary indicator for female, an indicator for non-German nationality, age, and age squared) in all subsequent analyses unless indicated otherwise. To ensure robustness, a secondary analysis will regress outcomes against the treatment indicator without controls.

H1-1 (direct or "total" effect #1): Information uptake regressed against treatment group for only cluster representatives, with robust standard errors.

H1-2 (direct or "total" effect #2): Vaccination uptake regressed against treatment group for only cluster representatives, with robust standard errors.

© 2025 Ternovski J et al. *JAMA Network Open*

H2 (spillover effect): Vaccination uptake regressed against treatment group for all individuals who are not cluster-representatives, with robust clustered standard errors.

H3-1 (overall effect #1): Information uptake regressed against treatment group for all individuals, with robust clustered standard errors.

H3-2 (overall effect #2): Vaccination uptake regressed against treatment group for all individuals, with robust clustered standard errors.

We will use p-values (threshold = 0.05) associated with the treatment indicator coefficient in our regressions.

1.14.2 Data exclusion

Aside from the exclusion of the two largest clusters, since we use block fixed effects, the one subject in a single-person household that was randomly determined to be in a block size of one will be dropped from analysis.

If a person gets multiple shots (e.g., first and second dose), we will count them only once - namely, for their first shot.

1.14.3 Exploratory analysis

For the analysis, all residents living at the same address will be treated as members of the same social network. We will test an alternative operationalization of a social network by considering all residents with the same last name who reside in the same address cluster as members of the same social network.

We will also explore whether the hypothesized effects differ by German vs. non-German nationals.

To better map out social influence, we will also compare which letters were given to individuals living in the same address as the treatment-letter recipient, which were given to individuals in the same address with the same last name, and which were given to individuals at a different address.

Additionally, we will conduct analysis restricted to

1) first doses (excluding second doses and booster shots),
2) first and second doses (excluding booster shots), and
3) boosters (excluding first and second doses).

To explore whether the treatment effects in H1-2, H2, and H3-2 are driven by an increase in booster shots, we will perform the analyses as described in H1-2, H2, and H3-2 but using a multinomial logistic regression with no record of vaccination equal to zero, primary vaccination equal to 1, and booster vaccination equal to 2. A Wald test will be used to determine if the treatment coefficient in terms of primary vaccination is statistically significantly different from that of boosters.

There is a fair chance that in early November, the Standing Committee on Vaccination at the Robert-Koch Institute – Germany's scientific body to provide recommendations for vaccine schedules – may recommend boosters to either the whole population, or some larger subset of the population (e.g., based on age and risk factors). As of 2021-11-02, they recommend boosters only for

people aged 70 or older and people with a high risk of severe consequences of a COVID-19 infection (e.g., immunocompromised patients). The extension of these guidelines may occur in the middle of our collection of outcomes. If this occurs, for the population affected by the announcement, we will also analyze the interaction of treatment assignment with the timing of this announcement (as a binary indicator =0 when outcomes are collected before the announcement and =1 when outcomes are collected after the announcement).

In addition, we will also examine heterogenous treatment effects by single event-sessions (analyzing Friday/Saturday events as a single event-session, which makes 4 event-sessions in total). This means we will estimate the above-described models for hypotheses 1-3 by each event-session separately. We will do so cumulatively (i.e., event-session #1, event-sessions #1 + #2, …, event-sessions #1 + #2 + #3 + #4) and non-cumulatively (i.e., net vaccination rates for each event-session), and correct for multiple testing. The event-session-analysis will depict before and after comparisons of potential public health announcements and regulations by the Standing Committee on Vaccination (STIKO), the Federal Government, and/or the State of Baden Württemberg that occur during the study period.

We will run additional exploratory analyses to include any additional vaccination events that the city communicates in conjunction with the vaccination campaign, but which were not announced in the letters. We compare them to results of the vaccination events announced in the letters.

To have a better understanding of all indirect costs of using monetary incentives to encourage vaccinations, we will also examine whether clusters assigned to treatment (as compared to clusters assigned to control) are more likely to contact the municipal government with feedback and if that feedback is more negative. (For a discussion of reactance to similar pro-social nudges, see Mann 2010). Civil servants managing the hotline about the vaccination program rank how positive the call is on a Likert scale and we also track all email inquiries. We will have the emails coded in a similar way and we will then analyze these outcomes as recommended in Coppock (2019).

Coppock, A. (2019). Avoiding post-treatment bias in audit experiments. Journal of Experimental Political Science, 6(1), 1-4.

Mann, C. B. (2010). Is there backlash to social pressure? A large-scale field experiment on voter mobilization. Political Behavior, 32(3), 387-407.

1.15 Other

This preregistration is an update to a previous one. We published it prior to collecting any data. The update was necessary to add exploratory analyses to capture regulatory interventions by government bodies that might occur during the study period and could affect the effects of the treatment as well as to capture decisions made by the involved city. We, therefore, added the last three paragraphs under "exploratory analysis", and specified that people getting vaccinated multiple times will only be counted once (see "Data exclusion").

eAppendix 11. Additional Analyses

1.16 Regression Table of Impact on Information Uptake

Table 43: Treatment effects on information uptake. OLS regression estimates of the effects of the treatment on information uptake.

	Direct effect		Spillover effect		Overall effect	
	(1) Unadjusted	(2) Adjusted	(3) Unadjusted	(4) Adjusted	(5) Unadjusted	(6) Adjusted
Treatment	0.339	0.337	-0.101	-0.106	0.006	0.000
	(0.339)	(0.339)	(0.152)	(0.152)	(0.138)	(0.138)
Age		0.138*		0.142***		0.140***
		(0.056)		(0.022)		(0.021)
Age²		-0.001**		-0.001***		-0.001***
		(0.001)		(0.000)		(0.000)
Female		-0.168		0.103		-0.002
		(0.389)		(0.169)		(0.151)
Non-German		-0.116		0.357		0.283
		(0.703)		(0.252)		(0.237)
Std. Errors	Heteroskedasticity-robust	Heteroskedasticity-robust	by address cluster	by address cluster	by address cluster	by address cluster
n	10,032	10,032	31,516	31,516	41,548	41,548

Estimates are expressed in terms of percentage points. Standard errors in parentheses. + p < 0.1, * p < 0.05, ** p < 0.01, *** p < 0.001.

© 2025 Ternovski J et al. *JAMA Network Open*

1.17 Regression Tables of Main Results

Table 44: Treatment effects. OLS regression estimates of the effects of the treatment on vaccinations.

	Direct effect		Spillover effect		Overall effect	
	(1) Unadjusted	(2) Adjusted	(3) Unadjusted	(4) Adjusted	(5) Unadjusted	(6) Adjusted
Treatment	-0.239	-0.247	-0.278	-0.269	-0.269+	-0.263+
	(0.269)	(0.269)	(0.170)	(0.169)	(0.149)	(0.148)
Age		0.033		0.014		0.017
		(0.048)		(0.023)		(0.021)
Age^2		0.000		0.000		0.000
		(0.000)		(0.000)		(0.000)
Female		0.112		-0.018		0.045
		(0.310)		(0.150)		(0.124)
Non-German		1.235*		0.961**		1.031***
		(0.587)		(0.304)		(0.278)
Std. Errors	Hetero-skedasticity-robust	Hetero-skedasticity-robust	by address cluster	by address cluster	by address cluster	by address cluster
n	10,032	10,032	31,516	31,516	41,548	41,548

Estimates are expressed in terms of percentage points. Standard errors in parentheses. + p < 0.1, * p < 0.05, ** p < 0.01, *** p < 0.001.

Table 45: Treatment effects by type of vaccination. Covariate-adjusted OLS regression estimates of the effects of the treatment on vaccinations. Effects are estimated for primary vaccinations (first and second dose) and booster vaccinations separately.

	Primary vaccination			Booster vaccination		
	(1) Direct Effect	(2) Spillover Effect	(3) Overall Effect	(4) Direct Effect	(5) Spillover Effect	(6) Overall Effect
Treatment	0.056	0.021	0.032	-0.319	-0.295*	-0.299**
	(0.142)	(0.124)	(0.103)	(0.233)	(0.118)	(0.108)
Age	-0.028	-0.026+	-0.026+	0.061	0.041*	0.043**
	(0.026)	(0.016)	(0.014)	(0.040)	(0.017)	(0.016)
Age^2	0.000	0.000	0.000	0.000	0.000	0.000
	(0.000)	(0.000)	(0.000)	(0.000)	(0.000)	(0.000)
Female	-0.159	-0.228*	-0.184*	0.279	0.208+	0.231*
	(0.171)	(0.099)	(0.079)	(0.264)	(0.115)	(0.097)
Non-German	1.483**	1.444***	1.460***	-0.224	-0.478***	-0.420**
	(0.457)	(0.269)	(0.246)	(0.380)	(0.142)	(0.132)
Std. Errors	Hetero-skedasticity-robust	by address cluster	by address cluster	Hetero-skedasticity-robust	by address cluster	by address cluster
n	9,896	31,163	41,059	9,982	31,259	41,241

Estimates are expressed in terms of percentage points. Standard errors in parentheses. + p < 0.1, * p < 0.05, ** p < 0.01, *** p < 0.001.

1.18 Pre-Registered Exploratory Analyses

Table 46: The interaction of treatment and nationality. Covariate-adjusted OLS regression estimates of the effects of the treatment on vaccinations and information uptake.

	(1) Direct effect Clicks	(2) Direct effect Vaccina-tions	(3) Spillover effect Vaccina-tions	(4) Overall effect Clicks	(5) Overall effect Vaccina-tions
Treatment	0.112	-0.205	-0.255	0.001	-0.248+
	(0.363)	(0.285)	(0.168)	(0.152)	(0.148)
Age	0.138*	0.033	0.014	0.140***	0.017
	(0.056)	(0.048)	(0.023)	(0.021)	(0.021)
Age^2	-0.001**	0.000	0.000	-0.001***	0.000
	(0.001)	(0.000)	(0.000)	(0.000)	(0.000)
Female	-0.162	0.110	-0.018	-0.002	0.045
	(0.389)	(0.310)	(0.150)	(0.151)	(0.124)
Non-German	-1.320	1.459+	1.001*	0.287	1.080*
	(0.948)	(0.876)	(0.486)	(0.334)	(0.445)
Treatment * Non-German	2.378+	-0.444	-0.081	-0.009	-0.099
	(1.310)	(1.208)	(0.620)	(0.448)	(0.565)
Std. Errors	Hetero-skedasticity-robust	Hetero-skedasticity-robust	by address cluster	by address cluster	by address cluster
n	10,032	10,032	31,516	41,548	41,548

Estimates are expressed in terms of percentage points. Standard errors in parentheses. + p < 0.1, * p < 0.05, ** p < 0.01, *** p < 0.001.

© 2025 Ternovski J et al. *JAMA Network Open*

Table 47: Effects of the treatment on first dose vaccinations. OLS regression.

	Direct effect		Spillover effect		Overall effect	
	(1) Unadjusted	(2) Adjusted	(3) Unadjusted	(4) Adjusted	(5) Unadjusted	(6) Adjusted
Treatment	0.054	0.051	-0.055	-0.041	-0.026	-0.017
	(0.124)	(0.124)	(0.124)	(0.119)	(0.101)	(0.098)
Age		-0.014		-0.001		-0.005
		(0.023)		(0.014)		(0.012)
Age^2		0.000		0.000		0.000
		(0.000)		(0.000)		(0.000)
Female		-0.237		-0.194*		-0.168*
		(0.148)		(0.088)		(0.071)
Non-German		1.014**		1.179***		1.159***
		(0.385)		(0.259)		(0.234)
Std. Errors	Heteroskedasticity-robust	Heteroskedasticity-robust	by address cluster	by address cluster	by address cluster	by address cluster
n	9,884	9,884	31,114	31,114	40,998	40,998

Estimates are expressed in terms of percentage points. Standard errors in parentheses. + p < 0.1, * p < 0.05, ** p < 0.01, *** p < 0.001.

We stated that we would attempt to map out the spread of letters given to others (from already fully-vaccinated to unvaccinated social contacts) via network analysis, but there were too few such transfers in our sample. Only 5 letters were given to other individuals who thereafter showed up to get vaccinated and presented the letters to vaccination officials. We do not pursue multinomial logistic regression for different types of vaccinations, as we pre-registered this analysis only in case of positive overall treatment effects. We also pre-registered that we would assess whether treatment affected residents' willingness to contact the city. Only 0.77% of citizens called the hotline of which only 27 could be matched with the main data, so we were underpowered to detect differences across conditions.

eAppendix 12. The Development and Prevalence of Evidence-Based Policy-Making

Before EBPM gained traction among policy-makers, the evidence-based medicine movement demonstrated the importance of going beyond doctors' expert opinions in a series of high-profile studies (Baron, 2018; Bothwell et al., 2016). From the outset, evidence-based medicine tended to value RCTs as the most preferred method for building evidence of intervention effectiveness (Baron, 2018). High profile cases included the Salk polio vaccine RCT conducted across ~130 U.S. schools, which eventually led to the eradication of polio in the country (American Academy of Pediatrics, 2022). The RCT was eventually made a legal requirement in many countries in the world as part of the standardized process for testing the efficacy and safety of new medical interventions (Bothwell et al., 2016).

Policy RCTs existed in academia decades before the term EBPM was coined,[5] but EBPM, and its prioritization of RCTs, began to coalesce into its modern form when influential governments began to codify EBPM into law and establish new EBPM institutions (McGann et al., 2018). In the US, the Education Science Reform Act of 2002 established the Institute for Educational Sciences, which worked with social scientists to measure educational program efficacy (Whitehurst, 2018). The Institute prioritized RCTs and, unsurprisingly, the number of educational RCTs grew eightfold between 2002 and 2016 (Connolly et al., 2018). Concurrently, non-government organizations, such as J-PAL, came to specialize in evaluating policies in the context of international development and rapidly grew in size and prominence; the number of impact evaluations conducted by J-PAL grew by almost an order of magnitude between 2002 and 2010 (de Souza Leão & Eyal, 2019). In 2010, the UK government created the Behavioral Insights Team (BIT) (John, 2014), which specialized in RCTs to evaluate policy effectiveness. The US followed suit shortly after; in 2015 the White House established the Social and Behavioral Sciences Team, which now resides within the US's General Services Administration and has been renamed as the Office of Evaluation Sciences (OES) (Office of Evaluation Science, 2023a). However, perhaps one of the prominent examples of the influence of EBPM in government came in the form of the Evidence Act. In 2018, a highly-polarized US Congress passed one of the few influential, overwhelmingly-bipartisan pieces of legislation in recent times: The Foundations for Evidence-based Policymaking Act of 2018. This law compelled all federal agencies to develop program evaluation capacity and to rigorously evaluate programs and policies (Office of Evaluation Science, 2023b).

Although, initially, there was considerable controversy as to what research counts as "evidence" in EBPM (Heinrich, 2007), because of RCTs prominence in evidence-based medicine, they eventually came to play a central role in EBPM as well (Baron, 2018; DellaVigna et al., 2022; Hjort et al., 2021). Many governments across the world have established public and private EBPM institutions that championed RCTs as the "gold standard" method for evaluating the causal impact of policies and programs (see, behavioral insights teams (Mukherjee & Giest, 2020) and public sector innovation labs (McGann et al., 2018).[6] And while alternative research methods have been used within the

[5]Early forms of EBPM included social policy, welfare, employment and education RCTs (Baron, 2018).

[6]A partial list of these organizations includes BIT in the UK (Haynes et al., 2012), the Fonds d'experimentation pour la jeunesse in France (Ministère de l'Éducation nationale, 2023), Ontario Behavioural Insights Unit in Canada (Government of Ontario, 2023), MineduLab in Peru (J-PAL, 2023), the Behavioural Economics Team and the New South Wales Behavioural Insights Unit in Australia, eMBeD at the World Bank (World Bank, 2023). In the US, organizations like OES and the White House's Office of Science and Technology Policy (Office of Evaluation Science, 2023c; The White House, 2014) prioritize RCTs on the federal level, but there is also a medley of state and local government equivalents such as the California Policy Lab or The Lab @ DC. In the Global South, private

context of EBPM and highlighted in prominent policy documents (The White House, 2020), the RCT has generally been the dominant research method for building evidence of policy effectiveness (Cairney & Oliver, 2017; DellaVigna et al., 2022; Evidence-Based Policymaking Collaborative, 2016; Monaghan & Ingold, 2019; Pearce & Raman, 2014).[7]

There is no shortage of EBPM process guides targeting policy-makers; many of these guides mention RCTs as part of the impact evaluation stage and their value in building evidence in favor or against a particular policy (Al-Akhali, 2020; Evidence-Based Policymaking Collaborative, 2016; The Pew Charitable Trusts & MacArthur Foundation, 2014; Tseng, 2015; Zanti & Thomas, 2021). However, the possibility of followup evaluation after scaling is infrequently mentioned and when it is, as with, for example, by the Evidence-Based Policymaking Collaborative (2016), there is little guidance as to the concrete steps a policy-maker should take to incorporate evaluation when scaling up a policy intervention.[8] And while recent works, such as (List, 2022) have focused on the possible pitfalls of scaling policy, the many different approaches to evaluating whether a policy has successfully scaled may be too technical for policy-makers. As such, since the simplicity of the RCT has helped it become a commonly-used tool in EBPM, we, similarly, propose incorporating the population-level RCT as a new step in the EBPM process before scaling up policy.

institutions such as the Busara Center for Behavioral Economics (headquartered in Kenya) (Busara, 2023) and the Center for Utilizing Behavioral Insights for Children (Save the Children International, 2023) work closely with governments to design, execute, and analyze policy RCTs (Silan et al., 2023).

[7]It's worth noting that the meta-analysis (Bothwell et al., 2016) and, more recently, the megastudy (Milkman et al., 2021) are considered by many as stronger evidence than any given RCT. However, both approaches usually require prohibitively more resources and/or much longer wait times before policy is implemented. As such, meta-analyses are not prominently featured in EBPM guides that target policymakers (Al-Akhali, 2020; Evidence-Based Policymaking Collaborative, 2016; The Pew Charitable Trusts & MacArthur Foundation, 2014; Zanti & Thomas, 2021) and are therefore outside of the scope of this paper.

[8]Contrast this with the plethora of step-by-step guides for conducting RCTs (Haynes et al., 2012).

eReferences

Al-Akhali, R. (2020, October 21). Using Evidence for Better Policymaking. Apolitical. https://apolitical.co/solution-articles/en/using-evidence-better-policymaking

American Academy of Pediatrics. (2022, June 28). The Poliomyelitis Vaccine Field Trial of 1954 Exhibit. AAP. https://www.aap.org/en/about-the-aap/gartner-pediatric-history-center/the-poliomyelitis-vaccine-field-trial-of-1954-exhibit

Atkinson, K. M., Mithani, S. S., Bell, C., Rubens-Augustson, T., & Wilson, K. (2020). The digital immunization system of the future: Imagining a patient-centric, interoperable immunization information system. Therapeutic Advances in Vaccines and Immunotherapy, 8, 2515135520967203.

Baron, J. (2018). A Brief History of Evidence-Based Policy. The ANNALS of the American Academy of Political and Social Science, 678(1), 40–50. https://doi.org/10.1177/0002716218763128

Bothwell, L. E., Greene, J. A., Podolsky, S. H., & Jones, D. S. (2016). Assessing the Gold Standard — Lessons from the History of RCTs. New England Journal of Medicine, 374(22), 2175–2181. https://doi.org/10.1056/NEJMms1604593

Busara Center for Behavioral Economics. (2023). Busara Center for Behavioral Economics. https://www.busara.global/

Cairney, P., & Oliver, K. (2017). Evidence-Based Policymaking Is Not Like Evidence-Based Medicine, so How Far Should You Go to Bridge the Divide Between Evidence and Policy? Health Research Policy and Systems, 15(1), 35. https://doi.org/10.1186/s12961-017-0192-x

Connolly, P., Keenan, C., & Urbanska, K. (2018). The Trials of Evidence-Based Practice in Education: A Systematic Review of Randomized Controlled Trials in Education Research 1980–2016. Educational Research, 60(3), 276–291. https://doi.org/10.1080/00131881.2018.1493353

DellaVigna, S., & Linos, E. (2022). RCTs to Scale: Comprehensive Evidence From Two Nudge Units. Econometrica, 90(1), 81–116. https://doi.org/10.3982/ECTA18709

Gerber, A. S., & Green, D. P. (2012). Field Experiments: Design, Analysis, and Interpretation New York: Norton

Government of Ontario. (2023, July 7). Behavioural Insights | Ontario.Ca. http://www.ontario.ca/page/behavioural-insights

Evidence-Based Policymaking Collaborative. (2016, September). Principles of Evidence-Based Policymaking. Urban Institute. https://www.urban.org/sites/default/files/publication/99739/principles_of_evidence-based_policymaking.pdf

Haynes, L., Service, O., Goldacre, B., & Torgerson, D. (2012). Test, Learn, Adapt: Developing Public Policy with Randomized Controlled Trials. SSRN Electronic Journal. https://doi.org/10.2139/ssrn.2131581

Heinrich, C. J. (2007). Evidence-Based Policy and Performance Management: Challenges and Prospects in Two Parallel Movements. The American Review of Public Administration, 37(3), 255–277. https://doi.org/10.1177/0275074007301957

© 2025 Ternovski J et al. *JAMA Network Open*

Hjort, J., Moreira, D., Rao, G., & Santini, J. F. (2021). How Research Affects Policy: Experimental Evidence from 2,150 Brazilian Municipalities. American Economic Review, 111(5), 1442–1480. https://doi.org/10.1257/aer.2019083

John, P. (2014). Policy Entrepreneurship in UK Central Government: The Behavioural Insights Team and the Use of Randomized Controlled Trials. Public Policy and Administration, 29(3), 257–267. https://doi.org/10.1177/0952076713509297

Lakens, D. (2013). Calculating and reporting effect sizes to facilitate cumulative science: a practical primer for t-tests and ANOVAs. *Frontiers in Psychology*, 4, 863.

Lakens, D. (2022). Sample size justification. *Collabra: Psychology*, 8(1), 33267.

Lakens, D., Scheel, A. M., & Isager, P. M. (2018). Equivalence testing for psychological research: A tutorial. *Advances in Methods and Practices in Psychological Science*, 1(2), 259-269.

List, J. A. (2022). The Voltage Effect. Currency.

McGann, M., Blomkamp, E., & Lewis, J. M. (2018). The Rise of Public Sector Innovation Labs: Experiments in Design Thinking for Policy. Policy Sciences, 51(3), 249–267. https://doi.org/10.1007/s11077-018-9315-7

Ministère de l'Éducation nationale. (2023). Le Fonds d'Expérimentation Pour La Jeunesse | Ministère de L'Éducation Nationale et de La Jeunesse. https://www.experimentation-fej.injep.fr/

Milkman, K. L., Gromet, D., Ho, H., Kay, J. S., Lee, T. W., Pandiloski, P., Park, Y., et al. (2021). Megastudies Improve the Impact of Applied Behavioural Science. Nature, 600(7889), 478–483. https://doi.org/10.1038/s41586-021-04128-4

Monaghan, M., & Ingold, J. (2019, April). Policy Practitioners' Accounts of Evidence-Based Policy Making: The Case of Universal Credit. Journal of Social Policy, 48(2), 351–368. https://doi.org/10.1017/S004727941800051X

Mukherjee, I., & Giest, S. (2020, November). Behavioural Insights Teams (BITs) and Policy Change: An Exploration of Impact, Location, and Temporality of Policy Advice. Administration & Society, 52(10), 1538–1561. https://doi.org/10.1177/0095399720918315

Office of Evaluation Science. (2023a). Evaluations | Office of Evaluation Sciences. https://oes.gsa.gov/work/

Office of Evaluation Science. (2023b). Evidence Act Toolkits | Office of Evaluation Sciences. https://oes.gsa.gov/toolkits/

Office of Evaluation Science. (2023c). OES Evaluation Policy. https://oes.gsa.gov/assets/files/evaluationpolicy.pdf

Papagiannis, D., Malli, F., & Gourgoulianis, K. I. (2022). Registry systems for COVID-19 vaccines and rate of acceptability for vaccination before and after availability of vaccines in 12 countries: a narrative review. Infectious Disease Reports, 14(1), 121–133.

Pearce, W., & Raman, S. (2014). The New Randomized Controlled Trials (RCT) Movement in Public Policy: Challenges of Epistemic Governance. Policy Sciences, 47(4), 387–402. https://doi.org/10.1007/s11077-014-9208-3

Save the Children International. (2023). CUBIC. https://www.savethechildren.net/cubic

Silan, M., Zelkowitz, A., Titus, B., Forscher, P., Gennari, F., Hickler, B., & Terer, M. (2023, April 27). Behavioral Insights in the Global South. APS Observer. https://www.psychologicalscience.org/observer/gs-behavioral-insights-global-south

Souza Leão, L. de, & Eyal, G. (2019). The Rise of Randomized Controlled Trials (RCTs) in International Development in Historical Perspective. Theory and Society, 48(3), 383–418.

The Abdul Latif Jameel Poverty Action Lab (J-PAL). (2023). MineduLAB. https://www.povertyactionlab.org/page/minedulab

Tseng, V. (2015, September). Studying the Use of Research Evidence in Policy & Practice. William T. Grant Foundation. https://wtgrantfoundation.org/wp-content/uploads/2015/09/Studying-the-Use-of-Research-Evidence-in-Policy-and-Practice.pdf

The White House. (2014, July 30). How Low-Cost Randomized Controlled Trials Can Drive Effective Social Spending. whitehouse.gov. https://obamawhitehouse.archives.gov/blog/2014/07/30/how-low-cost-randomized-controlled-trials-can-drive-effective-social-spending

The White House. (2020, March 10). Memorandum M-20-12 for Heads of Executive Departments and Agencies. Executive Office of the President - Office of Management and Budget. https://www.whitehouse.gov/wp-content/uploads/2020/03/M-20-12.pdf

The Pew Charitable Trusts, & MacArthur Foundation. (2014, November). Evidence-Based Policymaking: A Guide for Effective Government. https://www.pewtrusts.org/~/media/assets/2014/11/evidencebasedpolicymakingaguideforeffectivegovernment.pdf

Whitehurst, G. J. (2018). The Institute of Education Sciences: A Model for Federal Research Offices. The ANNALS of the American Academy of Political and Social Science, 678(1), 124–133. https://doi.org/10.1177/0002716218768243

World Bank. (2023). Mind, Behavior, and Development. https://www.worldbank.org/en/programs/embed

Zanti, S., & Thomas, M. L. (2021). Evidence-Based Policymaking: What Human Service Agencies Can Learn from Implementation Science and Integrated Data Systems. Global Implementation Research and Applications, 1(4), 304–314. https://doi.org/10.1007/s43477-021-00028-x
