# Supplementary material for: Financial Incentives for COVID-19 Vaccination: A Cluster Randomized Clinical Trial
Source: JAMA Netw Open. 2025 Feb 7;8(2):e2458542. doi: 10.1001/jamanetworkopen.2024.58542 (PMC11806395; doi:10.1001/jamanetworkopen.2024.58542)
Supplement: Supplement 3. — Data Sharing Statement [file jamanetwopen-e2458542-s003.pdf]

# Data Sharing Statement

Ternovski. Financial Incentives for COVID-19 Vaccination. *JAMA Netw Open*. Published February 10, 2025. doi:10.1001/jamanetworkopen.2024.58542

**Data**
**Additional Information:** ISRCTN, 59503725, doi.org/10.1186/ISRCTN59503725
**Data available:** Yes
**Data types:** Deidentified participant data
**How to access data:** https://osf.io/tepf6/
**When available:** With publication

**Supporting Documents**
**Document types:** Statistical/analytic code
**How to access documents:** https://osf.io/tepf6/
**When available:** With publication

**Additional Information**
**Who can access the data:** Publicly available online
**Types of analyses:** For any purpose
**Mechanisms of data availability:** Publicly available online
